# Supplementary material for: Resolving dynamics and function of transient states in single enzyme molecules
Source: Nat Commun. 2020 Mar 6;11:1231. doi: 10.1038/s41467-020-14886-w (PMC7060211; doi:10.1038/s41467-020-14886-w)
Supplement: Supplementary file 1 — Supplementary Information [file 41467_2020_14886_MOESM1_ESM.pdf]

# Supplementary Information

# Resolving dynamics and function of transient states in single enzyme molecules

Hugo Sanabria[1,2, 8,*], Dmitro Rodnin[1,8], Katherina Hemmen[1,8], Thomas Peulen[1], Suren Felekyan[1], Mark R Fleissner[3,7], Mykola Dimura[1,4], Felix Koberling[5], Ralf Kühnemuth[1], Wayne Hubbell[3], Holger Gohlke[4,6], Claus A.M. Seidel[1*]

[1]Institut für Physikalische Chemie, Lehrstuhl für Molekulare Physikalische Chemie, Heinrich-Heine-Universität, Düsseldorf, Germany.
[2]Department of Physics and Astronomy, Clemson University, Clemson, South Carolina, U.S.A.
[3]Jules Stein Eye Institute and Department of Chemistry and Biochemistry, University of California, Los Angeles, U.S.A.
[4]Institut für Pharmazeutische und Medizinische Chemie, Heinrich-Heine-Universität, Düsseldorf, Germany.
[5]PicoQuant GmbH, Berlin, Germany.
[6]John von Neumann Institute for Computing (NIC), Jülich Supercomputing Centre (JSC) & Institute of Biological Information Processing (IBI-7: Structural Biochemistry), Forschungszentrum Jülich GmbH, 52425 Jülich, Germany
[7]Present address: Avanir Pharmaceuticals Inc., Aliso Viejo, California, U. S. A.

[8]These authors contributed equally: Hugo Sanabria, Dmitro Rodnin, Katherina Hemmen

*Correspondence and requests for materials should be addressed to C.A.M.S.(email: cseidel@hhu.de) or to H.S. (email: hsanabr@clemson.edu)

# *Table of contents*


| | Title | Page |
|---|---|---|
| **Supplementary Figure 1** | Detecting protein dynamics with our fluorescence spectroscopic toolkit | **3** |
| **Supplementary Figure 2** | MFD analysis of all 33 variants of the T4L network | **4** |
| **Supplementary Figure 3** | Exemplary data analysis for variant K60pAcF I150C-(DA) | **7** |
| **Supplementary Figure 4** | Brownian Dynamic Simulations of S44pAcF/I150C-(DA) | **8** |
| **Supplementary Figure 5** | eTCSPC and EPR results of T4L | **9** |
| **Supplementary Figure 6** | Reduced FRET chi-squared values of the NMR and X-ray structures. | **12** |
| **Supplementary Figure 7** | Single-molecule experiments of functional variants | **13** |
| **Supplementary Figure 8** | T4L binding to peptidoglycan as observed by reverse phase HPLC and cleavage at low pH | **14** |
| **Supplementary Figure 9** | Exemplary filters for fFCS analysis and fFCS curves | **15** |
| **Supplementary Figure 10** | MFD analysis of further samples | **17** |
| **Supplementary Figure 11** | Total energy landscape of the hydrolysis of T4L on a generalized reaction coordinate | **18** |
| **Supplementary Figure 12** | Triplet or dark states do not influence the *sCCF* on the variant S44pAcF/I150C-(DA) | **19** |
| **Supplementary Table 1** | States description for the vector $p$, equilibrium faction vector $p_{eq}$ and the rate matrix $K$ | **20** |
| **Supplementary Table 2** | eTCSPC fit results | **21** |
| **Supplementary Table 3** | Analysis of time-resolved fluorescence anisotropies $r(t)$ | **30** |
| **Supplementary Table 4** | Conformational clusters of T4L PDB structures | **35** |
| **Supplementary Table 5** | List of evaluated fit models | **36** |
| **Supplementary Table 6** | List of primers used for cloning | **37** |
| **Supplementary Note 1** | Single-molecule and fluorescence correlation spectroscopy | **38** |
| **Supplementary Note 2** | Species Cross Correlation Function -(DA) and –(AD) labeled samples. | **39** |
| **Supplementary Note 3** | Analyzing the kinetic network of conformational states in T4L | **40** |
| **Supplementary Note 4** | Fluorescence decay analysis of single and double labeled T4 Lysozyme | **43** |
| **Supplementary Note 5** | Characterization of functional T4L variants | **44** |
| **Supplementary Note 6** | Challenges of smFRET measurements and their solutions | **44** |
| **Supplementary Note 7** | Original data available on Zenodo | **47** |
| **Supplementary Methods** | • Multiparameter Fluorescence Detection (MFD)<br>• MFD burst analysis: Multiparameter FRET histograms and FRET-lines<br>• Guidelines for reading MFD histograms<br>• Filtered Fluorescence Correlation Spectroscopy<br>• Ensemble Time Correlated Single Photon Counting with high precision<br>• Donor and Acceptor fluorescence quantum yields<br>• Time-resolved fluorescence decay analysis | **50** |

# *Supplementary Figures*

## Supplementary Figure 1

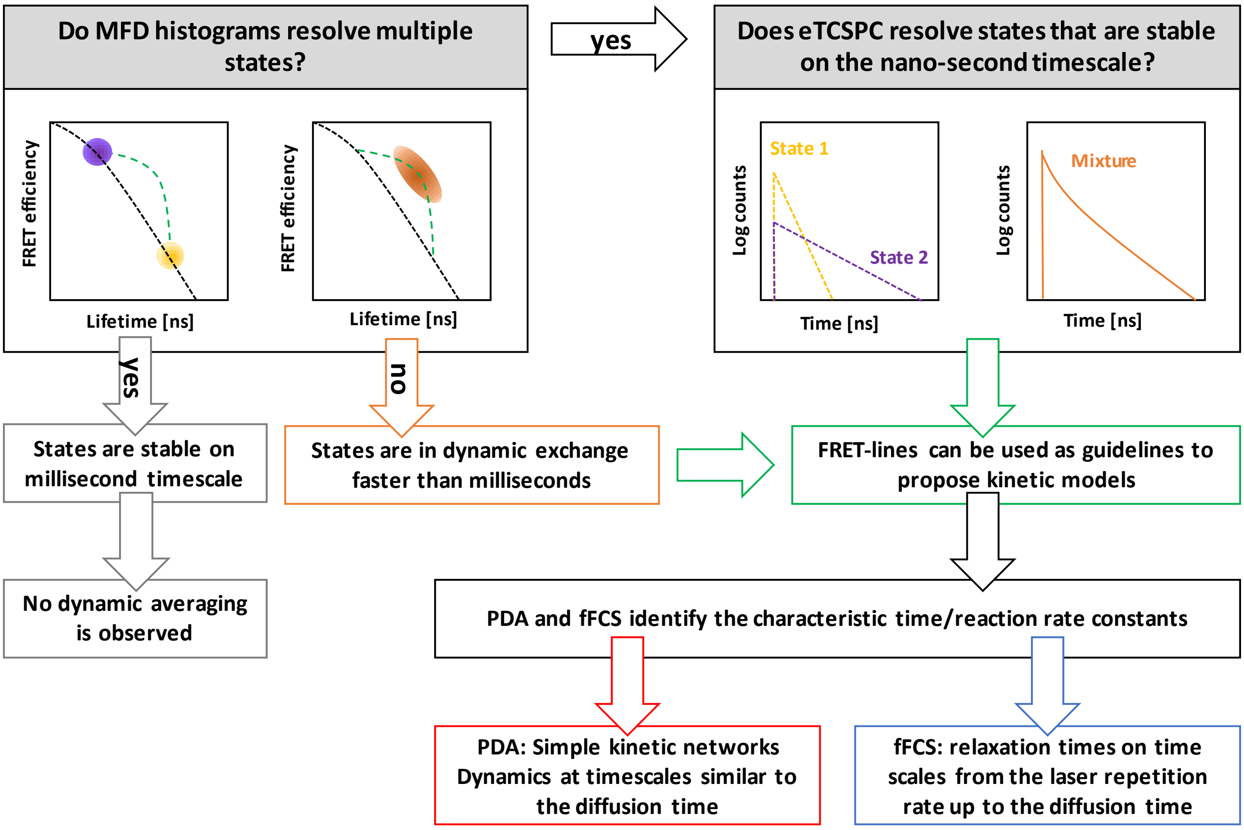


**Supporting Figure 1** How to identify and analysis dynamics with our fluorescence spectroscopic toolkit. Multiparameter Fluorescence Detection (MFD) immediately shows whether a sample undergoes dynamic exchange and the timescale of dynamics can be estimated by the position of the population(s) with respect to the static FRET line. Populations, which are stable on the nanosecond timescale are resolved ensemble time-correlated single-photon counting (TCSPC). These states obtained in TCSPC can be used to generate static and dynamic FRET lines, which serve as guidelines to propose kinetic models. (Filtered) Fluorescence correlation spectroscopy is good in identifying the relaxation times on time scales from the laser repetition rate up to the diffusion time, providing a broader dynamic range, while Photon Distribution Analysis (PDA) is useful for simple and kinetic networks and for dynamics that occur at timescales similar to the diffusion time.

## Supplementary Figure 2

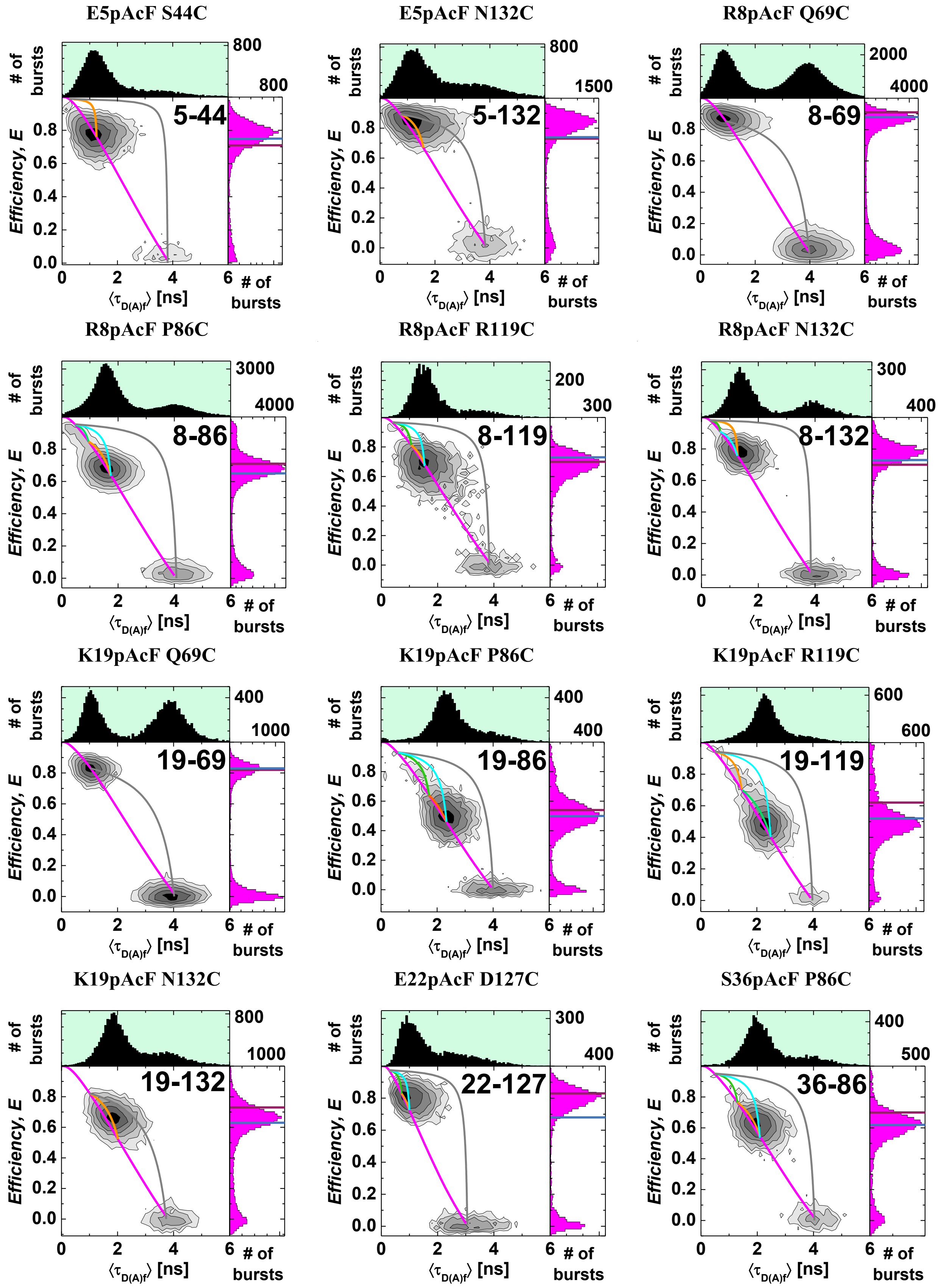

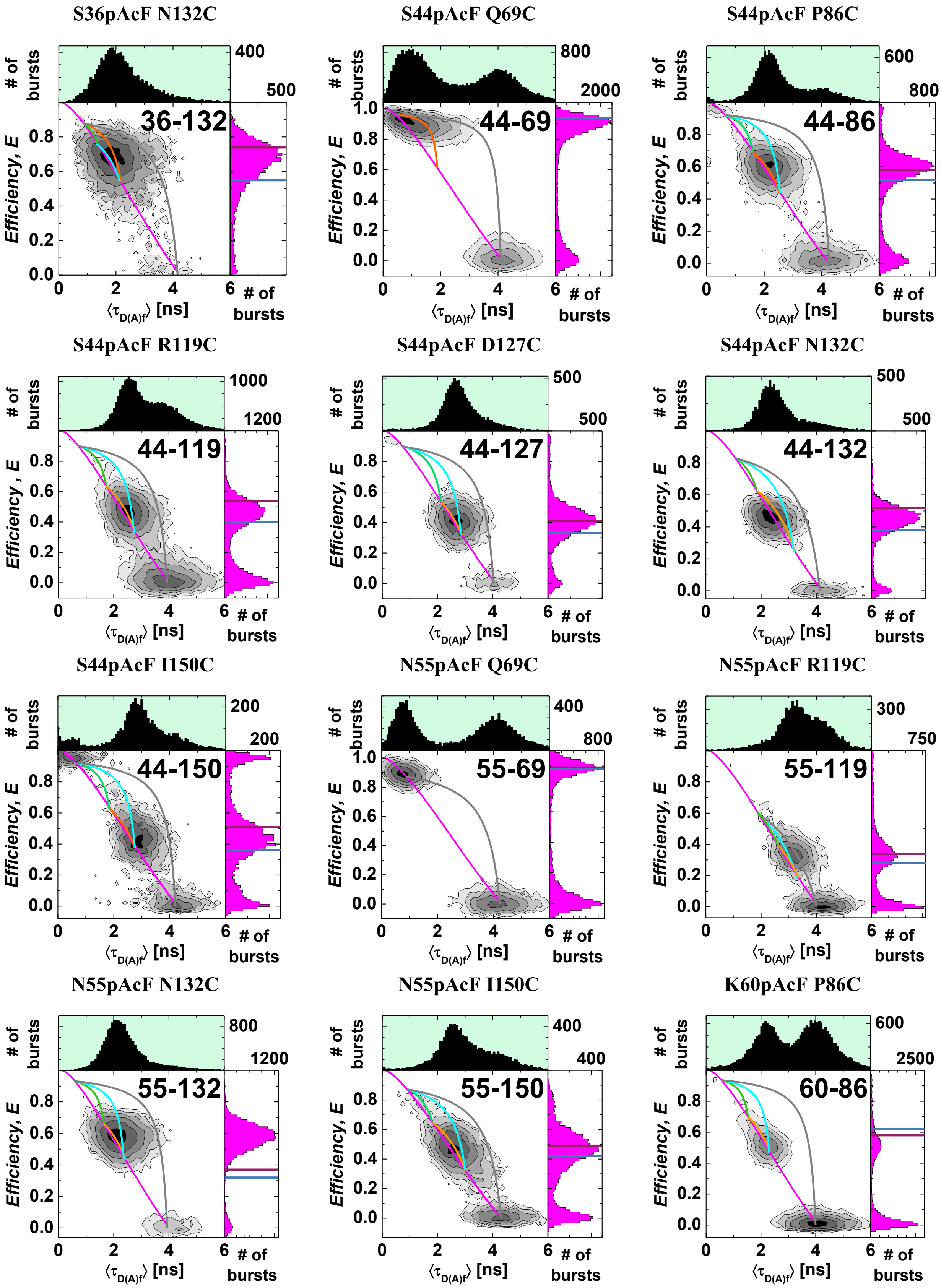
S36pAcF N132C
36-132
S44pAcF Q69C
44-69
S44pAcF P86C
44-86
S44pAcF R119C
44-119
S44pAcF D127C
44-127
S44pAcF N132C
44-132
S44pAcF I150C
44-150
N55pAcF Q69C
55-69
N55pAcF R119C
55-119
N55pAcF N132C
55-132
N55pAcF I150C
55-150
K60pAcF P86C
60-86
# of bursts
Efficiency, E
⟨τ_D(A)f⟩ [ns]

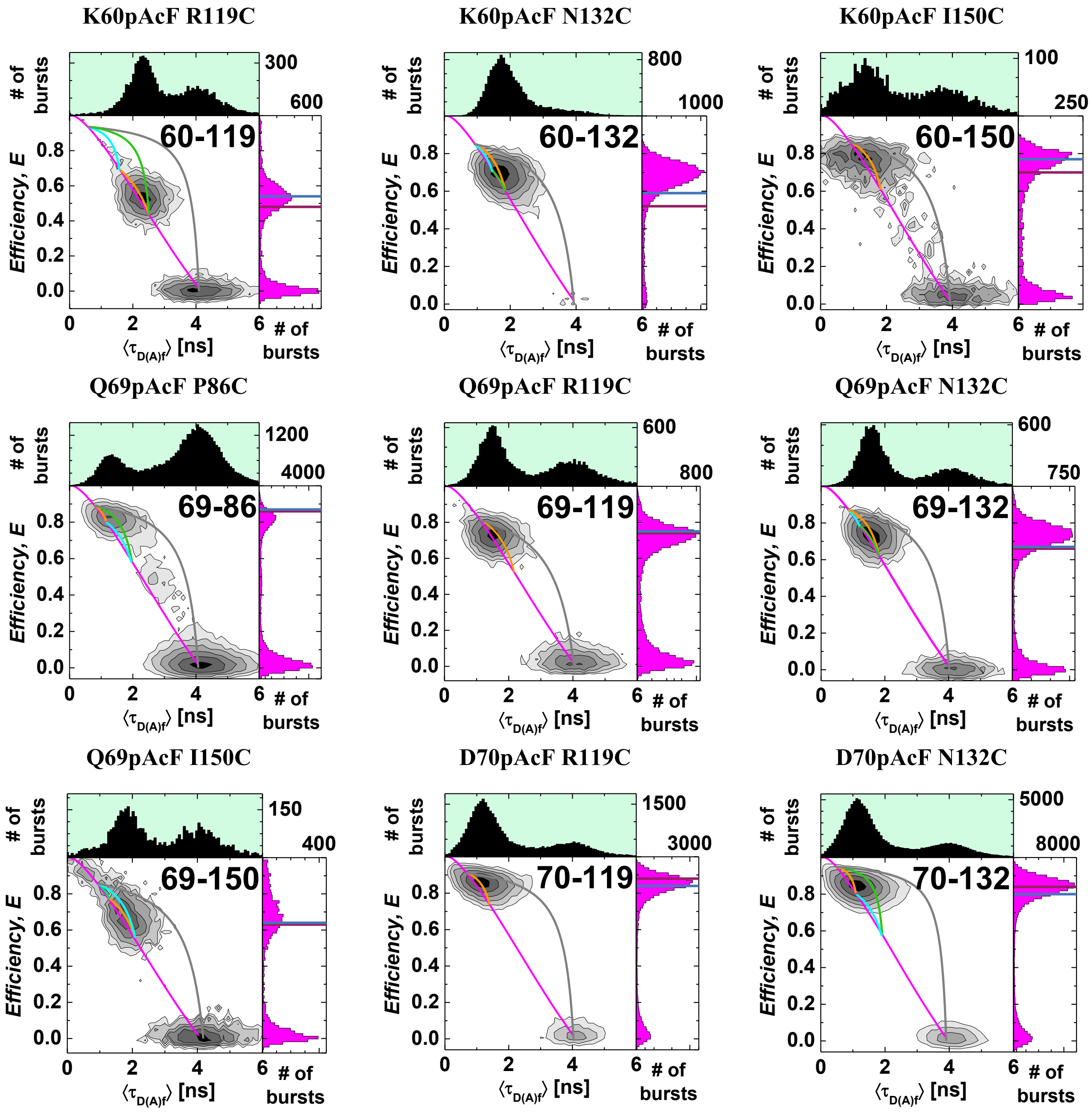


**Supplementary Figure 2. MFD analysis of all 33 variants of the T4L network.** Two dimensional histogram of FRET efficiency $E$ vs. lifetime of donor in the presence of acceptor $\langle\tau_{D(A)}\rangle_f$. One dimensional projections for $E$ and $\langle\tau_{D(A)}\rangle_f$ are also shown. Static (magenta) and dynamic FRET lines connecting states $C_1$-$C_2$ (orange), $C_1$-$C_3$ (cyan) and $C_2$-$C_3$ (bright green) are also shown (Supplementary Methods, Supplementary Equations (19-21)). Solid horizontal lines show the FRET efficiency expected from known X-ray structures for the *open* (blue, PDB ID 172L) and *closed* (violet, 148L) state from T4L.

**Supplementary Figure 3**

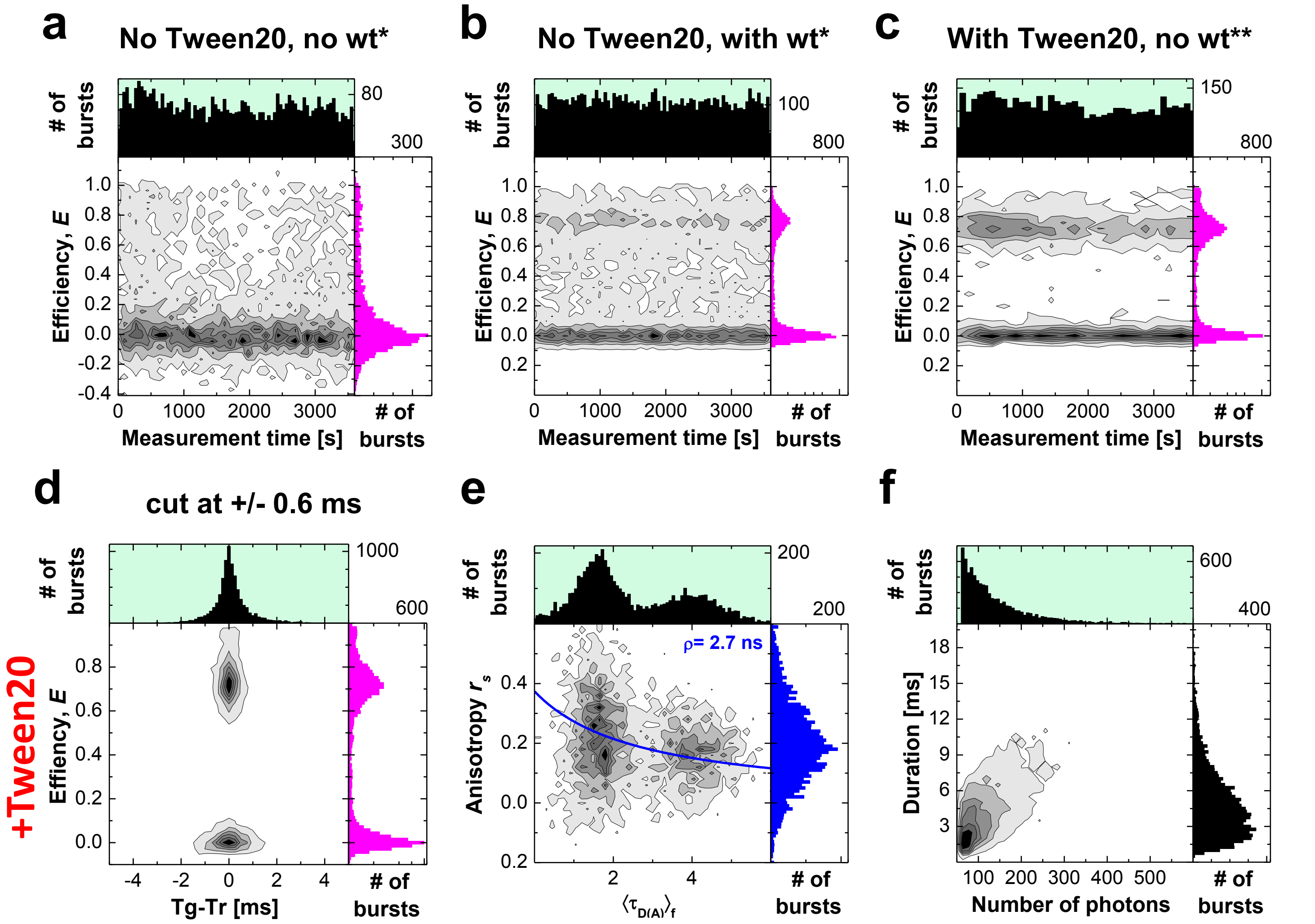


**Supplementary Figure 3. Exemplary data analysis for variant K60pAcF I150C-(DA). (a-c)** FRET efficiency E vs. the measurement time for 60-150 in the absence of coating (A), coating of the measurement chamber with 0.1 % (v/v) Tween20 (b), or 1 µM unlabeled protein (c). **(d)** In the case of acceptor photobleaching, the green signal trace will be longer (Tg) than the red signal trace (Tr). The difference |Tg-Tr| is in ideal case randomly and sharply distributed around 0. Molecules, in which |Tg-Tr| exceeds 0.6 are removed from further analysis. **(e)** Free dye molecules can be recognized by a low anisotropy ~ 0, labeled single molecules follow the Perrin equation. **(f)** Aggregates will show a high brightness and long burst duration, which do not scale linearly.

## Supplementary Figure 4

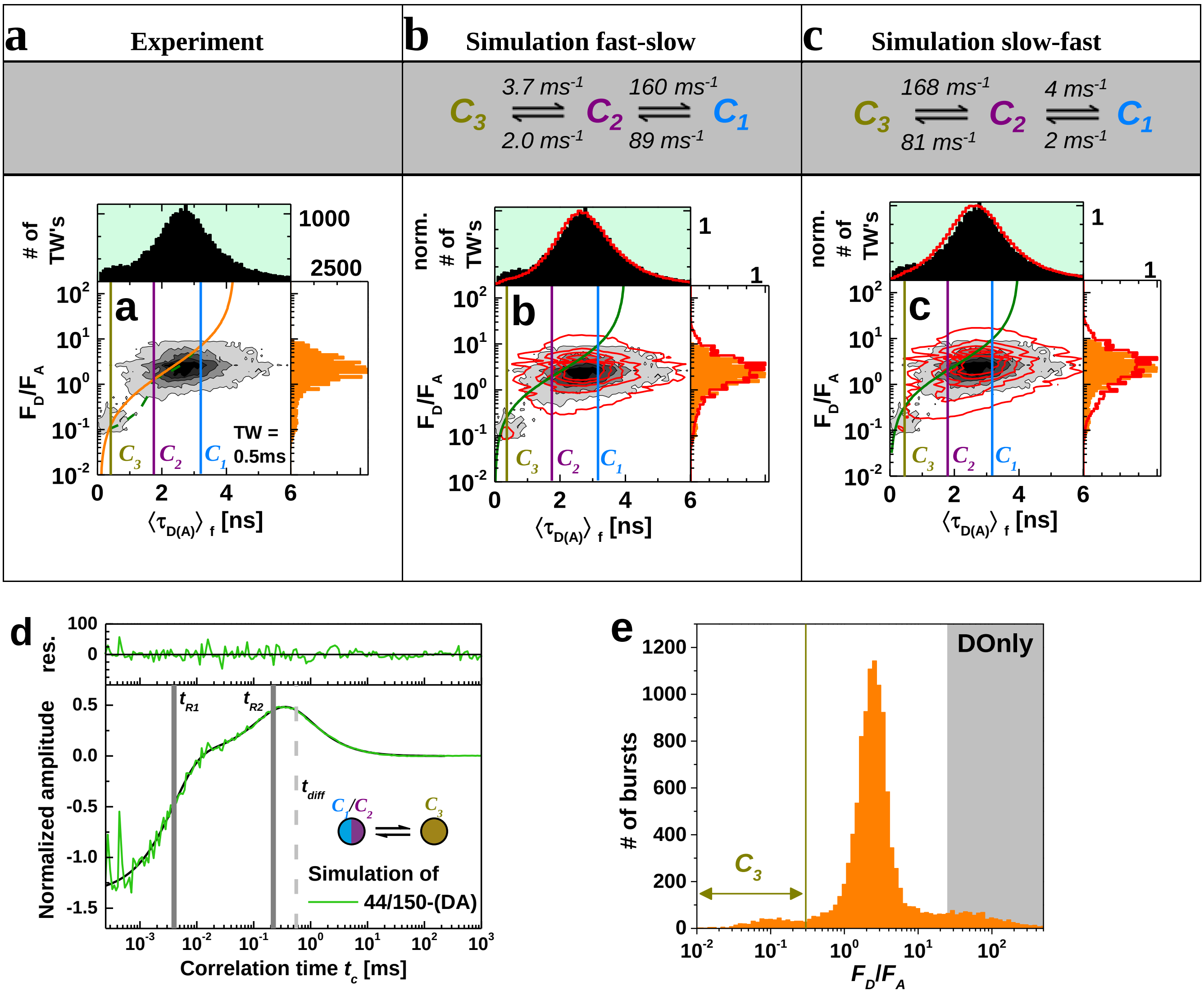


**Supplementary Figure 4. Brownian Dynamics (BD) Simulations of S44pAcF/I150C-(DA). (a-c)** The experimental data (a) are compared with data from BD simulation for the depicted kinetic schemes (b,c). (**a**) Two dimensional histograms from smFRET analysis ($F_D/F_A$ vs. fluorescence lifetime ($\langle\tau_{D(A)}\rangle_f$) of the raw data from the S44pAcF/I150-(DA) variant at pH 7.5 selected with 0.5 ms time-windows (TW's). FRET lines, static and dynamic are shown as orange solid and green dashed lines. $\langle B_G\rangle$ = 1.6 kHz, $\langle B_R\rangle$ = 0.8 kHz, spectral crosstalk $\alpha$ = 1.2% and ratio of green and red detection efficiencies $g_G/g_R$ = 0.77 are used for corrections. (**b, c**) Brownian dynamics simulation using the rates from Figure 3b, c was processed as the experimental data (overlaid red contours). Simulated parameters ($\langle B_G\rangle$, $\langle B_R\rangle$, $\alpha$, $g_G/g_R$) were the same as in the experiment. In addition, we considered a rotational correlation of $\rho$ = 2.2 ns for conformational state. Analysis results of simulated data are presented in the same fashion as in panel (a). The experimental data agree best with simulated data for the case "fast-slow" (b). (**d**) *sCCF* between the pseudo-species consisting of the $C_1/C_2$ mix and the $C_3$ for simulated data. Fit of this *sCCF* curve returns two relaxation times of 4 µs and 220 µs, consistent with our input parameters. **(e)** One dimensional histogram of the raw data from the S44pAcF/I150-(DA) variant at pH 7.5 analyzed in burstwise mode to illustrate the region of $C_{3t}$, D-Only and the dynamically mixed state.

# Supplementary Figure 5

**a**

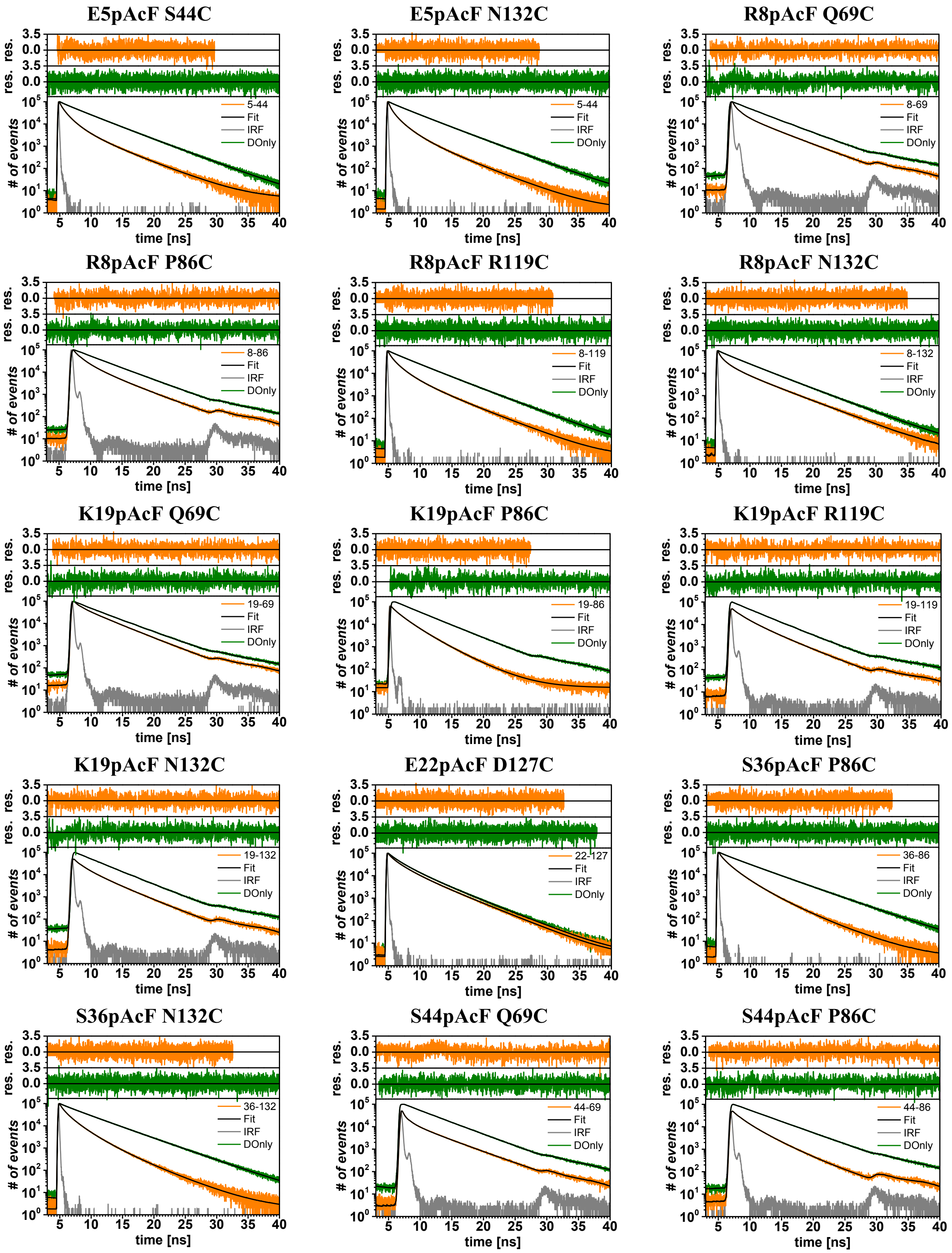

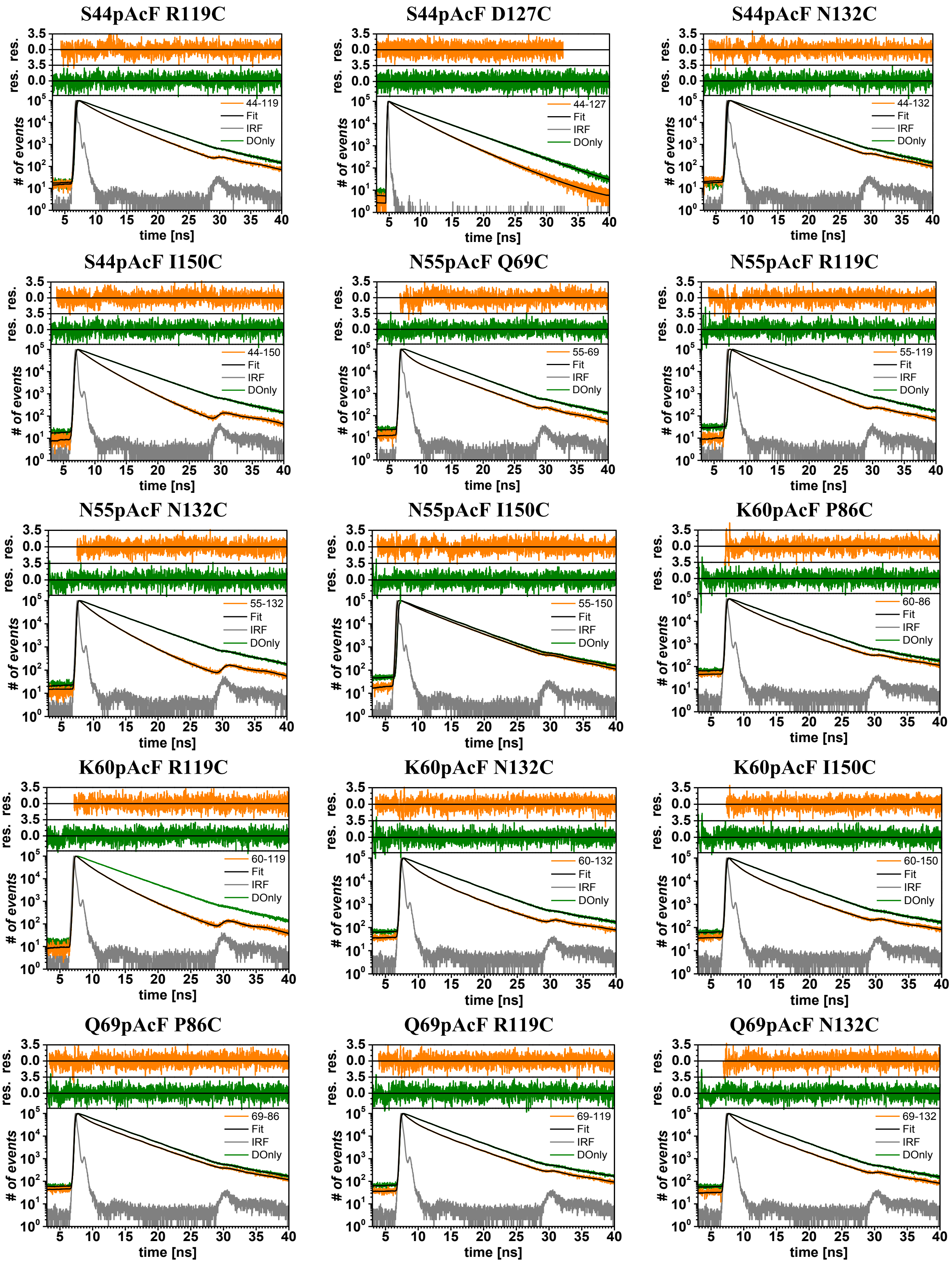
S44pAcF R119C
44-119
S44pAcF D127C
44-127
S44pAcF N132C
44-132
S44pAcF I150C
44-150
N55pAcF Q69C
55-69
N55pAcF R119C
55-119
N55pAcF N132C
55-132
N55pAcF I150C
55-150
K60pAcF P86C
60-86
K60pAcF R119C
60-119
K60pAcF N132C
60-132
K60pAcF I150C
60-150
Q69pAcF P86C
69-86
Q69pAcF R119C
69-119
Q69pAcF N132C
69-132
Fit
IRF
DOnly
res.
# of events
time [ns]

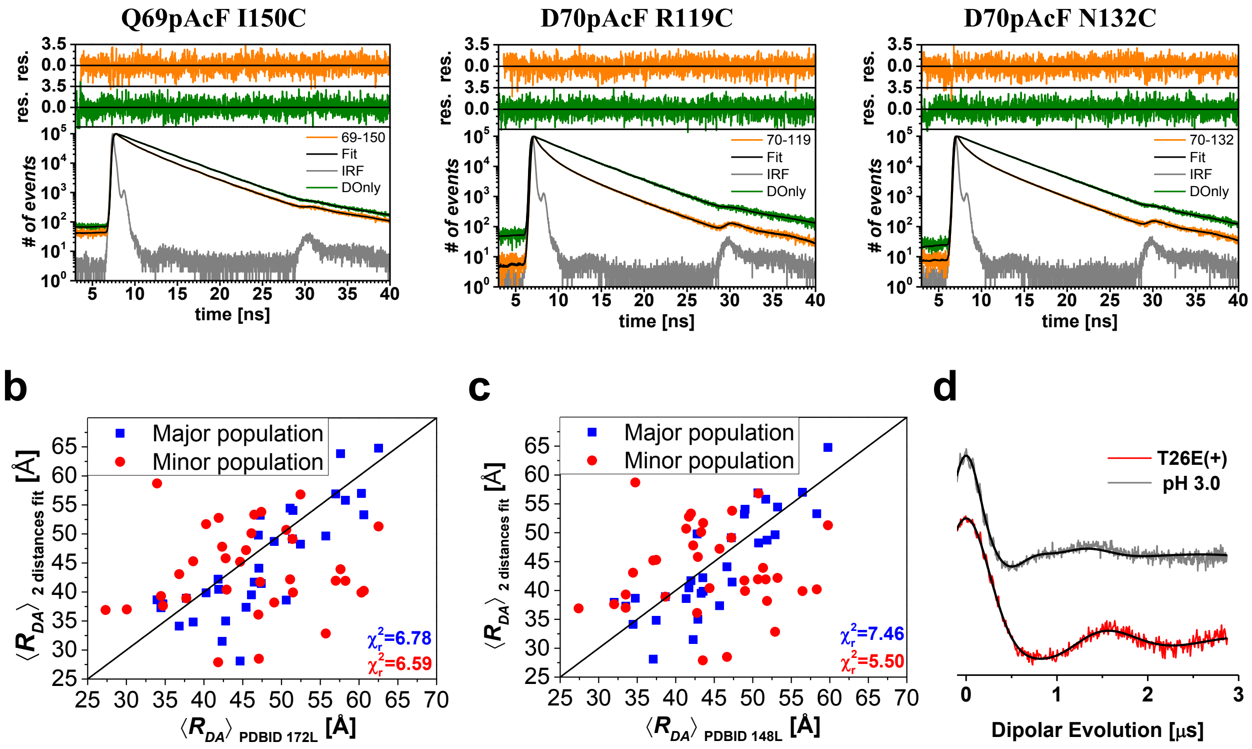


**Supplementary Figure 5. eTCSPC results of T4L. (a)** Fit (black) of the experimental data of double-labeled sample (orange) and respective Donor-only labeled sample (green), weighted residuals are shown on top. Fit parameter are given in Table S2a (D-Only) and S2d-f (double-labeled sample). Instrument response function (IRF) is shown in gray. **(b)** Fitted distances of two distributed states (Table S2b) fit plotted versus the distances calculated for the model X-ray structure of the open state (PDBID: 172L). “Major state” is the distance having the higher amplitude in fraction, while “minor state” is the distance with the lower fraction. **(c)** Same as (b), only for the model X-ray structure of the closed state (PDBID 148L). **(d)** Experimental DEER time traces of the dipolar evolution, which were used to calculate the distance distributions shown in the main text Figure 6a. For pH 3.0 data (grey) the modulation depth was 0.33, the maximum time 2.87 µs, the upper distance limit 56 Å, and the upper shape limit 45 Å. For the T26E adduct data (red), the modulation depth was 0.39, the maximum time 2.88 µs, the upper distance limit also 56 Å, and the upper shape limit also 45 Å. Fits are overlaid.

## Supplementary Figure 6

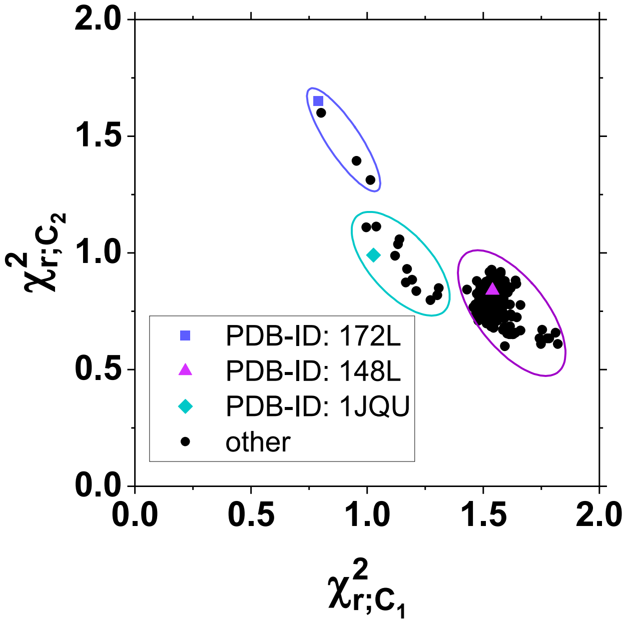


**Supplementary Figure 6. Reduced FRET chi-squared values of the NMR and X-ray structures.** The X axis represents the $\chi^2_{r,C_1}$ of the PDB structures against the FRET dataset $C_1$, the Y axis represents the $\chi^2_{r,C_2}$. The $C_1$ representative structure (open, PDB-ID: 172L) is shown as blue rectangle, similar structures -are highlighted by the blue ellipse. The $C_2$ representative is shown as violet triangle (closed, PDB-ID: 148L), similar structures are highlighted by the violet ellipse. The ajar structure (PDB-ID: 1JQU) is shown as a light blue diamond, similar structures are highlighted by the light blue ellipse. The data are available at Zenodo (see Supplementary Note 7) in the file FRET_screening_of_PDB_structures.zip.

## Supplementary Figure 7

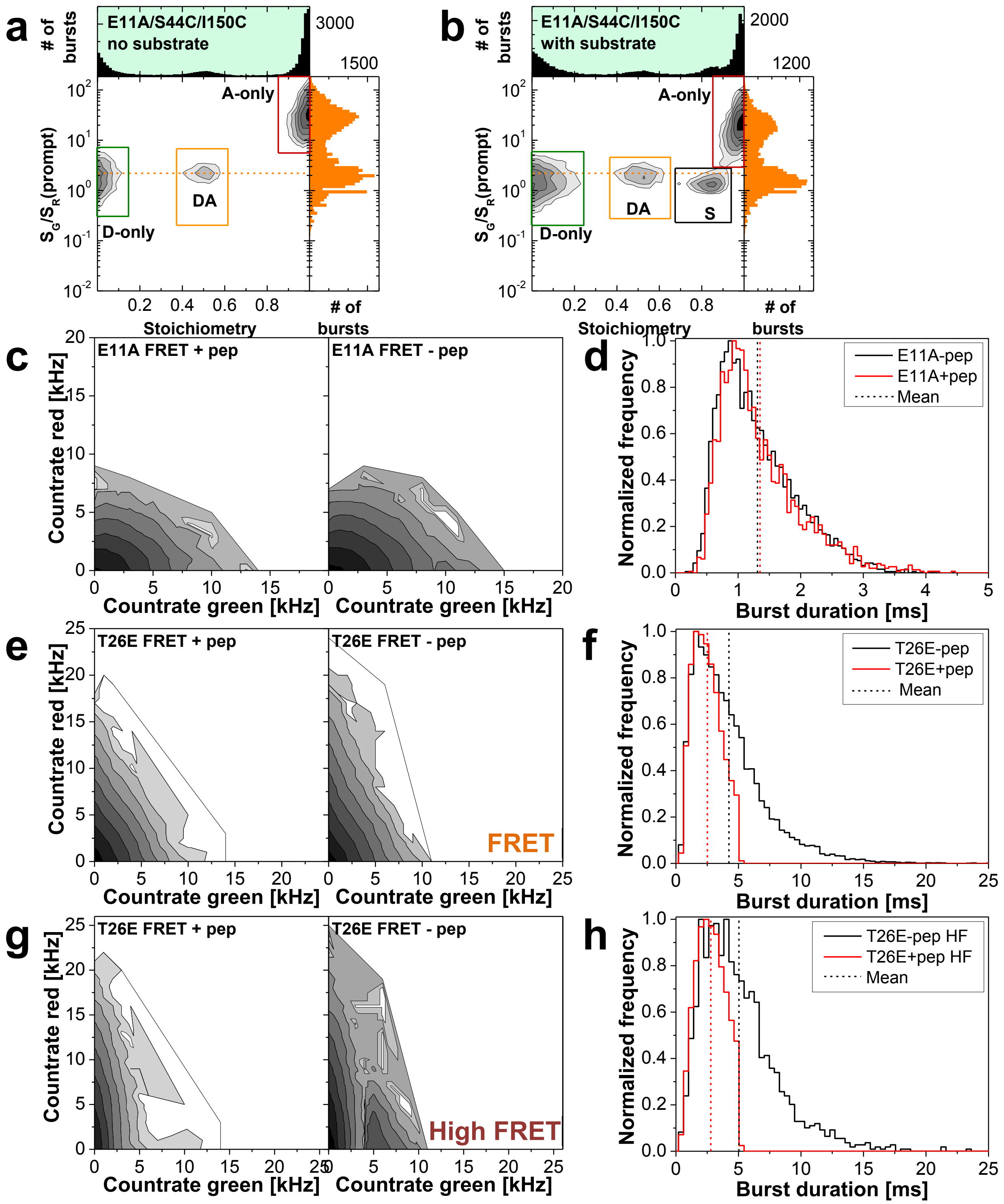


**Supplementary Figure 7. Single-molecule experiments of functional variants. (a,b)** PIE experiments identify burst stemming from single and double labeled E11A/S44C/I150C and substrate alone. **(c)** Brightness distribution of bursts from double-labeled E11A/S44C/I150C. **(d)** Burst duration distribution of bursts from double-labeled E11A/S44C/I150C. **(e-h)** Same as (c,d) for the variant T26E/S44pAcF/I150C-(DA).

## Supplementary Figure 8

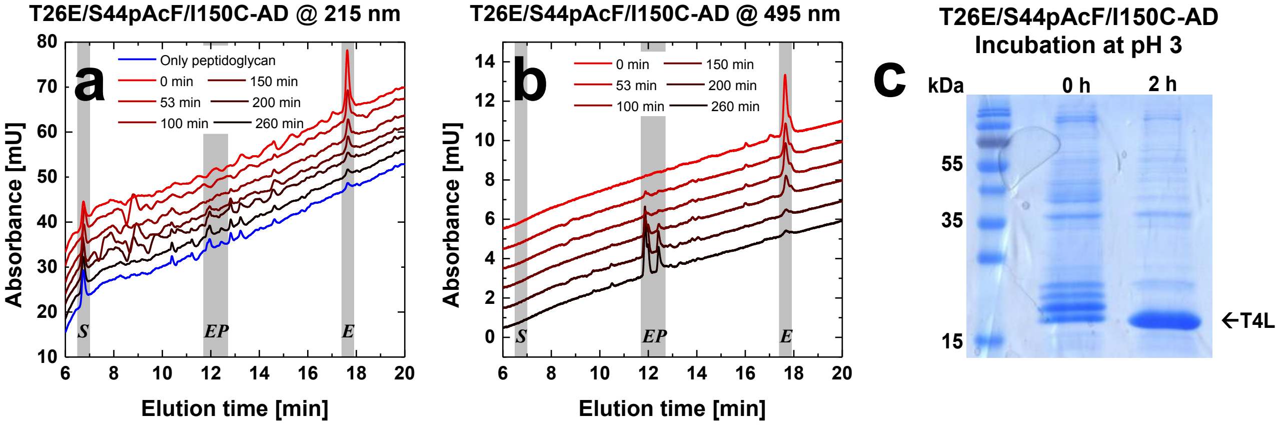


**Supplementary Figure 8. T4L binding to peptidoglycan as observed by reverse phase HPLC and cleavage at low pH. (a)** The elution profile measured at 215 nm of reverse phase chromatography for T26E/S44pAcF/I150C-(AD). Samples were taken at different times during the incubation with peptidoglycan. First line shows only the elution of peptidoglycan. Note different peaks of the heterogeneity on the peptidoglycan. Offset between lines was added for clarity. Gray lines represent the free enzyme population (*e*), the product bound enzyme (*EP*) and the substrate alone (*S*). **(b)** Elution of the same sample as in (a) but monitored at 495 nm, which corresponds to the absorbance of Alexa488. Saturation of T26E/S44pAcF/I150C-(AD) with substrate is reached at ~ 4 hours of incubation. **(c)** Purification of T26E/S44pAcF/I150C from the *E. coli* cell pellet yielded a mixture of free and to cell wall pieces of different sizes bound protein. After incubation for 2 hrs at pH 3, nearly all bound peptidoglycan had been cleaved and the free enzyme could now be used for labeling and further experiments after adjusting the conditions to neutral pH again. Source Data are provided by a source data file.

**Supplementary Figure 9**

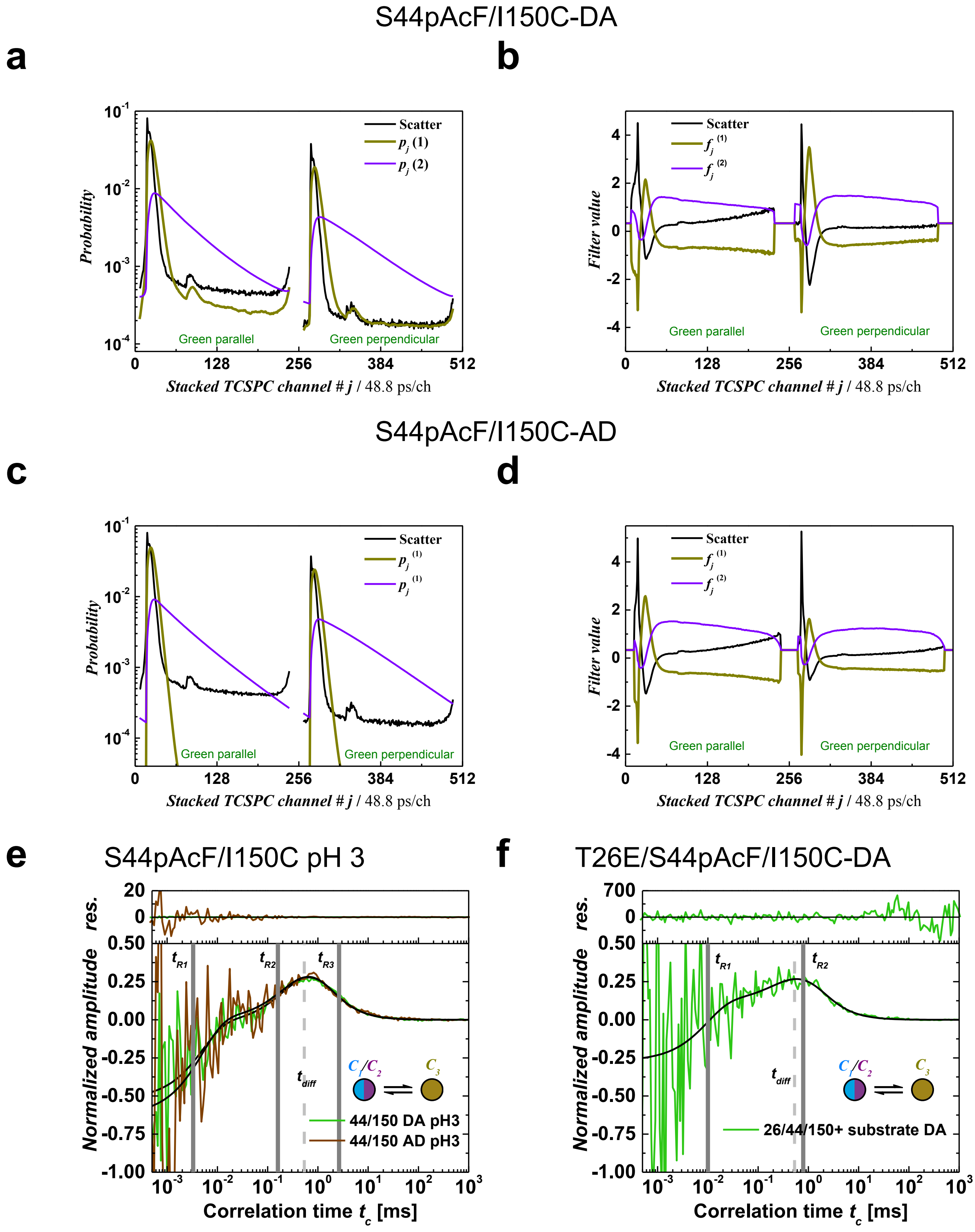

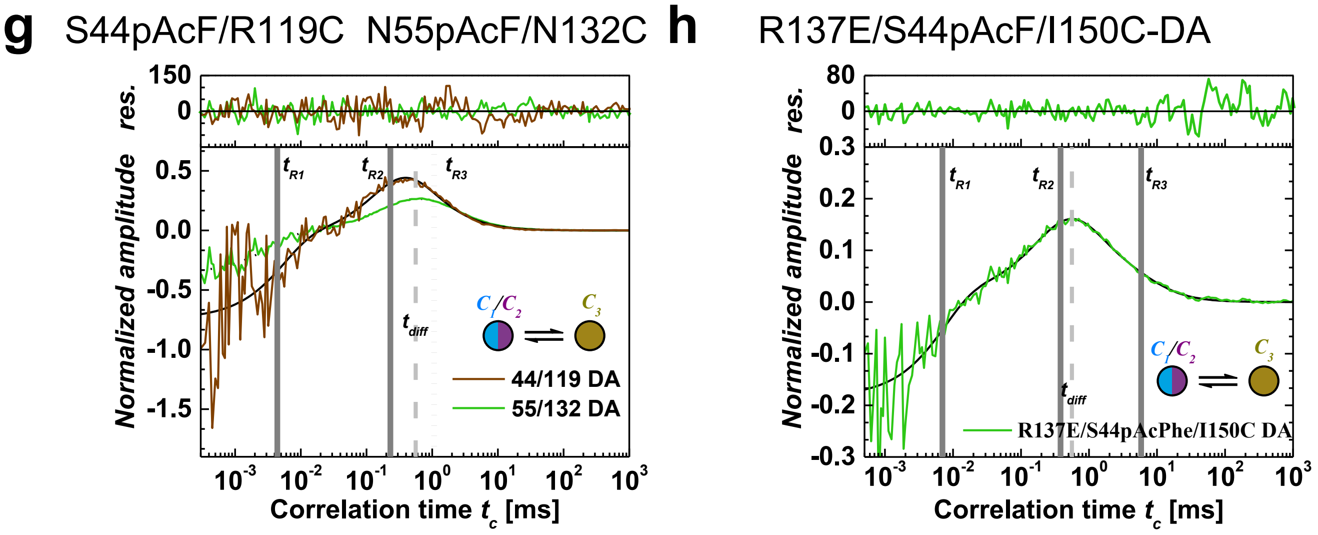


**Supplementary Figure 9. fFCS results. (a)** Generated decays for two pseudo-species of S44pAcF/I150C-DA in addition to the scatter profile. The parameters of the decay generation for the first pseudo-species were $\tau_1$ = 0.25 ns, and rotational correlation time of $\rho_1$ = 3.3 ns. The second pseudo-species had a lifetime of $\tau_2$ = 2.97 ns and the same rotational correlation time. **(b)** Filters $f_j^{(i)}$ were calculated according to Supplementary Equation 26 using the decays from graph (A). **(c, d)** Decay patterns and the corresponding filters for the S44pAcF/I150C-AD with first pseudo species lifetime $\tau_1$ = 0.25 ns, and rotational correlation time $\rho_1$ = 3.3 ns. The second pseudo species was generated with $\tau_2$ = 3.25 ns and same rotational correlation time. **(e)** *sCCF* between the mix $C_1/C_2$ and $C_3$ at pH 3.0 for the two configurations of labeling –(DA) and –(AD). The fit with the Supplementary Equations (27-28) required three relaxation times. The diffusion time was fixed to $t_{diff}$ = 0.54 ms. **(f)** *sCCF* between the mix $C_1/C_2$ and $C_3$ for T26E/S44pAcF/I150C-(DA) incubated with substrate. Two relaxation times are found ($t_{R1}$= 10 µs, and $t_{R2}$ = 0.790 ms). **(g)** Overlay of the normalized *sCCF* of S44pAcF/R119C-DA and N55pAcF/N132C-(DA). Global fit shows two common relaxation times ($t_{R1}$ = 4 ± 2.4 µs, $t_{R2}$ = 230 ± 28 µs). The variant N55pAcF/N132C-(DA) requires an additional rate $t_{R3}$ ~ 1.1 ms. **(h)** *sCCF* for variant R137E/S44pAcF/I150C-(DA). Three relaxation times were needed to fit the curve ($t_{R1}$ = 7 µs, $t_{R2}$ = 0.38 ms and $t_{R3}$ = 5.84 ms). The diffusion time was fixed to $t_{diff}$ = 0.54 ms.

## Supplementary Figure 10

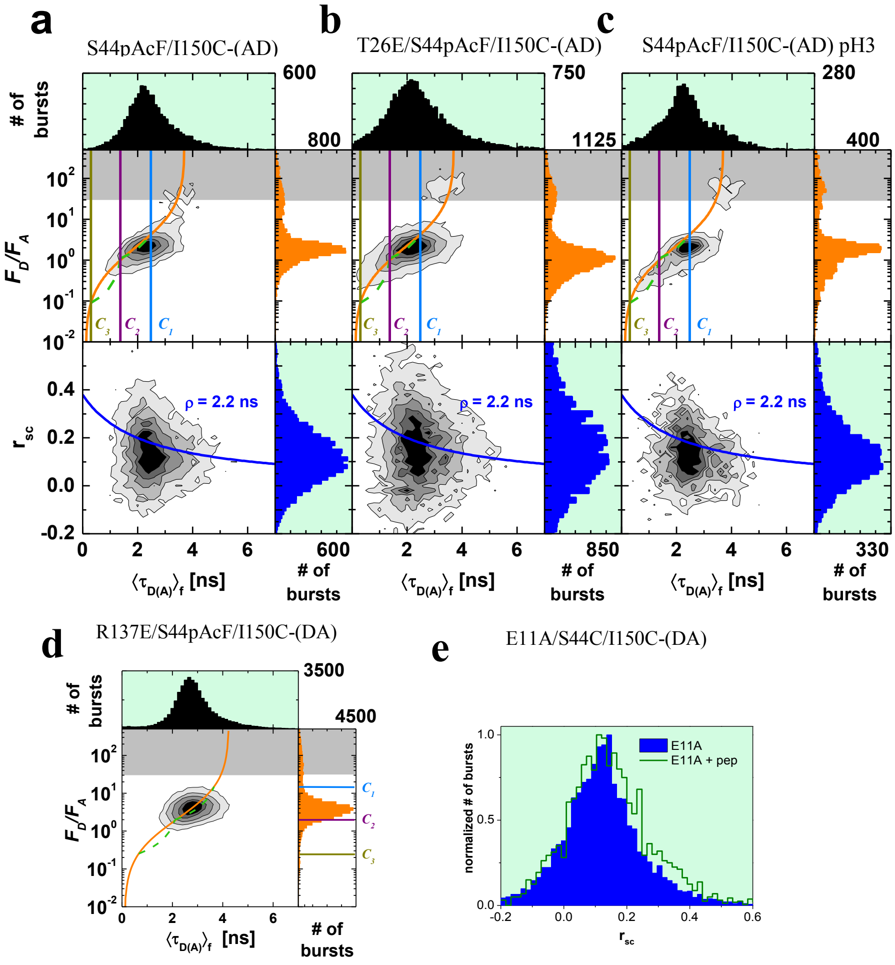


**Supplementary Figure 10. MFD analysis of further samples. (a)** MFD histogram of S44pAcF/I150C-(AD) labeled T4L. Two dimensional histogram $F_D/F_A$ vs. lifetime of donor in the presence of acceptor $\langle\tau_{D(A)}\rangle_f$, and anisotropy vs. $\langle\tau_{D(A)}\rangle_f$ for S44pAcF/I150C-(AD). One dimensional projections for $F_D/F_A$, $\langle\tau_{D(A)}\rangle_f$ and anisotropy are also shown. Static FRET line is shown in orange. Pure donor and acceptor fluorescence ($F_D$ and $F_A$) are corrected for background ($\langle B_G\rangle$ = 1.8 kHz, $\langle B_R\rangle$ = 0.7 kHz), spectral cross-talk ($\alpha$ = 1.3%) and detection efficiency ratio ($g_G/g_R$ = 0.77). Shaded area in gray is the region of donor only. On the anisotropy vs. $\langle\tau_{D(A)}\rangle_f$ histograms the Perrin's equation with rotational correlation $\rho$ = 2.2 ns is shown as blue line. Vertical guidelines for states $C_1$, $C_2$, and $C_3$ according to the eTCSPC results of the same sample are added as references. Ignoring the donor only population a single unimodal distribution is observed in all $F_D/F_A$ vs. $\langle\tau_{D(A)}\rangle_f$; similarly to what was observed in the –(DA) sample. Two slight differences can be observed: the tilt towards the state $C_3$ is more evident and the accumulation of the $C_{3t}$ is not visible. **(b)** MFD histograms for the variant T26E/S44pAcF/I150C-(AD) without substrate. We observe a more pronounced broadening along the FRET-line in direction to $C_3$. **(c)** At pH 3.0, the MFD histograms for the S44pAcF/I150C-(AD) show very similar characteristics as the variant T26E. **(d)** Functional mutant of T4L**.** MFD histograms for R137E/S44pAcPh/I150C-(DA). **(e)** Effect of substrate on E11A/S44C/I150C. Upon addition of substrate we observe a higher anisotropy (green line). All samples were corrected for background, cross talk, and detection efficiencies according to experimentally determined parameters.

## Supplementary Figure 11

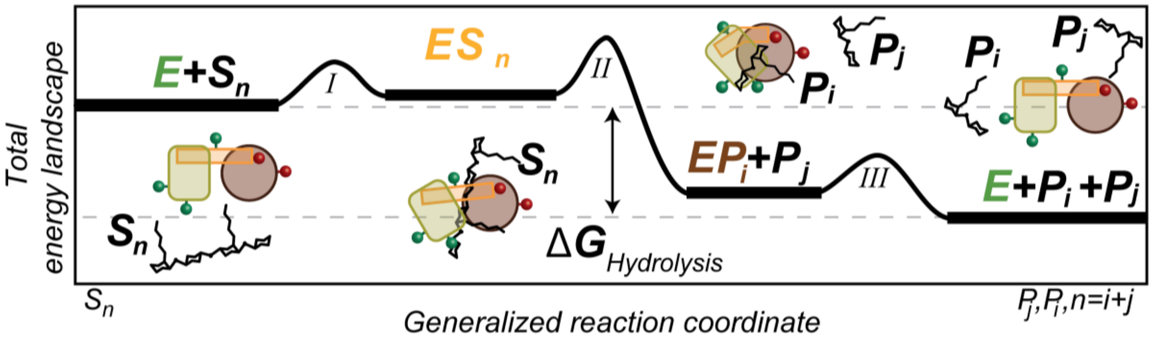


**Supplementary Figure 11. Total energy landscape of the hydrolysis of T4L on a generalized reaction coordinate.** T4L cleaves the polymer chain peptidoglycan, the substrate *S*, of length $n$ ($S_n$) between the alternating residues of β-(1,4) linked N-acetylglucosamine and N-acetylmuramic acid into two shorter peptidoglycan chains, the product *P* of chain length $i$ and $j$ ($P_i$ and $P_j$). Here, $n = i + j$.

The enzymatic pathway of the extended Michaelis-Menten mechanism (Fig. 7a in main text) consists of three distinct reaction steps. *I*) The substrate *S* binds reversibly to the enzyme *E* to form an enzyme-substrate complex *ES*. *II*) In the *ES* complex, *S* is converted to the product *P*, resulting in the *EP* complex with the product still bound to the enzyme. *III*) *P* is released from the complex via a transition of *E* to an excited state $E^*$. Finally, the free enzyme $E^*$ relaxes to *E*. Our observations demonstrate that a fine-tuned shift of the conformational equilibrium favors motions of active product release in T4L where the energy of product formation in step II defines the directionality of the reaction [1]. This hydrolysis reaction is irreversible and thus can be denoted as "ratchet mechanism" [2].

## Supplementary Figure 12

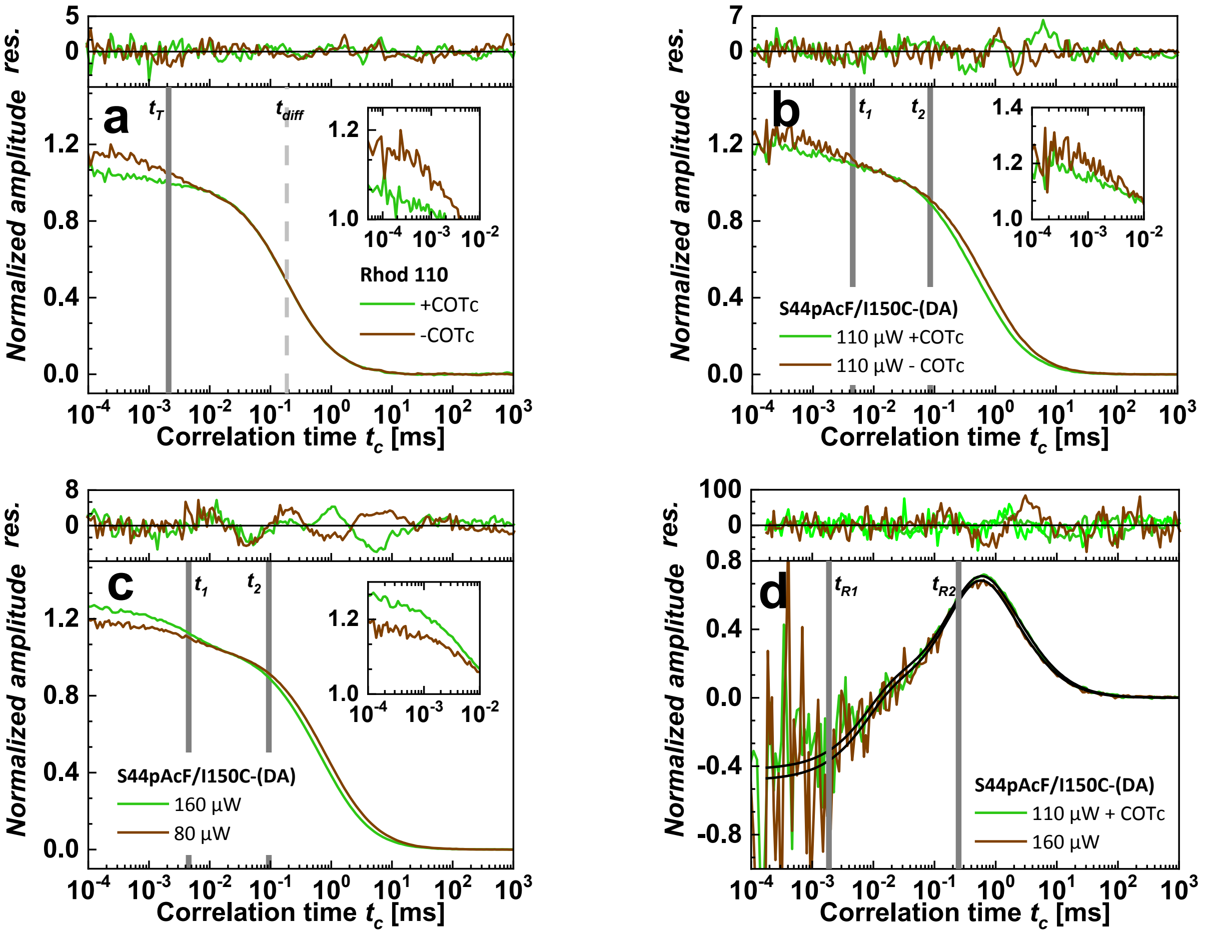


**Supplementary Figure 12. Triplet or dark states do not influence the *sCCF* on the variant S44pAcF/I150C-(DA).** **(a)** The addition of the triplet quencher COTc into Rhod110 solution significantly reduces triplet fraction (see in inset). **(b)** Overlay of the standard auto/cross-correlation curves from signals in the green channels for the variant S44pAcF/I150C-(DA) without (-COTc) and with (+COTc) triplet quencher COTc in solution. Inset shows the regime where triple kinetics is observed. **(c)** Overlay of standard auto/cross-correlation of the green signals at 80 µW and at 160 µW power at objective. Two bunching terms are needed to fit the data ($t_T$ = 4.5 µs, and $t_b$ = 60 µs). The triplet fraction changes from 10 % at 80 µW to 15 % at 160 µW power at objective. Also changes in diffusion times are observed from 0.8 ms at 80 µW to 0.6 ms at 160 µW power at objective. Photobleaching can account for this change. Inset shows the reduction of the triplet fraction by COTc quencher. **(d)** *sCCF* of the variant S44pAcF/I150C-(DA) between pseudo-species $C_1/C_2$ and $C_3$ at different power at 80 µW and at 160 µW power at the objective. The relaxation times fitted globally are $t_{R1}$ = 6 µs and $t_{R2}$ = 240 µs, that are within the errors presented on Table S4c. Note that the amplitudes do not change as in the case of the standard auto-correlation.

# *Supplementary Tables*

## Supplementary Table 1

**Supplementary Table 1a**. States description for the vector ***p***, equilibrium faction vector $\boldsymbol{p_{eq}}$ and the rate matrix ***K***.

| Kinetic state i | State name | Efficiency *E* | Fluorescence lifetime $\tau$, ns [a] | Equilibrium fraction $p_{eq,i}$ |
|---|---|---|---|---|
| 1 | $C_1$ | 0.2 | 3.2 | 0.51 |
| 2 | $C_2$ | 0.5 | 2.0 | 0.29 |
| 3 | $C_3$ | 0.9 | 0.5 | 0.14 |
| 4 | $C_{3d}$ | 0.9 | 0.5 | 0.06 |

$$\boldsymbol{K}_{fastslow} = \begin{pmatrix} -158.1 & 90.8 & 0 & 0 \\ 158.1 & -94.5 & 1.8 & 0 \\ 0 & 3.7 & -1.808 & 0.003 \\ 0 & 0 & 0.008 & -0.003 \end{pmatrix} \quad \boldsymbol{K}_{slowfast} = \begin{pmatrix} -3.5 & 2 & 0 & 0 \\ 3.5 & -169.7 & 81.1 & 0 \\ 0 & 167.7 & -81.108 & 0.003 \\ 0 & 0 & 0.008 & -0.003 \end{pmatrix}$$

**Supplementary Table 1b.** Parameters used in the evaluation of the statistical significance of different simulations.

| Type of analysis | $\chi^2$ fast-slow | $\chi^2$ slow-fast | Degrees of freedom | *F-value* | $p(C_1$-$C_2$ fast vs. $C_1$-$C_2$ slow) |
|---|---|---|---|---|---|
| 1D $\langle\tau_{D(A)}\rangle_f$ histogram | 68.6 | 187.2 | 10 | 2.72 | 1 |
| 1D *E* histogram | 85.1 | 95.5 | 10 | 1.12 | 0.734 |
| 2D $\langle\tau_{D(A)}\rangle_f$ vs *E* histogram | 0.4551 | 0.6069 | 10 | 1.33 | 1 |

**Supplementary Table 1c.** Calculated reaction rates for several variants using Supplementary Equation (8). Confidence intervals (2σ) are shown in squared brackets and the corresponding renormalized fractions shown below $x_1$+$x_2$+$x_{3d}$ =1*.

| **Samples** | $k_{12}$ **[ms$^{-1}$]** | $k_{21}$ **[ms$^{-1}$]** | $k_{23}$ **[ms$^{-1}$]** | $k_{32}$ **[ms$^{-1}$]** |
|---|---|---|---|---|
| 44/150-(DA) | 89<br>[45.5-175.6] | 160<br>[74.1-312.7] | 2.0<br>[1.5-2.3] | 3.7<br>[3.1-4.3] |
| 11/44/150-(DA)+pept | 217<br>[134.8-535.4] | 33<br>[19.0-86.38] | 0.8<br>[0.6-1.1] | 3.7<br>[3.1-4.3] |
| 26/44/150-(DA) + pept | 79<br>[49.2-193.3] | 21<br>[13.0-52.6] | 0.5<br>[0.4-0.6] | 0.9<br>[0.8-1.0] |

The relaxations times used were: $t_{R1}$ = 4 ± 2.3 µs; $t_{R2}$ = 230 ± 28.4µs for 44/150-(DA) and 11/44/150-(DA)+pept. $t_{R1}$ = 10 µs; $t_{R2}$ = 790 µs (Fig. S6F) was used for 26/44/150-(DA) + pept.

| **Chemical State** | **Samples** | $x_1$ | $x_2$ | $x_{3d}$ |
|---|---|---|---|---|
| *E* | 44/150-(DA) | 0.54 | 0.30 | 0.16 |
| *ES* | 11/44/150-(DA)+pept | 0.30 | 0.54 | 0.16 |
| *EP* | 26/44/150-(DA) +pept | 0.35 | 0.29 | 0.36 |

* Rounded to 2 digits. Renormalized fractions based on the relative changes observed in all states in the presence of substrate (Fig. 6E). Only the amino acid number of the mutagenesis is shown.

## Supplementary Table 2

**Supplementary Table 2a.** Fluorescence properties of the dyes without FRET. The table lists the parameters of the multi-exponential fits (Supplementary Equation 32) of Donor-only labeled variants and of the direct acceptor excitation of double labeled FRET samples. Fluorescence lifetimes $\tau_i$ and corresponding species fractions $x_i$ and fluorescence quantum yields of the donor ($\Phi_{FD(0)}$, Alexa488) and acceptor ($\Phi_{FA}$, Alexa647) are listed. Empty cells represent parameters that are not applicable. The donor fluorophore was attached to the unnatural amino acid pAcF, the acceptor fluorophore to the Cysteine residue.

| Variant | $\tau_1$ [ns] | $x_1$ | $\tau_2$ [ns] | $x_2$ | $\tau_3$ [ns] | $x_3$ | $\langle\tau\rangle_{x,D(0)}$ [ns] | $\Phi_{FD(0)}$ † | $\langle\tau\rangle_{x,A}$ [ns] | $\Phi_{FA}$ |
|---|---|---|---|---|---|---|---|---|---|---|
| E5pAcF/S44C | 4.10 | 0.77 | 2.44 | 0.17 | 0.43 | 0.06 | 3.59 | 0.78 | 1.24 | 0.36 |
| E5pAcF/N132C | 4.11 | 0.78 | 2.33 | 0.16 | 0.34 | 0.06 | 3.59 | 0.78 | 1.25 | 0.37 |
| R8pAcF/Q69C | 4.10 | 0.89 | 1.60 | 0.11 | | | 3.83 | 0.78 | 1.23 | 0.34 |
| R8pAcF/P86C | 4.16 | 0.92 | 1.89 | 0.08 | | | 3.98 | 0.79 | 1.35 | 0.40 |
| R8pAcF/R119C | 4.03 | 0.77 | 2.43 | 0.17 | 0.38 | 0.05 | 3.54 | 0.75 | 1.26 | 0.34 |
| R8pAcF/N132C | 4.08 | 0.79 | 2.27 | 0.14 | 0.34 | 0.07 | 3.55 | 0.76 | 1.25 | 0.34 |
| K19pAcF/Q69C | 4.25 | 0.78 | 2.22 | 0.13 | 0.65 | 0.09 | 3.60 | 0.80 | 1.22 | 0.33 |
| K19pAcF/P86C | 3.90 | 0.77 | 2.56 | 0.15 | 0.32 | 0.08 | 3.39 | 0.73 | 1.30 | 0.38 |
| K19pAcF/R119C | 3.91 | 0.74 | 2.51 | 0.15 | 0.52 | 0.11 | 3.27 | 0.73 | 1.32 | 0.39 |
| K19pAcF/N132C | 3.91 | 0.81 | 2.66 | 0.14 | 0.32 | 0.05 | 3.54 | 0.74 | 1.20 | 0.33 |
| E22pAcF/D127C | 3.83 | 0.36 | 1.83 | 0.47 | 0.67 | 0.17 | 2.25 | 0.60 | 1.24 | 0.34 |
| S36pAcF/P86C | 4.40 | 0.81 | 2.28 | 0.14 | 0.49 | 0.05 | 3.89 | 0.83 | 1.30 | 0.39 |
| S36pAcF/N132C | 4.39 | 0.83 | 2.23 | 0.13 | 0.46 | 0.05 | 3.91 | 0.83 | 1.25 | 0.34 |
| S44pAcF/Q69C | 4.32 | 0.94 | 1.75 | 0.06 | | | 4.17 | 0.83 | 1.25 | 0.34 |
| S44pAcF/P86C | 4.32 | 0.94 | 1.75 | 0.06 | | | 4.17 | 0.83 | 1.26 | 0.35 |
| S44pAcF/R119C | 4.32 | 0.96 | 1.44 | 0.04 | | | 4.20 | 0.84 | 1.27 | 0.35 |
| S44pAcF/D127C | 4.28 | 0.85 | 2.25 | 0.10 | 0.39 | 0.05 | 3.85 | 0.81 | 1.28 | 0.35 |
| S44pAcF/N132C | 4.32 | 0.96 | 1.44 | 0.04 | | | 4.20 | 0.84 | 1.37 | 0.37 |
| S44pAcF/I150C | 4.32 | 0.96 | 1.44 | 0.04 | | | 4.20 | 0.84 | 1.34 | 0.40 |
| N55pAcF/Q69C | 4.14 | 0.92 | 1.48 | 0.08 | | | 3.93 | 0.79 | 1.32 | 0.39 |
| N55pAcF/R119C | 4.26 | 0.78 | 2.31 | 0.15 | 0.24 | 0.07 | 3.67 | 0.80 | 1.35 | 0.41 |
| N55pAcF/N132C | 4.28 | 0.94 | 1.49 | 0.06 | | | 4.11 | 0.82 | 1.33 | 0.39 |
| N55pAcF/I150C | 4.32 | 0.69 | 3.08 | 0.25 | 0.72 | 0.06 | 3.75 | 0.79 | 1.48 | 0.40 |
| K60pAcF/P86C | 4.12 | 0.94 | 2.07 | 0.06 | | | 4.00 | 0.79 | 1.40 | 0.41 |
| K60pAcF/R119C | 4.26 | 0.91 | 1.81 | 0.09 | | | 4.04 | 0.81 | 1.34 | 0.37 |
| K60pAcF/N132C | 4.15 | 0.89 | 1.78 | 0.11 | | | 3.89 | 0.79 | 1.30 | 0.36 |
| K60pAcF/I150C | 4.09 | 0.88 | 1.76 | 0.12 | | | 3.81 | 0.77 | 1.35 | 0.37 |
| Q69pAcF/P86C | 4.20 | 0.94 | 1.52 | 0.06 | | | 4.04 | 0.81 | 1.36 | 0.37 |
| Q69pAcF/R119C | 4.20 | 0.88 | 1.64 | 0.12 | | | 3.89 | 0.79 | 1.37 | 0.38 |
| Q69pAcF/N132C | 4.20 | 0.89 | 1.47 | 0.11 | | | 3.90 | 0.80 | 1.40 | 0.38 |
| Q690pAcF/I150C | 4.20 | 0.89 | 1.88 | 0.11 | | | 3.94 | 0.80 | 0.94 | 0.35 |
| D70pAcF/R119C | 4.14 | 0.68 | 2.61 | 0.23 | 0.82 | 0.09 | 3.42 | 0.76 | 1.18 | 0.32 |
| D70pAcF/N132C | 4.08 | 0.88 | 1.12 | 0.12 | | | 3.72 | 0.78 | 1.33 | 0.36 |

† Fluorescence quantum yields are calculated from the species averaged lifetimes $\langle\tau\rangle_x = \sum_{i=0}^{n} x_i \tau^{(i)}$ , where $x_i$'s are the species fractions with reference values of quantum yield $\Phi_{FD(0)} = 0.8$ and lifetime $\langle\tau_{D(0)}\rangle_x = 4.0$ ns for Alexa488; and $\Phi_{FA} = 0.32$ and lifetime $\langle\tau_A\rangle_x = 1.17$ ns for Alexa647 (see the section "Donor and acceptor fluorescence quantum yields" in Supplementary Methods).

**Supplementary Table 2b.** Table of state-specific mean distances ($\langle R_{DAmajor}\rangle$, $\langle R_{DAminor}\rangle$) and corresponding fractions ($x_{major}$, $x_{minor}$) for the distribution fit with a 2-component model and individual species fractions ($N$=2, Supplementary Equation 36). Species with a higher fraction is assigned to as *major*. Donor only fraction ($x_{D(0)}$) is fitted individually. Fit specific chi-squared reduced values ($\chi^2_r$) are given for each fit. Average $\chi^2_r$ for this fit model is 1.0825. The results were not used for structural modeling.

| Variant | $\langle R_{DAmajor}\rangle$ [Å] | $x_{major}^{\dagger}$ | $\langle R_{DAminor}\rangle$ [Å] | $x_{minor}^{\dagger}$ | $x_{D(0)}^{*}$ | $\chi^2_r$ |
|---|---|---|---|---|---|---|
| E5pAcF/S44C | 42.2 | 0.94 | 27.9 | 0.06 | 0.074 | 1.12 |
| E5pAcF/N132C | 35.0 | 0.63 | 45.8 | 0.37 | 0.041 | 1.01 |
| R8pAcF/Q69C | 38.0 | 0.56 | 37.6 | 0.44 | 0.206 | 1.07 |
| R8pAcF/P86C | 39.5 | 0.68 | 50.1 | 0.32 | 0.178 | 1.06 |
| R8pAcF/R119C | 44.1 | 0.65 | 28.5 | 0.36 | 0.082 | 1.03 |
| R8pAcF/N132C | 17.9 | 0.84 | 40.4 | 0.16 | 0.053 | 1.02 |
| K19pAcF/Q69C | 38.9 | 0.62 | 38.9 | 0.38 | 0.513 | 1.07 |
| K19pAcF/P86C | 48.2 | 0.62 | 56.8 | 0.39 | 0.068 | 1.02 |
| K19pAcF/R119C | 49.1 | 0.55 | 49.1 | 0.45 | 0.425 | 1.05 |
| K19pAcF/N132C | 41.7 | 0.71 | 53.3 | 0.29 | 0.336 | 1.10 |
| E22pAcF/D127C | 28.1 | 0.65 | 45.2 | 0.35 | 0.581 | 0.99 |
| S36pAcF/P86C | 49.8 | 0.62 | 36.1 | 0.38 | 0.029 | 1.03 |
| S36pAcF/N132C | 38.6 | 0.54 | 50.7 | 0.46 | 0.031 | 0.96 |
| S44pAcF/Q69C | 20.9 | 0.78 | 36.9 | 0.22 | 0.068 | 1.05 |
| S44pAcF/P86C | 54.1 | 0.62 | 39.9 | 0.39 | 0.082 | 1.05 |
| S44pAcF/R119C | 56.9 | 0.68 | 41.9 | 0.32 | 0.215 | 1.12 |
| S44pAcF/D127C | 57.0 | 0.83 | 39.9 | 0.17 | 0.101 | 1.03 |
| S44pAcF/N132C | 63.8 | 0.55 | 43.9 | 0.45 | 0.357 | 1.14 |
| S44pAcF/I150C | 55.8 | 0.73 | 41.9 | 0.27 | 0.027 | 1.09 |
| N55pAcF/Q69C | 37.3 | 0.79 | 39.3 | 0.21 | 0.231 | 1.16 |
| N55pAcF/R119C | 64.8 | 0.63 | 51.3 | 0.37 | 0.196 | 1.09 |
| N55pAcF/N132C | 53.3 | 0.68 | 40.2 | 0.32 | 0.042 | 1.04 |
| N55pAcF/I150C | 49.6 | 0.97 | 32.9 | 0.04 | 0.723 | 1.13 |
| K60pAcF/P86C | 53.2 | 0.59 | 41.7 | 0.41 | 0.338 | 1.11 |
| K60pAcF/R119C | 54.5 | 0.61 | 42.2 | 0.39 | 0.263 | 1.12 |
| K60pAcF/N132C | 48.7 | 0.52 | 38.2 | 0.48 | 0.164 | 1.04 |
| K60pAcF/I150C | 39.8 | 0.74 | 51.7 | 0.26 | 0.196 | 1.05 |
| Q69pAcF/P86C | 38.6 | 0.87 | 58.7 | 0.14 | 0.396 | 1.09 |
| Q69pAcF/R119C | 40.5 | 0.87 | 52.8 | 0.13 | 0.306 | 1.10 |
| Q69pAcF/N132C | 37.4 | 0.55 | 47.2 | 0.45 | 0.236 | 1.10 |
| Q690pAcF/I150C | 41.5 | 0.64 | 53.8 | 0.37 | 0.400 | 1.09 |
| D70pAcF/R119C | 34.1 | 0.56 | 43.1 | 0.44 | 0.115 | 1.04 |
| D70pAcF/N132C | 34.8 | 0.68 | 45.3 | 0.32 | 0.142 | 1.07 |

. $^{\dagger}$ Values for the FRET populations are normalized such that $x_{minor} + x_{major} = 1$. *Donor decay was fixed and $x_{D(0)}$ represents the fraction of donor only from the total.

**Supplementary Table 2c.** Table of state-specific mean distances ($\langle R_{DAmajor}\rangle$, $\langle R_{DAminor}\rangle$) and corresponding fractions ($x_{major}$, $x_{minor}$) for the distribution fit with a 2-component model sharing global species fractions for the states ($N$=2, Supplementary Equation 36), $x_{minor}$ = 0.466, $x_{major}$=0.534. Species with higher fraction is assigned as *major*. Donor only fraction ($x_{D(0)}$) is fitted individually. Fit specific chi-squared reduced values ($\chi^2_r$) are given for each fit. Average $\chi^2_r$ for this fitting model is 1.0985. The results were not used for structural modeling.

| **Variant** | $\langle R_{DAminor}\rangle$ [Å] | $\langle R_{DAmajor}\rangle$ [Å] | $x_{D(0)}$* | $\chi^2_r$ |
|---|---|---|---|---|
| E5pAcF/S44C | 40.3 | 43.4 | 0.08 | 1.16 |
| E5pAcF/N132C | 44.4 | 32.8 | 0.04 | 1.05 |
| R8pAcF/Q69C | 37.8 | 37.8 | 0.21 | 1.08 |
| R8pAcF/P86C | 35.7 | 47.0 | 0.17 | 1.09 |
| R8pAcF/R119C | 37.3 | 45.8 | 0.10 | 1.07 |
| R8pAcF/N132C | 28.2 | 41.4 | 0.17 | 0.97 |
| K19pAcF/Q69C | 39.1 | 39.1 | 0.05 | 1.05 |
| K19pAcF/P86C | 46.2 | 55.5 | 0.06 | 1.00 |
| K19pAcF/R119C | 49.1 | 49.1 | 0.04 | 1.07 |
| K19pAcF/N132C | 38.2 | 49.2 | 0.03 | 1.11 |
| E22pAcF/D127C | 29.7 | 43.4 | 0.07 | 1.03 |
| S36pAcF/P86C | 39.3 | 51.1 | 0.03 | 1.10 |
| S36pAcF/N132C | 36.8 | 49.6 | 0.03 | 1.00 |
| S44pAcF/Q69C | 29.0 | 38.6 | 0.18 | 1.38 |
| S44pAcF/P86C | 42.8 | 55.6 | 0.08 | 1.06 |
| S44pAcF/R119C | 45.9 | 59.8 | 0.20 | 1.15 |
| S44pAcF/D127C | 49.4 | 61.7 | 0.07 | 1.11 |
| S44pAcF/N132C | 43.4 | 61.7 | 0.42 | 1.14 |
| S44pAcF/I150C | 46.7 | 58.6 | 0.01 | 1.13 |
| N55pAcF/Q69C | 37.2 | 37.2 | 0.23 | 1.17 |
| N55pAcF/R119C | 52.7 | 67.7 | 0.17 | 1.13 |
| N55pAcF/N132C | 43.7 | 55.2 | 0.04 | 1.10 |
| N55pAcF/I150C | 49.5 | 49.5 | 0.73 | 1.13 |
| K60pAcF/P86C | 41.2 | 53.9 | 0.32 | 1.17 |
| K60pAcF/R119C | 55.6 | 44.6 | 0.05 | 1.14 |
| K60pAcF/N132C | 49.2 | 39.1 | 0.17 | 1.06 |
| K60pAcF/I150C | 47.2 | 36.7 | 0.20 | 1.04 |
| Q69pAcF/P86C | 34.4 | 42.8 | 0.42 | 1.11 |
| Q69pAcF/R119C | 45.8 | 37.0 | 0.30 | 1.12 |
| Q69pAcF/N132C | 47.0 | 37.4 | 0.23 | 1.12 |
| Q690pAcF/I150C | 51.8 | 39.8 | 0.39 | 1.09 |
| D70pAcF/R119C | 31.7 | 42.1 | 0.11 | 1.04 |
| D70pAcF/N132C | 31.1 | 42.6 | 0.14 | 1.14 |

† Values for the FRET populations are normalized such that $x_{major}+x_{minor}$ =1. *Donor decay was fixed and $x_{D(0)}$ represents the fraction of donor only from the total.

**Supplementary Table 2d.** Table of determined state-specific mean distances $\langle R_{DA}^{(1)}\rangle$ and total absolute distance uncertainties $\Delta R_{DA,tot-,+}^{(1)}$ for the state $C_1$ obtained by the distribution fit with a 3-component model (*N*=3, Supplementary Equation (36)) with globally shared species fractions $x_1$ = 0.44, $x_2$ = 0.38 and $x_3$ = 0.18 and free donor-only fraction ($x_{D(0)}$). Minimal $\chi^2_{r,global}$ for this fit model is 1.0736. The total distance uncertainties $\Delta R_{DA,tot-}^{(1)}$ and $\Delta R_{DA,tot+}^{(1)}$ are calculated according to Equation 5 (main text) using the contributions of individual uncertainties listed in Supplementary Table 2g. The distances were used for FPS in Fig. 5 b,c. We present the weighted residuals (w.res.) of the conformation $C_1$ against the model structure (PDB-ID 172L). $C_\alpha$-$C_\alpha$ represents the distances between the $C_\alpha$ atoms of the labeled residues in the model structure, and $\langle R_{DA}\rangle$ represents the corresponding average inter-dye distances. The donor fluorophore was attached to the unnatural amino acid p-acetyl-L-phenylalanine (pAcF), the acceptor fluorophore to the Cysteine residue (C). The results were used for structural modeling in Figure 5.

| Variant | $\langle R_{DA}^{(1)}\rangle$ [Å] | $\Delta R_{DA,tot-}^{(1)}$ [Å] | $\Delta R_{DA,tot+}^{(1)}$ [Å] | $C_\alpha$-$C_\alpha$ X-ray* [Å] | $\langle R_{DA}\rangle$ X-ray [Å] | *w.res.* *** [Å/Å] | $x_{D(0)}$* | $\chi^2_r$ | Class |
|---|---|---|---|---|---|---|---|---|---|
| E5pAcF/S44C | 42.3 | 4.4 | 4.4 | 27.3 | 41.8 | -0.13 | 0.07 | 1.09 | $R_1 = R_2$, $R_3$ var. |
| E5pAcF/N132C | 34.7 | 5.4 | 5.4 | 25.7 | 42.8 | 1.62 | 0.04 | 1.01 | $R_1 = R_3$, $R_2$ var. |
| R8pAcF/Q69C | 37.8 | 3.9 | 3.9 | 15.1 | 34.7 | -0.80 | 0.21 | 1.08 | $R_1 = R_2 = R_3$ |
| R8pAcF/P86C | 47.6 | 5.4 | 5.3 | 26.8 | 46.1 | -0.28 | 0.16 | 1.08 | $R_2 = R_3$, $R_1$ var. |
| R8pAcF/R119C | 45.9 | 6.0 | 6.0 | 28.2 | 47.1 | 0.23 | 0.09 | 1.04 | $R_1 > R_2 > R_3$ |
| R8pAcF/N132C | 42.4 | 5.7 | 5.7 | 26.0 | 42.9 | 0.16 | 0.04 | 0.97 | $R_1 > R_3 > R_2$ |
| K19pAcF/Q69C | 39.1 | 4.0 | 4.0 | 20.2 | 37.7 | -0.35 | 0.53 | 1.05 | $R_1 = R_2 = R_3$ |
| K19pAcF/P86C | 54.2 | 5.5 | 5.5 | 35.9 | 52.4 | -0.33 | 0.04 | 1.00 | $R_1 > R_2 > R_3$ |
| K19pAcF/R119C | 56.4 | 6.2 | 6.2 | 37.5 | 51.4 | -0.81 | 0.25 | 1.07 | $R_1 = R_2 = R_3$ |
| K19pAcF/N132C | 50.4 | 7.0 | 7.0 | 35.3 | 46.5 | -0.58 | 0.33 | 1.11 | $R_2 = R_3$, $R_1$ var. |
| E22pAcF/D127C | 41.5 | 6.7 | 6.7 | 37.3 | 44.7 | 0.48 | 0.53 | 1.01 | $R_3 > R_1 > R_2$ |
| S36pAcF/P86C | 51.3 | 5.2 | 5.2 | 37.4 | 47.0 | -0.83 | 0.03 | 1.06 | $R_1 > R_2 > R_3$ |
| S36pAcF/N132C | 50.9 | 6.6 | 6.6 | 41.0 | 50.6 | -0.04 | 0.03 | 0.96 | $R_2 = R_3$, $R_1$ var. |
| S44pAcF/Q69C | 29.8 | 4.7 | 4.7 | 18.4 | 27.3 | -0.72 | 0.13 | 1.14 | $R_1 = R_2$, $R_3$ var. |
| S44pAcF/P86C | 55.8 | 6.0 | 6.0 | 39.3 | 51.5 | -0.72 | 0.07 | 1.05 | $R_1 = R_3$, $R_2$ var. |
| S44pAcF/R119C | 59.7 | 4.9 | 4.9 | 44.2 | 57.0 | -0.45 | 0.21 | 1.13 | $R_1 > R_2 > R_3$ |
| S44pAcF/D127C | 58.4 | 6.8 | 6.8 | 47.6 | 60.3 | 0.14 | 0.10 | 1.01 | $R_1 > R_2 > R_3$ |
| S44pAcF/N132C | 64.8 | 7.7 | 7.7 | 45.2 | 57.6 | -0.94 | 0.40 | 1.14 | $R_1 > R_2 > R_3$ |
| S44pAcF/I150C | 58.2 | 5.9 | 5.8 | 38.9 | 58.2 | 0.00 | 0.03 | 1.10 | $R_1 > R_2 > R_3$ |
| N55pAcF/Q69C | 37.1 | 4.4 | 4.4 | 20.9 | 34.5 | -0.59 | 0.23 | 1.17 | $R_1 = R_2 = R_3$ |
| N55pAcF/R119C | 68.4 | 5.6 | 5.6 | 46.5 | 62.5 | -1.09 | 0.18 | 1.13 | $R_1 > R_2 > R_3$ |
| N55pAcF/N132C | 55.2 | 5.5 | 5.5 | 46.2 | 60.6 | 1.05 | 0.04 | 1.08 | $R_1 > R_2 > R_3$ |
| N55pAcF/I150C | 60.8 | 6.8 | 6.8 | 38.7 | 55.7 | -0.75 | 0.48 | 1.13 | $R_1 = R_2$, $R_3$ var. |

| | | | | | | | | | |
|---|---|---|---|---|---|---|---|---|---|
| K60pAcF/P86C | 54.0 | 6.0 | 6.0 | 32.7 | 47.3 | -1.09 | 0.30 | 1.15 | $R_1 > R_2 > R_3$ |
| K60pAcF/R119C | 47.4 | 5.9 | 5.9 | 36.4 | 51.1 | 0.50 | 0.06 | 1.09 | $R_2 > R_1 > R_3$ |
| K60pAcF/N132C | 37.7 | 4.4 | 4.4 | 35.9 | 49.1 | 2.54 | 0.17 | 1.06 | $R_2 = R_3$, $R_1$ var. |
| K60pAcF/I150C | 37.8 | 4.3 | 4.3 | 27.1 | 40.3 | 0.59 | 0.20 | 1.03 | $R_1 = R_3$, $R_2$ var. |
| Q69pAcF/P86C | 38.6 | 4.3 | 4.3 | 22.0 | 34-0 | -1.15 | 0.42 | 1.11 | $R_1 = R_2$, $R_3$ var. |
| Q69pAcF/R119C | 39.9 | 4.7 | 4.7 | 27.9 | 41.9 | 0.45 | 0.30 | 1.10 | $R_1 = R_2$, $R_3$ var. |
| Q69pAcF/N132C | 37.3 | 4.5 | 4.5 | 31.0 | 45.4 | 1.81 | 0.24 | 1.12 | $R_1 = R_3$, $R_2$ var. |
| Q690pAcF/I150C | 50.8 | 5.9 | 5.9 | 24.7 | 47.4 | -0.58 | 0.36 | 1.09 | $R_1 = R_3$, $R_2$ var. |
| D70pAcF/R119C | 43.1 | 4.6 | 4.6 | 25.8 | 36.8 | -1.36 | 0.12 | 1.04 | $R_2 = R_3$, $R_1$ var. |
| D70pAcF/N132C | 38.8 | 4.8 | 4.8 | 28.2 | 38.6 | 0.00 | 0.13 | 1.07 | $R_3 > R_1 > R_2$ |

**Supplementary Table 2e.** Table of determined state-specific mean distances $\langle R_{DA}^{(2)} \rangle$ and total absolute distance uncertainties $\Delta R_{DA,tot-,+}^{(2)}$ for the state $C_2$ obtained by the distribution fit with a 3-component model ($N$=3, Supplementary Equation (36)) with globally shared species fractions $x_1$ = 0.44, $x_2$ = 0.38 and $x_3$ = 0.18 and free donor-only fraction ($x_{D(0)}$). Minimal $\chi^2_{r,global}$ for this fit model is 1.0736. The total distance uncertainties $\Delta R_{DA,tot-}^{(2)}$ and $\Delta R_{DA,tot+}^{(2)}$ are calculated according to Equation 5 (main text) using the contributions of individual uncertainties listed in Supplementary Table 2g. The distances were used for FPS in Fig. 5 b,c. We present the weighted residuals (w.res.) of the conformation $C_2$ against the model structure (PDB-ID 148L). $C_\alpha$-$C_\alpha$ represents the distances between the $C_\alpha$ atoms of the labeled residues in the model structure, and $\langle R_{DA} \rangle$ represents the corresponding average inter-dye distances. The results were used for structural modeling in Figure 5.

| Variant | $\langle R_{DA}^{(2)} \rangle$ [Å] | $\Delta R_{DA,tot-}^{(2)}$ [Å] | $\Delta R_{DA,tot+}^{(2)}$ [Å] | $C_\alpha$-$C_\alpha$ X-ray* [Å] | $\langle R_{DA} \rangle$ X-ray [Å] | *w.res.**** [Å/Å] | $x_{D(0)}$* | $\chi^2_r$ | Class |
|---|---|---|---|---|---|---|---|---|---|
| E5pAcF/S44C | 42.3 | 4.4 | 4.4 | 28.9 | 43.5 | 0.30 | 0.07 | 1.09 | $R_1 = R_2$, $R_3$ var. |
| E5pAcF/N132C | 45.6 | 7.2 | 7.2 | 25.7 | 42.9 | -0.37 | 0.04 | 1.01 | $R_1 = R_3$, $R_2$ var. |
| R8pAcF/Q69C | 37.8 | 3.9 | 3.9 | 14.3 | 32.0 | -1.49 | 0.21 | 1.08 | $R_1 = R_2 = R_3$ |
| R8pAcF/P86C | 38.2 | 4.4 | 4.4 | 26.6 | 43.3 | 1.16 | 0.16 | 1.08 | $R_2 = R_3$, $R_1$ var. |
| R8pAcF/R119C | 39.5 | 5.4 | 5.4 | 28.3 | 46.7 | 1.27 | 0.09 | 1.04 | $R_1 > R_2 > R_3$ |
| R8pAcF/N132C | 31.1 | 4.6 | 4.6 | 25.6 | 44.4 | 2.31 | 0.17 | 0.97 | $R_1 > R_3 > R_2$ |
| K19pAcF/Q69C | 39.1 | 4.0 | 4.0 | 20.9 | 38.7 | -0.10 | 0.53 | 1.05 | $R_1 = R_2 = R_3$ |
| K19pAcF/P86C | 47.2 | 4.9 | 4.9 | 34.4 | 50.7 | -0.33 | 0.04 | 1.00 | $R_1 > R_2 > R_3$ |
| K19pAcF/R119C | 44.7 | 5.0 | 5.0 | 33.5 | 47.2 | 0.50 | 0.25 | 1.07 | $R_1 = R_2 = R_3$ |
| K19pAcF/N132C | 39.7 | 5.5 | 5.5 | 28.4 | 42.0 | 0.42 | 0.33 | 1.11 | $R_2 = R_3$, $R_1$ var. |
| E22pAcF/D127C | 36.8 | 5.5 | 5.5 | 28.7 | 37.1 | 0.05 | 0.53 | 1.01 | $R_3 > R_1 > R_2$ |
| S36pAcF/P86C | 41.6 | 4.9 | 4.9 | 34.3 | 42.8 | 0.08 | 0.03 | 1.06 | $R_1 > R_2 > R_3$ |
| S36pAcF/N132C | 37.6 | 5.0 | 5.0 | 30.5 | 41.4 | 0.78 | 0.03 | 0.96 | $R_2 = R_3$, $R_1$ var. |
| S44pAcF/Q69C | 29.8 | 4.7 | 4.7 | 18.9 | 27.4 | -0.70 | 0.13 | 1.14 | $R_1 = R_2$, $R_3$ var. |
| S44pAcF/P86C | 45.8 | 5.2 | 5.2 | 37.5 | 49.0 | 0.62 | 0.07 | 1.05 | $R_1 = R_3$, $R_2$ var. |
| S44pAcF/R119C | 50.1 | 5.2 | 5.2 | 40.0 | 50.7 | 0.01 | 0.21 | 1.13 | $R_1 > R_2 > R_3$ |
| S44pAcF/D127C | 56.1 | 7.2 | 7.2 | 43.8 | 56.5 | 0.18 | 0.10 | 1.01 | $R_1 > R_2 > R_3$ |
| S44pAcF/N132C | 47.8 | 6.2 | 6.2 | 38.3 | 51.3 | 0.52 | 0.40 | 1.14 | $R_1 > R_2 > R_3$ |
| S44pAcF/I150C | 48.1 | 5.8 | 5.8 | 35.0 | 51.7 | 0.62 | 0.03 | 1.10 | $R_1 > R_2 > R_3$ |
| N55pAcF/Q69C | 37.1 | 4.4 | 4.4 | 21.5 | 33.5 | -0.82 | 0.23 | 1.17 | $R_1 = R_2 = R_3$ |
| N55pAcF/R119C | 56.6 | 5.2 | 5.2 | 44.8 | 59.8 | 0.56 | 0.18 | 1.13 | $R_1 > R_2 > R_3$ |
| N55pAcF/N132C | 46.8 | 4.7 | 4.7 | 42.1 | 58.3 | 2.58 | 0.04 | 1.08 | $R_1 > R_2 > R_3$ |
| N55pAcF/I150C | 47.6 | 5.4 | 5.4 | 37.1 | 52.9 | -0.75 | 0.48 | 1.13 | $R_1 = R_2$, $R_3$ var. |
| K60pAcF/P86C | 43.9 | 5.7 | 5.7 | 35.6 | 48.9 | 0.67 | 0.30 | 1.15 | $R_1 > R_2 > R_3$ |

| | | | | | | | | | |
|---|---|---|---|---|---|---|---|---|---|
| K60pAcF/R119C | 55.0 | 6.0 | 6.0 | 39.3 | 53.2 | -0.22 | 0.06 | 1.09 | $R_2 > R_1 > R_3$ |
| K60pAcF/N132C | 49.2 | 5.8 | 5.8 | 37.4 | 51.9 | 0.46 | 0.17 | 1.06 | $R_2 = R_3$, $R_1$ var. |
| K60pAcF/I150C | 48.5 | 5.6 | 5.6 | 29.9 | 43.6 | -0.90 | 0.20 | 1.03 | $R_1 = R_3$, $R_2$ var. |
| Q69pAcF/P86C | 36.7 | 3.8 | 3.8 | 23.2 | 34.8 | -0.46 | 0.42 | 1.11 | $R_1 = R_2$, $R_3$ var. |
| Q69pAcF/R119C | 40.0 | 5.1 | 5.1 | 28.9 | 41.7 | 0.28 | 0.30 | 1.10 | $R_1 = R_2$, $R_3$ var. |
| Q69pAcF/N132C | 47.8 | 5.7 | 5.7 | 30.1 | 45.7 | -0.38 | 0.24 | 1.12 | $R_1 = R_3$, $R_2$ var. |
| Q690pAcF/I150C | 42.2 | 5.0 | 5.0 | 24.7 | 47.3 | -0.58 | 0.36 | 1.09 | $R_1 = R_3$, $R_2$ var. |
| D70pAcF/R119C | 34.1 | 3.8 | 3.8 | 26.2 | 34.5 | 0.08 | 0.12 | 1.04 | $R_2 = R_3$, $R_1$ var. |
| D70pAcF/N132C | 30.4 | 3.6 | 3.6 | 26.6 | 37.5 | 1.92 | 0.13 | 1.07 | $R_3 > R_1 > R_2$ |

**Supplementary Table 2f.** Table of determined state-specific mean distances $\langle R_{DA}^{(3)} \rangle$ and total absolute distance uncertainties $\Delta R_{DA,tot-,+}^{(3)}$ for the state $C_3$ obtained by the distribution fit with a 3-component model (*N*=3, Supplementary Equation (36)) with globally shared species fractions $x_1 = 0.44$, $x_2 = 0.38$ and $x_3 = 0.18$ and free donor-only fraction ($x_{D(0)}$). Minimal $\chi^2_{r,\mathrm{global}}$ for this fit model is 1.0736. The total distance uncertainties $\Delta R_{DA,tot-}^{(3)}$ and $\Delta R_{DA,tot+}^{(3)}$ are calculated according to Equation 5 (main text) using the contributions of the individual uncertainties listed in Supplementary Table 2g. The distances were used for FPS in Fig. 5 b,c. The results were used for structural modeling in Figure 5.

| Variant | $\langle R_{DA}^{(3)} \rangle$ [Å] | $\Delta R_{DA,tot-}^{(3)}$ [Å] | $\Delta R_{DA,tot+}^{(3)}$ [Å] | $x_{D(0)}$* | $\chi^2_r$ | Class |
|---|---|---|---|---|---|---|
| E5pAcF/S44C | 25.1 | 3.6 | 3.6 | 0.07 | 1.09 | $R_1 = R_2$, $R_3$ var. |
| E5pAcF/N132C | 35.3 | 5.9 | 5.9 | 0.04 | 1.01 | $R_1 = R_3$, $R_2$ var. |
| R8pAcF/Q69C | 37.8 | 3.9 | 3.9 | 0.21 | 1.08 | $R_1 = R_2 = R_3$ |
| R8pAcF/P86C | 30.0 | 4.6 | 4.6 | 0.16 | 1.08 | $R_2 = R_3$, $R_1$ var. |
| R8pAcF/R119C | 27.9 | 3.7 | 3.7 | 0.09 | 1.04 | $R_1 > R_2 > R_3$ |
| R8pAcF/N132C | 25.2 | 4.8 | 4.8 | 0.17 | 0.97 | $R_1 > R_3 > R_2$ |
| K19pAcF/Q69C | 39.1 | 4.0 | 4.0 | 0.53 | 1.05 | $R_1 = R_2 = R_3$ |
| K19pAcF/P86C | 32.3 | 5.0 | 5.2 | 0.04 | 1.00 | $R_1 > R_2 > R_3$ |
| K19pAcF/R119C | 31.0 | 4.6 | 4.6 | 0.25 | 1.07 | $R_1 = R_2 = R_3$ |
| K19pAcF/N132C | 39.7 | 5.7 | 5.7 | 0.33 | 1.11 | $R_2 = R_3$, $R_1$ var. |
| E22pAcF/D127C | 25.4 | 4.1 | 4.1 | 0.53 | 1.01 | $R_3 > R_1 > R_2$ |
| S36pAcF/P86C | 29.2 | 4.3 | 4.3 | 0.03 | 1.06 | $R_1 > R_2 > R_3$ |
| S36pAcF/N132C | 41.6 | 5.8 | 5.8 | 0.03 | 0.96 | $R_2 = R_3$, $R_1$ var. |
| S44pAcF/Q69C | 42.8 | 6.1 | 6.1 | 0.13 | 1.14 | $R_1 = R_2$, $R_3$ var. |
| S44pAcF/P86C | 54.0 | 5.8 | 5.8 | 0.07 | 1.05 | $R_1 = R_3$, $R_2$ var. |
| S44pAcF/R119C | 38.5 | 4.4 | 4.4 | 0.21 | 1.13 | $R_1 > R_2 > R_3$ |
| S44pAcF/D127C | 41.4 | 7.1 | 7.1 | 0.10 | 1.01 | $R_1 > R_2 > R_3$ |
| S44pAcF/N132C | 38.9 | 4.7 | 4.7 | 0.40 | 1.14 | $R_1 > R_2 > R_3$ |
| S44pAcF/I150C | 33.4 | 4.4 | 4.4 | 0.03 | 1.10 | $R_1 > R_2 > R_3$ |
| N55pAcF/Q69C | 37.8 | 4.5 | 4.5 | 0.23 | 1.17 | $R_1 = R_2 = R_3$ |
| N55pAcF/R119C | 49.0 | 4.2 | 4.2 | 0.18 | 1.13 | $R_1 > R_2 > R_3$ |
| N55pAcF/N132C | 34.4 | 4.0 | 4.0 | 0.04 | 1.08 | $R_1 > R_2 > R_3$ |
| N55pAcF/I150C | 37.3 | 8.1 | 8.1 | 0.48 | 1.13 | $R_1 = R_2$, $R_3$ var. |
| K60pAcF/P86C | 29.7 | 4.5 | 4.5 | 0.30 | 1.15 | $R_1 > R_2 > R_3$ |
| K60pAcF/R119C | 34.1 | 4.7 | 4.7 | 0.06 | 1.09 | $R_2 > R_1 > R_3$ |
| K60pAcF/N132C | 45.7 | 5.6 | 5.6 | 0.17 | 1.06 | $R_2 = R_3$, $R_1$ var. |
| K60pAcF/I150C | 38.0 | 4.4 | 4.4 | 0.20 | 1.03 | $R_1 = R_3$, $R_2$ var. |
| Q69pAcF/P86C | 52.4 | 5.2 | 5.2 | 0.42 | 1.11 | $R_1 = R_2$, $R_3$ var. |
| Q69pAcF/R119C | 50.7 | 7.0 | 7.0 | 0.30 | 1.10 | $R_1 = R_2$, $R_3$ var. |
| Q69pAcF/N132C | 41.0 | 5.0 | 5.0 | 0.24 | 1.12 | $R_1 = R_3$, $R_2$ var. |
| Q690pAcF/I150C | 38.0 | 4.5 | 4.5 | 0.36 | 1.09 | $R_1 = R_3$, $R_2$ var. |
| D70pAcF/R119C | 34.1 | 3.7 | 3.7 | 0.12 | 1.04 | $R_2 = R_3$, $R_1$ var. |
| D70pAcF/N132C | 47.1 | 5.9 | 5.9 | 0.13 | 1.07 | $R_3 > R_1 > R_2$ |

**Supplementary Table 2g.** Table of uncertainty contributions of mean distances: the state-specific absolute statistical uncertainty $\langle R^{(i)}_{noise-,+}\rangle$ and the $R_0$ related relative uncertainty $\delta R_{R_0}(r(t)_{dye})$, for the distribution fit with a 3-component model ($N$=3, Supplementary Equation (36)) with globally shared species fractions $x_1$ = 0.44, $x_2$ = 0.38 and $x_3$ = 0.18*. The relative uncertainty $\delta R_{R_0}(r(t)_{dye})$, which is equal for all states, is computed as described as in the Methods Section "Simulation of interdye distances and structural modelling" in the main text following the procedures in Ref.[3] using the limiting anisotropies of the donor fluorescence $r_{3,\mathrm{D}}$ (Supplementary Table 3a), directly excited acceptor fluorescence $r_{3,\mathrm{A}}$ (Supplementary Table 3b) and FRET-sensitized acceptor fluorescence $r_{2,\mathrm{A(D)}}$ (Supplementary Table 3c) to compute the $\kappa^2$ distributions compiled in Supplementary Table 3d. The results were used for structural modeling in Figure 5.

| Variant | $\langle R^{(1)}_{noise-}\rangle$ [Å] | $\langle R^{(1)}_{noise+}\rangle$ [Å] | $\langle R^{(2)}_{noise-}\rangle$ [Å] | $\langle R^{(2)}_{noise+}\rangle$ [Å] | $\langle R^{(3)}_{noise-}\rangle$ [Å] | $\langle R^{(3)}_{noise+}\rangle$ [Å] | $\delta R_{R_0}(r(t)_{dye})$ [%] |
|---|---|---|---|---|---|---|---|
| E5pAcF/S44C | 42.3 | 42.5 | 42.1 | 42.3 | 23.1 | 28.1 | 10.3 |
| E5pAcF/N132C | 33.4 | 34.7 | 45.0 | 46.0 | 33.9 | 37.7 | 15.7 |
| R8pAcF/Q69C | 37.8 | 37.8 | 37.8 | 37.8 | 37.8 | 37.8 | 10.2 |
| R8pAcF/P86C | 46.9 | 48.2 | 37.3 | 39.1 | 29.3 | 30.7 | 10.7 |
| R8pAcF/R119C | 45.6 | 45.9 | 38.3 | 41.3 | 27.2 | 28.6 | 13.1 |
| R8pAcF/N132C | 43.0 | 42.7 | 35.4 | 33.8 | 27.1 | 26.2 | 13.6 |
| K19pAcF/Q69C | 39.1 | 39.1 | 39.1 | 39.1 | 39.1 | 39.1 | 10.3 |
| K19pAcF/P86C | 54.2 | 54.2 | 47.2 | 47.2 | 28.7 | 36.1 | 9.80 |
| K19pAcF/R119C | 56.4 | 56.4 | 44.7 | 44.7 | 31.0 | 31.0 | 10.7 |
| K19pAcF/N132C | 49.8 | 51.3 | 39.0 | 40.4 | 39.0 | 41.6 | 13.8 |
| E22pAcF/D127C | 37.1 | 45.9 | 33.6 | 40.0 | 22.9 | 27.8 | 11.6 |
| S36pAcF/P86C | 51.2 | 51.4 | 40.2 | 44.7 | 27.2 | 33.2 | 10.1 |
| S36pAcF/N132C | 50.3 | 51.4 | 36.6 | 38.4 | 38.1 | 42.8 | 13.0 |
| S44pAcF/Q69C | 29.0 | 32.4 | 29.0 | 32.4 | 40.9 | 48.7 | 10.6 |
| S44pAcF/P86C | 55.3 | 56.2 | 44.1 | 47.5 | 51.2 | 54.9 | 10.3 |
| S44pAcF/R119C | 58.6 | 59.8 | 47.5 | 53.8 | 34.7 | 40.9 | 8.21 |
| S44pAcF/D127C | 58.0 | 60.6 | 51.5 | 58.9 | 35.0 | 45.7 | 11.3 |
| S44pAcF/N132C | 63.3 | 66.3 | 45.5 | 50.8 | 38.1 | 40.5 | 11.6 |
| S44pAcF/I150C | 57.2 | 59.1 | 44.9 | 51.2 | 30.7 | 36.1 | 9.58 |
| N55pAcF/Q69C | 37.1 | 37.1 | 37.1 | 37.1 | 37.6 | 38.2 | 11.9 |
| N55pAcF/R119C | 68.0 | 69.2 | 54.6 | 59.2 | 47.6 | 50.3 | 8.16 |
| N55pAcF/N132C | 54.5 | 55.2 | 45.0 | 47.2 | 29.7 | 34.4 | 9.97 |
| N55pAcF/I150C | 60.3 | 61.3 | 47.2 | 48.1 | 30.4 | 44.2 | 10.9 |
| K60pAcF/P86C | 53.6 | 54.0 | 42.4 | 47.8 | 28.9 | 34.6 | 11.1 |
| K60pAcF/R119C | 45.6 | 50.8 | 53.6 | 55.5 | 32.0 | 37.6 | 10.9 |
| K60pAcF/N132C | 37.3 | 38.3 | 48.8 | 49.7 | 44.7 | 47.8 | 11.7 |
| K60pAcF/I150C | 37.3 | 38.2 | 47.8 | 49.5 | 37.2 | 38.8 | 11.3 |
| Q69pAcF/P86C | 37.2 | 40.7 | 35.6 | 37.5 | 57.5 | 54.7 | 10.1 |
| Q69pAcF/R119C | 39.1 | 40.5 | 38.1 | 42.4 | 48.4 | 55.5 | 11.6 |
| Q69pAcF/N132C | 36.8 | 37.8 | 47.5 | 48.2 | 38.3 | 41.5 | 11.9 |
| Q690pAcF/I150C | 50.2 | 51.5 | 41.4 | 42.9 | 37.3 | 38.7 | 11.1 |
| D70pAcF/R119C | 42.6 | 43.6 | 33.0 | 35.3 | 33.0 | 34.8 | 10.7 |
| D70pAcF/N132C | 36.9 | 40.3 | 30.2 | 31.0 | 45.7 | 49.6 | 11.6 |

* Considering a 1σ-confidence interval, the fraction $x_3$ of $\langle R^{(3)}_{DA}\rangle$ varies between 0.1 – 0.27 (see Fig. 4c). The corresponding $\langle R^{(i)}_{noise-}\rangle$ and $\langle R^{(i)}_{noise+}\rangle$ are the shortest and longest distance below the 1σ-threshold. The minimal $\chi^2_{\mathrm{r,global}}$ for this distribution fit model is 1.0736 with the species fraction $x_3 = x_{middle}$ = 0.18 with $x_1$ = 0.44 and $x_2$ = 0.38 and the state-specific mean distances $\langle R^{(i)}_{DA}\rangle$ a listed in the Supplementary Tables 2d-f.

**Supplementary Table 2h. Results of determined state-specific mean distances $\langle R_{DA}^{(i)} \rangle$, relative uncertainties $R_{DA,tot-,+}^{(i)}$ and species fractions $x_i$ for the distribution fit for functional variants of the S44/I150C FRET pair.** Globally shared parameters are highlighted in gray cells (Supplementary Equations (36-39)).

| Variant | $\langle R_{DA}^{(1)} \rangle$ [Å] | $\delta R_{DA,tot-,+}^{(1)}$ [%] | $x_1$† | $\langle R_{DA}^{(2)} \rangle$ [Å] | $\delta R_{DA,tot-,+}^{(2)}$ [%] | $x_2$† | $\langle R_{DA}^{(3)} \rangle$ [Å] | $\delta R_{DA,tot-,+}^{(3)}$ [%] | $x_3$† | $x_{D(0)}$* | $\chi^2_r$ |
|---|---|---|---|---|---|---|---|---|---|---|---|
| S44pAcF/I150C(-) | 65.1 | 8.0 | 0.25 | 51.7 | 8.0 | 0.55 | 38.8 | 3.0 | 0.20 | 0.01 | 1.06 |
| S44pAcF/I150C(+)*** | 65.1 | 12.3 | 0.25 | 51.7 | 12.3 | 0.57 | 38.8 | 3.9 | 0.18 | - | 1.52** |
| T26E/S44pAcF/I150C(-)*** | 65.1 | 4.7 | 0.37 | 51.7 | 3.0 | 0.35 | 34.9 | 9.0 | 0.28 | 0.62 | 1.21 |
| T26E/S44pAcF/I150C(+)*** | 65.1 | 1.9 | 0.20 | 51.7 | 1.0 | 0.28 | 34.9 | 13.0 | 0.52 | 0.74 | 1.08 |
| E11A/S44C/I150C(-)*** | 65.1 | 7.8 | 0.75 | 51.7 | 7.8 | 0.12 | 38.8 | 11.8 | 0.13 | - | 1.98** |
| E11A/S44C/I150C(+)*** | 65.1 | 4.9 | 0.56 | 51.7 | 4.9 | 0.27 | 38.8 | 17.4 | 0.17 | - | 2.00** |
| R137E/S44pAcF/I150C | 59.3 | 7.3 | 0.52 | 49.3 | 2.6 | 0.37 | 36.2 | 9.1 | 0.11 | 0.24 | 1.07 |

† Values for the FRET populations are normalized such that $x_1+x_2+x_3=1$. *Donor decay was fixed and $x_{D(0)}$ represents the fraction of donor only from the total. ** Data from single molecule experiments shows higher $\chi^2_r$ when compared to eTCSPC, due to low photon statistics. ***Sub-ensemble fit from burst analysis. For E11A/S44C/I150C, it was not possible to measure in eTCSPC due to high donor-only (double Cys variant).

## Supplementary Table 3

**Supplementary Table 3a. Donor anisotropies.** Analysis of time-resolved donor fluorescence anisotropies $r_D(t)$* for donor only labeled samples obtained by ensemble time-resolved fluorescence decays as described in [3]. The table lists the rotation correlation times ($\rho_{i,D}$) and corresponding fractions ($r_{i,D}$).

| Samples | $r_{1,D}$ | $\rho_{1,D}$ [ns] | $r_{2,D}$ | $\rho_{2,D}$ [ns] | $r_{3,D}$ | $\rho_{3,D}$ [ns] |
|---|---|---|---|---|---|---|
| 5/44-(D(0)) | 0.049 | 0.18 | 0.080 | 1.92 | 0.246 | 12.30 |
| 5/132-D(0)) | 0.036 | 0.15 | 0.041 | 1.12 | 0.298 | 8.58 |
| 8/69-(D(0)) | 0.082 | 0.17 | 0.063 | 1.47 | 0.230 | 9.76 |
| 8/86-(D(0)) | 0.078 | 0.18 | 0.066 | 1.24 | 0.231 | 9.09 |
| 8/119-(D(0)) | 0.04 | 0.11 | 0.042 | 1.08 | 0.293 | 8.15 |
| 8/132-(D(0)) | 0.049 | 0.10 | 0.047 | 0.97 | 0.279 | 7.85 |
| 19/69-(D(0)) | 0.122 | 0.26 | 0.116 | 1.84 | 0.137 | 8.47 |
| 19/86-(D(0)) | 0.122 | 0.26 | 0.116 | 1.84 | 0.137 | 8.47 |
| 19/119-(D(0)) | 0.122 | 0.26 | 0.116 | 1.84 | 0.137 | 8.47 |
| 19/132-(D(0)) | 0.082 | 0.16 | 0.057 | 1.15 | 0.237 | 10.39 |
| 22/127-(D(0)) | 0.054 | 0.14 | 0.120 | 1.78 | 0.201 | 16.32 |
| 36/86-(D(0)) | 0.095 | 0.17 | 0.089 | 1.44 | 0.192 | 12.00 |
| 36/132-(D(0)) | 0.078 | 0.13 | 0.074 | 0.91 | 0.223 | 8.12 |
| 44/69-(D(0)) | 0.093 | 0.15 | 0.089 | 1.17 | 0.193 | 9.32 |
| 44/86-(D(0)) | 0.113 | 0.19 | 0.067 | 1.26 | 0.195 | 8.60 |
| 44/119-(D(0)) | 0.113 | 0.19 | 0.067 | 1.26 | 0.195 | 8.60 |
| 44-127-(D(0)) | 0.093 | 0.15 | 0.089 | 1.17 | 0.193 | 9.32 |
| 44/132-(D(0)) | 0.113 | 0.19 | 0.067 | 1.26 | 0.195 | 8.60 |
| 44/150-(D(0)) | 0.113 | 0.19 | 0.067 | 1.26 | 0.195 | 8.60 |
| 55/69-(D(0)) | 0.268 | 0.10 | 0.107 | 0.73 | 0.071 | 7.61 |
| 55/119-(D(0)) | 0.134 | 0.17 | 0.084 | 1.27 | 0.157 | 10.89 |
| 55/132-(D(0)) | 0.245 | 0.05 | 0.130 | 0.58 | 0.120 | 6.93 |
| 55/150-(D(0)) | 0.144 | 0.20 | 0.082 | 1.22 | 0.150 | 9.52 |
| 60/86-(D(0)) | 0.096 | 0.18 | 0.081 | 1.24 | 0.198 | 8.24 |
| 60/119-(D(0)) | 0.077 | 0.16 | 0.067 | 1.17 | 0.231 | 9.00 |
| 60/132-(D(0)) | 0.096 | 0.18 | 0.081 | 1.24 | 0.198 | 8.24 |
| 60/150-(D(0)) | 0.096 | 0.18 | 0.081 | 1.24 | 0.198 | 8.24 |
| 69/86-(D(0)) | 0.115 | 0.18 | 0.092 | 1.07 | 0.167 | 8.26 |
| 69/119-(D(0)) | 0.115 | 0.18 | 0.092 | 1.07 | 0.167 | 8.26 |
| 69/132-(D(0)) | 0.115 | 0.18 | 0.092 | 1.07 | 0.167 | 8.26 |
| 69/150-(D(0)) | 0.115 | 0.18 | 0.092 | 1.07 | 0.167 | 8.26 |
| 70/119-(D(0)) | 0.108 | 0.23 | 0.094 | 1.38 | 0.173 | 8.42 |
| 70/132-(D(0)) | 0.108 | 0.23 | 0.094 | 1.38 | 0.173 | 8.42 |

* The fluorescence anisotropy decay $r_D(t)$ can be described as a sum of three exponentials:
$r_D(t) = r_{1,D} \exp(-t/\rho_{1,D}) + r_{2,D} \exp(-t/\rho_{2,D}) + r_{3,D} \exp(-t/\rho_{3,D})$ with anisotropy fractions $r_{1,D} + r_{2,D} + r_{3,D} \leq r_0$.
For Alexa488-hydroxylamine the fundamental anisotropy $r_{0,D}$ is 0.375.

**Supplementary Table 3b. Acceptor anisotropies (direct excitation).** Analysis of time-resolved fluorescence anisotropies $r_A(t)$* for direct acceptor excitation of double labeled samples obtained by ensemble time-resolved fluorescence decays as described in [3]. The table lists rotation correlation times ($\rho_{i,A}$) and corresponding fractions ($r_{i,A}$).

| **Samples** | $r_{1,A}$ | $\rho_{1,A}$ **[ns]** | $r_{2,A}$ | $\rho_{2,A}$ **[ns]** | $r_{3,A}$ | $\rho_{3,A}$ **[ns]** |
|---|---|---|---|---|---|---|
| 5/44-(DA) | 0.081 | 0.03 | 0.144 | 0.70 | 0.165 | 10.68 |
| 5/132-DA) | 0.030 | 0.14 | 0.119 | 0.84 | 0.241 | 12.11 |
| 8/69-(DA) | 0.060 | 0.08 | 0.116 | 0.57 | 0.214 | 13.96 |
| 8/86-(DA) | 0.051 | 0.05 | 0.108 | 0.73 | 0.231 | 12.53 |
| 8/119-(DA) | 0.035 | 0.08 | 0.134 | 0.66 | 0.221 | 13.12 |
| 8/132-(DA) | 0.059 | 0.10 | 0.152 | 0.79 | 0.178 | 11.26 |
| 19/69-(DA) | 0.066 | 0.04 | 0.155 | 0.71 | 0.169 | 12.58 |
| 19/86-(DA) | 0.036 | 0.17 | 0.141 | 0.87 | 0.214 | 11.38 |
| 19/119-(DA) | 0.042 | 0.09 | 0.142 | 0.79 | 0.206 | 10.73 |
| 19/132-(DA) | 0.061 | 0.09 | 0.173 | 0.78 | 0.156 | 19.93 |
| 22/127-(DA) | 0.062 | 0.05 | 0.151 | 0.91 | 0.177 | 14.09 |
| 36/86-(DA) | 0.042 | 0.12 | 0.131 | 0.80 | 0.217 | 11.18 |
| 36/132-(DA) | 0.030 | 0.03 | 0.148 | 0.80 | 0.212 | 17.19 |
| 44/69-(DA) | 0.094 | 0.02 | 0.151 | 0.99 | 0.145 | 14.80 |
| 44/86-(DA) | 0.039 | 0.16 | 0.142 | 0.90 | 0.209 | 14.11 |
| 44/119-(DA) | 0.030 | 0.17 | 0.133 | 0.82 | 0.227 | 11.47 |
| 44-127-(DA) | 0.105 | 0.35 | 0.099 | 1.72 | 0.186 | 19.97 |
| 44/132-(DA) | 0.046 | 0.06 | 0.073 | 0.73 | 0.271 | 10.13 |
| 44/150-(DA) | 0.031 | 0.14 | 0.096 | 0.82 | 0.263 | 10.36 |
| 55/69-(DA) | 0.036 | 0.07 | 0.096 | 0.71 | 0.257 | 11.24 |
| 55/119-(DA) | 0.030 | 0.09 | 0.072 | 0.74 | 0.288 | 10.84 |
| 55/132-(DA) | 0.031 | 0.07 | 0.107 | 0.63 | 0.252 | 11.14 |
| 55/150-(DA) | 0.037 | 0.16 | 0.078 | 0.96 | 0.275 | 10.37 |
| 60/86-(DA) | 0.026 | 0.09 | 0.074 | 0.76 | 0.290 | 10.10 |
| 60/119-(DA) | 0.067 | 0.12 | 0.056 | 0.67 | 0.267 | 10.45 |
| 60/132-(DA) | 0.100 | 0.12 | 0.075 | 0.78 | 0.215 | 11.38 |
| 60/150-(DA) | 0.045 | 0.02 | 0.067 | 0.53 | 0.278 | 8.61 |
| 69/86-(DA) | 0.110 | 0.23 | 0.063 | 2.86 | 0.216 | 11.06 |
| 69/119-(DA) | 0.054 | 0.07 | 0.069 | 0.75 | 0.267 | 9.80 |
| 69/132-(DA) | 0.064 | 0.21 | 0.147 | 1.05 | 0.179 | 13.87 |
| 69/150-(DA) | 0.157 | 0.70 | 0.233 | 24.87 | | |
| 70/119-(DA) | 0.039 | 0.10 | 0.087 | 0.70 | 0.264 | 11.08 |
| 70/132-(DA) | 0.054 | 0.14 | 0.080 | 0.69 | 0.256 | 8.66 |

* The fluorescence anisotropy decay $r_A(t)$ can be described as a sum of three exponentials:
$r_A(t) = r_{1,A} \exp(-t/\rho_{1,A}) + r_{2,A} \exp(-t/\rho_{2,A}) + r_{3,A} \exp(-t/\rho_{3,A})$ with anisotropy fractions $r_{1,A} + r_{2,A} + r_{3,A} \leq r_0$.
For Alexa647-maleimide $r_{0,A}$ is 0.39.

**Supplementary Table 3c. Acceptor anisotropies (FRET-sensitized).** Analysis of time-resolved fluorescence anisotropies $r_{A(D)}(t)$* for FRET-sensitized emission of acceptor of double labeled samples obtained by ensemble time-resolved fluorescence decays as described in [3] except for [1] and [2]. The table lists the rotation correlation times ($\rho_{i,A(D)}$) and corresponding fractions ($r_{i,A(D)}$).

| Samples | $r_{1,A(D)}$ | $\rho_{1,A(D)}$ [ns] | $r_{2,A(D)}$ | $\rho_{2,A(D)}$ [ns] | $\Sigma\ r_{i,A(D)}$ |
|---|---|---|---|---|---|
| 5/44-(DA) | 0.052 | 0.192 | 0.010 | 8.126 | 0.062 |
| 5/132-DA) | 0.087 | 0.320 | 0.026 | 10.008 | 0.113 |
| 8/69-(DA) | 0.025 | 0.509 | 0.041 | ∞ | 0.066 |
| 8/86-(DA) | 0.032 | 0.438 | 0.049 | 380 | 0.081 |
| 8/119-(DA) | 0.061 | 0.127 | 0.012 | ∞ | 0.073 |
| 8/132-(DA) | 0.091 | 0.280 | 0.020 | 4.163 | 0.111 |
| 19/69-(DA) | 0.081 | 0.398 | 0.105 | 48.063 | 0.186 |
| 19/86-(DA)[1] | 0.209 | 0.756 | 0.0561 | 19.901 | 0.265 |
| 19/119-(DA) | 0.041 | 0.512 | 0.091 | 202 | 0.132 |
| 19/132-(DA) | 0.1 | 0.373 | 0.112 | 88.561 | 0.212 |
| 22/127-(DA) | 0.044 | 0.702 | 0.018 | ∞ | 0.062 |
| 36/86-(DA) | 0.087 | 0.243 | 0.007 | 9.393 | 0.094 |
| 36/132-(DA) | 0.086 | 0.241 | 0.020 | 2.095 | 0.106 |
| 44/69-(DA) | 0.033 | 0.282 | 0.019 | 7.958 | 0.052 |
| 44/86-(DA)[2] | <0.06 | | | | |
| 44/119-(DA)[2] | <0.09 | | | | |
| 44-127-(DA) | 0.179 | 0.326 | 0.017 | 8.815 | 0.196 |
| 44/132-(DA) | 0.054 | 0.246 | 0.115 | 23.934 | 0.169 |
| 44/150-(DA) | 0.087 | 0.563 | 0.048 | 101.937 | 0.135 |
| 55/69-(DA) | 0.036 | 0.405 | 0.069 | 63.43 | 0.105 |
| 55/119-(DA) | 0.067 | 1.31 | 0.089 | 136.651 | 0.156 |
| 55/132-(DA) | 0.064 | 1.039 | 0.016 | 14.346 | 0.080 |
| 55/150-(DA) | 0.065 | 0.512 | 0.061 | 150.739 | 0.126 |
| 60/86-(DA) | 0.103 | 0.483 | 0.104 | 127.327 | 0.207 |
| 60/119-(DA) | 0.079 | 0.501 | 0.086 | 114.851 | 0.165 |
| 60/132-(DA) | 0.054 | 1.035 | 0.058 | 74.739 | 0.112 |
| 60/150-(DA) | 0.038 | 1.102 | 0.067 | 77.378 | 0.105 |
| 69/86-(DA) | 0.038 | 0.604 | 0.073 | ∞ | 0.111 |
| 69/119-(DA) | 0.045 | 0.603 | 0.059 | 84.864 | 0.104 |
| 69/132-(DA) | 0.039 | 0.294 | 0.049 | 72.456 | 0.088 |
| 69/150-(DA) | 0.049 | 0.595 | 0.048 | 210.295 | 0.097 |
| 70/119-(DA)[2] | <0.04 | 0.2416 | | | |
| 70/132-(DA)[2] | <0.04 | 0.2471 | | | |

* The fluorescence anisotropy decay $r_{A(D)}(t)$ can be described as a sum of two exponentials: $r_{A(D)}(t) = r_{1,A(D)} \exp(-t/\rho_{1,A(D)}) + r_{2,A(D)} \exp(-t/\rho_{2,A(D)})$ with anisotropy fractions $r_{1,A(D)} + r_{2,A(D)} \leq r_0$. For FRET-sensitized anisotropy decay of Alexa647-maleimide $r_{0,A(D)}$ is 0.38.

[1] eTCSPC data not available. Fluorescence anisotropy decay was fitted from sub-ensemble single-molecule MFD data of the FRET population.
[2] eTCSPC data not available. Considering variants with a very high FRET efficiency, no satisfactory anisotropy decays from sub-ensemble single-molecule MFD data were obtainable due the short donor fluorescence lifetime. Here, steady-values anisotropies were taken as upper limit from single-molecule MFD measurements.

**Supplementary Table 3d. $\kappa^2$ distributions for the 33 DA samples.** Donor positions are labeled on green and acceptor positions on red. The mean $\kappa^2$ ($\langle\kappa^2\rangle$) is shown as a solid bar in blue, and $\kappa^2$ = 2/3 is shown in red. Therefore, the assumption of $\kappa^2$ = 2/3 is justified. Nevertheless, the $\kappa^2$ distribution adds to the uncertainty on our distances, which is considered as previously described in [3].

**E5pAcF S44C**

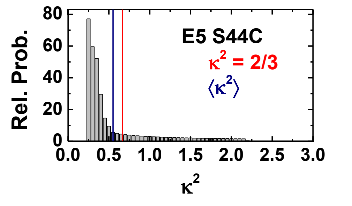


**E5pAcF N132C**

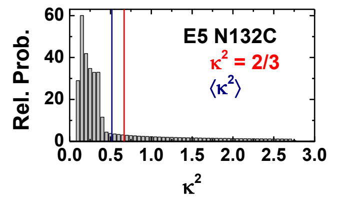


**R8pAcF N55C**

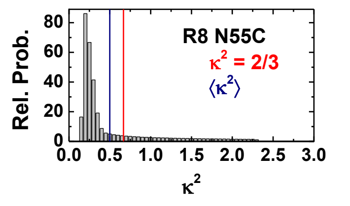


**R8pAcF Q69C**

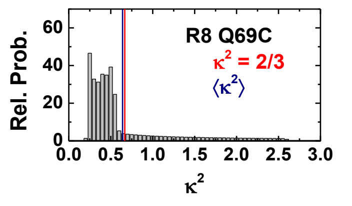


**R8pAcF P86C**

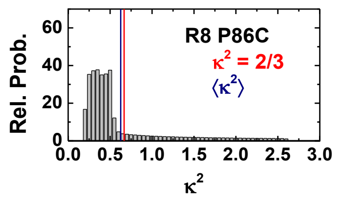


**R8pAcF R119C**

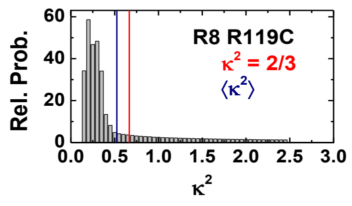


**R8pAcF N132C**

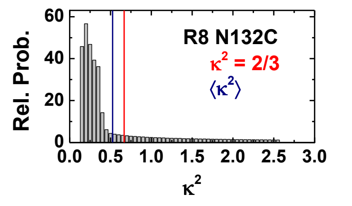


**K19pAcF Q69C**

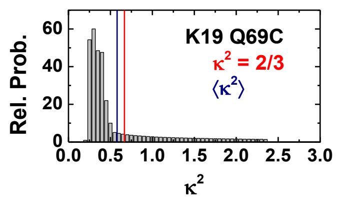


**K19pAcF P86C**

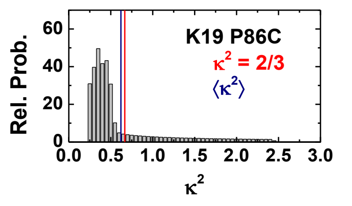


**K19pAcF R119C**

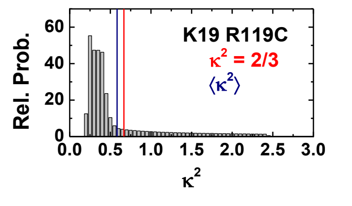


**K19pAcF N132C**

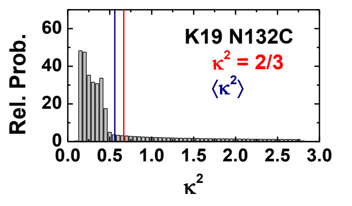


**E22pAcF P86C**

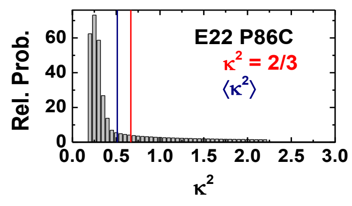


**E22pAcF D127C**

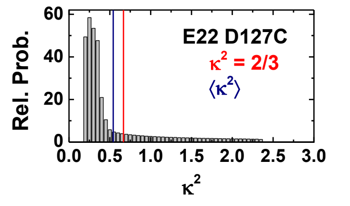


**S36pAcF P86C**

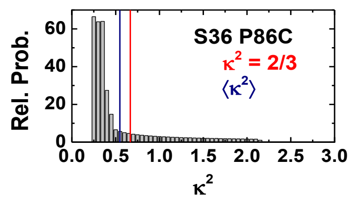


**S36pAcF N132C**

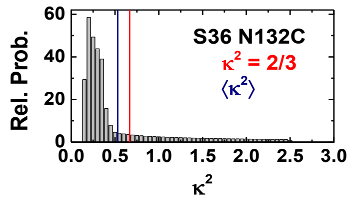


**S44pAcF Q69C**

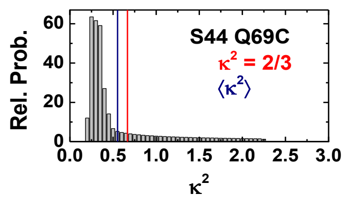


**S44pAcF P86C**

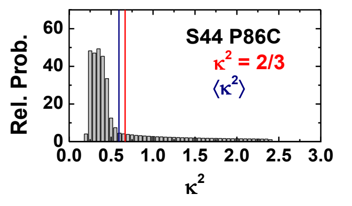


**S44pAcF R119C**

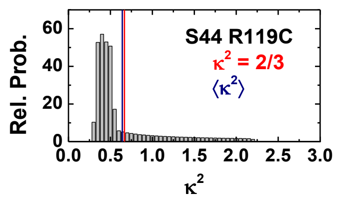


**S44pAcF D127C**

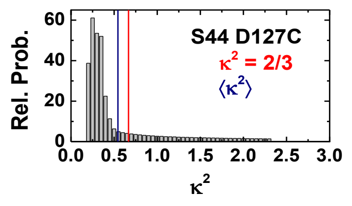


**S44pAcF N132C**

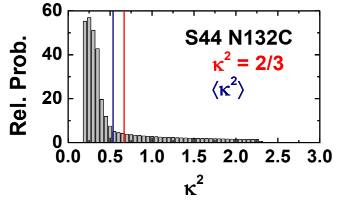

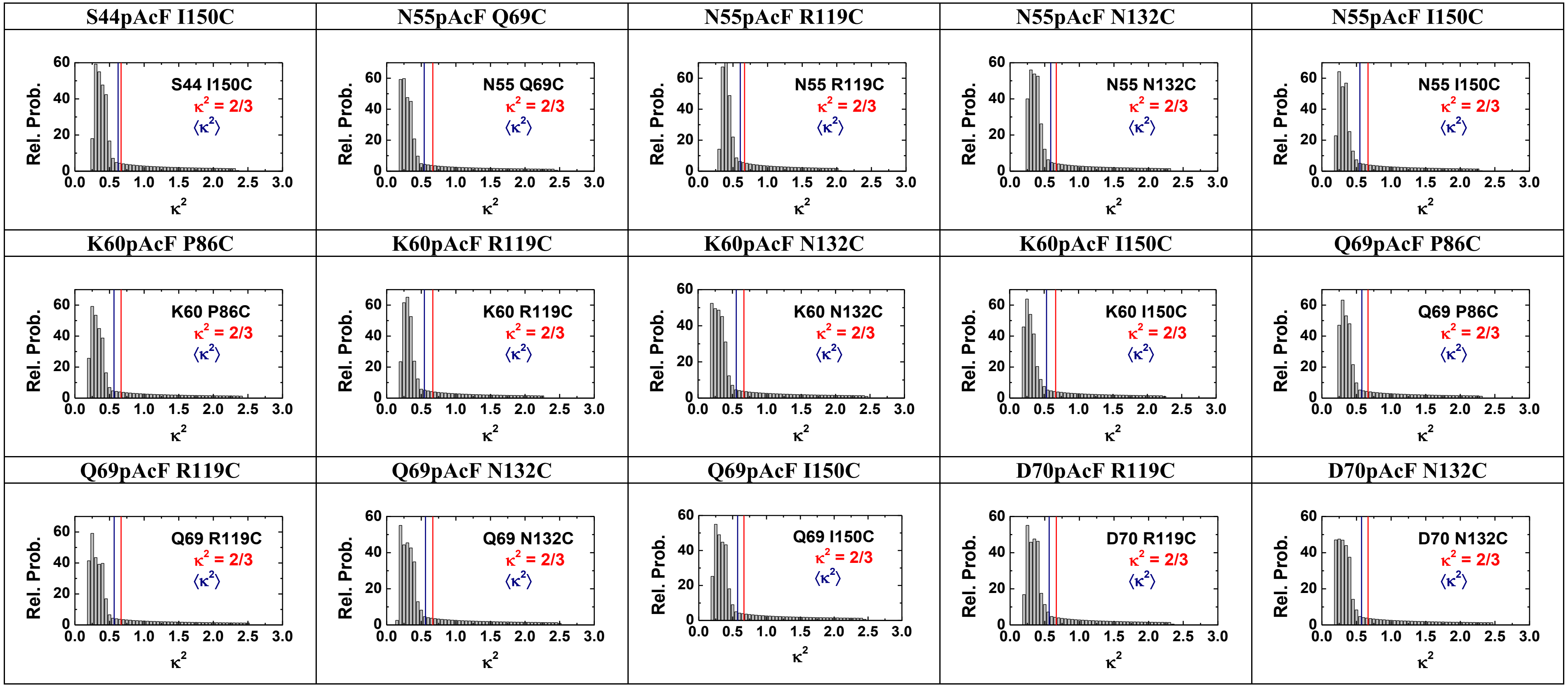
S44pAcF I150C
N55pAcF Q69C
N55pAcF R119C
N55pAcF N132C
N55pAcF I150C
K60pAcF P86C
K60pAcF R119C
K60pAcF N132C
K60pAcF I150C
Q69pAcF P86C
Q69pAcF R119C
Q69pAcF N132C
Q69pAcF I150C
D70pAcF R119C
D70pAcF N132C
S44 I150C
N55 Q69C
N55 R119C
N55 N132C
N55 I150C
K60 P86C
K60 R119C
K60 N132C
K60 I150C
Q69 P86C
Q69 R119C
Q69 N132C
Q69 I150C
D70 R119C
D70 N132C
κ² = 2/3
⟨κ²⟩
Rel. Prob.
κ²

## Supplementary Table 4

**Supplementary Table 4**. **Groups of structure models for T4L in PDB**. All 578 structural models could be grouped in three clusters: *Open* (19 structures), *ajar* (26 structures) and *closed* (535 structures).

| Cluster-name | PDB-ID |
|---|---|
| *Open* (19) | 172L, 151L, 168L, 169L, 173L, 174L, 178L, 1L97, 2HUK, 3EML, 3JR6, 3OE8, 3QAK, 3RZE, 3SB5, 4ARJ, 4IAP, 4K5Y, 4OO9. |
| *Ajar* (26) | 1JQU, 149L, 150L, 189L, 1P5C, 1P7S, 1QTH, 1SSY, 218L, 2HUM, 2QAR, 2QB0, 2RH1, 3PBL, 3SB9, 3SBA, 3SBB, 3SN6, 3UON, 3V2W, 3V2Y, 3VW7, 4DJH, 4EPI, 4EXM, 4GBR |
| *Closed* (535) | 148L, 102L, 103L, 104L, 107L, 108L, 109L, 110L, 111L, 112L, 113L, 114L, 115L, 118L, 119L, 120L, 122L, 123L, 125L, 126L, 127L, 128L, 129L, 130L, 131L, 137L, 138L, 139L, 140L, 141L, 142L, 143L, 144L, 145L, 146L, 147L, 152L, 155L, 156L, 157L, 158L, 159L, 160L, 161L, 162L, 163L, 164L, 165L, 166L, 167L, 170L, 171L, 175L, 176L, 177L, 180L, 181L, 182L, 183L, 184L, 185L, 186L, 187L, 188L, 190L, 191L, 192L, 195L, 196L, 197L, 198L, 199L, 1B6I, 1C60, 1C61, 1C62, 1C63, 1C64, 1C65, 1C66, 1C67, 1C68, 1C69, 1C6A, 1C6B, 1C6C, 1C6D, 1C6E, 1C6F, 1C6G, 1C6H, 1C6I, 1C6J, 1C6K, 1C6L, 1C6M, 1C6N, 1C6P, 1C6Q, 1C6T, 1CTW, 1CU0, 1CU2, 1CU3, 1CU5, 1CU6, 1CUP, 1CUQ, 1CV0, 1CV1, 1CV3, 1CV4, 1CV5, 1CV6, 1CVK, 1CX6, 1CX7, 1D2W, 1D2Y, 1D3F, 1D3J, 1D3M, 1D3N, 1D9W, 1DYA, 1DYB, 1DYC, 1DYD, 1DYE, 1DYF, 1DYG, 1EPY, 1G06, 1G07, 1G0G, 1G0J, 1G0K, 1G0L, 1G0M, 1G0P, 1G0Q, 1G1V, 1G1W, 1I6S, 1JTM, 1JTN, 1KNI, 1KS3, 1KW5, 1KW7, 1KY0, 1KY1, 1L00, 1L01, 1L02, 1L03, 1L04, 1L05, 1L06, 1L07, 1L08, 1L09, 1L0J, 1L0K, 1L10, 1L11, 1L12, 1L13, 1L14, 1L15, 1L16, 1L17, 1L18, 1L19, 1L20, 1L21, 1L22, 1L23, 1L24, 1L25, 1L26, 1L27, 1L28, 1L29, 1L30, 1L31, 1L32, 1L33, 1L34, 1L35, 1L36, 1L37, 1L38, 1L39, 1L40, 1L41, 1L42, 1L43, 1L44, 1L45, 1L46, 1L47, 1L48, 1L49, 1L50, 1L51, 1L52, 1L53, 1L54, 1L55, 1L56, 1L57, 1L58, 1L59, 1L60, 1L61, 1L62, 1L63, 1L64, 1L65, 1L66, 1L67, 1L68, 1L69, 1L70, 1L71, 1L72, 1L73, 1L74, 1L75, 1L76, 1L77, 1L79, 1L80, 1L81, 1L82, 1L83, 1L84, 1L85, 1L86, 1L87, 1L88, 1L89, 1L90, 1L91, 1L92, 1L93, 1L94, 1L95, 1L96, 1L98, 1L99, 1LGU, 1LGW, 1LGX, 1LI2, 1LI3, 1LI6, 1LLH, 1LPY, 1LW9, 1LWG, 1LWK, 1LYD, 1LYE, 1LYF, 1LYG, 1LYH, 1LYI, 1LYJ, 1NHB, 1OV5, 1OV7, 1OVH, 1OVJ, 1OVK, 1OWY, 1OWZ, 1OYU, 1P2L, 1P2R, 1P36, 1P37, 1P3N, 1P46, 1P56, 1P64, 1P6Y, 1PQD, 1PQI, 1PQJ, 1PQK, 1PQM, 1PQO, 1QS5, 1QS9, 1QSB, 1QSQ, 1QT3, 1QT4, 1QT5, 1QT6, 1QT7, 1QT8, 1QTB, 1QTC, 1QTD, 1QTV, 1QTZ, 1QUD, 1QUG, 1QUH, 1QUO, 1SSW, 1SWY, 1SWZ, 1SX2, 1SX7, 1T6H, 1T8A, 1T8F, 1T8G, 1T97, 1TLA, 1XEP, 1ZUR, 1ZWN, 1ZYT, 200L, 201L, 205L, 206L, 209L, 210L, 211L, 212L, 213L, 214L, 215L, 216L, 217L, 219L, 220L, 221L, 222L, 223L, 224L, 225L, 226L, 227L, 228L, 229L, 230L, 231L, 232L, 233L, 234L, 235L, 236L, 237L, 238L, 239L, 240L, 241L, 242L, 243L, 244L, 245L, 246L, 247L, 248L, 249L, 250L, 251L, 252L, 253L, 254L, 255L, 256L, 257L, 258L, 259L, 260L, 261L, 262L, 2A4T, 2B6T, 2B6W, 2B6X, 2B6Y, 2B6Z, 2B70, 2B72, 2B73, 2B74, 2B75, 2B7X, 2CUU, 2F2Q, 2F32, 2F47, 2HUL, 2IGC, 2L78, 2LC9, 2LCB, 2LZM, 2NTG, 2NTH, 2O4W, 2O79, 2O7A, 2OE4, 2OE7, 2OE9, 2OEA, 2OTY, 2OTZ, 2OU0, 2OU8, 2OU9, 2Q9D, 2Q9E, 2RAY, 2RAZ, 2RB0, 2RB1, 2RB2, 2RBN, 2RBO, 2RBP, 2RBQ, 2RBR, 2RBS, 3C7W, 3C7Y, 3C7Z, 3C80, 3C81, 3C82, 3C83, 3C8Q, 3C8R, 3C8S, 3CDO, 3CDQ, 3CDR, 3CDT, 3CDV, 3DKE, 3DMV, 3DMX, 3DMZ, 3DN0, 3DN1, 3DN2, 3DN3, 3DN4, 3DN6, 3DN8, 3DNA, 3F8V, 3F9L, 3FA0, 3FAD, 3FI5, 3G3V, 3G3W, 3G3X, 3GUI, 3GUJ, 3GUK, 3GUL, 3GUM, 3GUN, 3GUO, 3GUP, 3HH3, 3HH4, 3HH5, 3HH6, 3HT6, 3HT7, 3HT8, 3HT9, 3HTB, 3HTD, 3HTF, 3HTG, 3HU8, 3HU9, 3HUA, 3HUK, 3HUQ, 3HWL, 3K2R, 3L2X, 3L64, 3LZM, 3NY8, 3NY9, 3NYA, 3RUN, 3SB6, 3SB7, 3SB8, 4DAJ, 4DKL, 4E97, 4EJ4, 4EKP, 4EKQ, 4EKR, 4EKS, 4GRV, 4I7J, 4I7K, 4I7L, 4I7M, 4I7N, 4I7O, 4I7P, 4I7Q, 4I7R, 4I7S, 4I7T, 4LDE, 4LDL, 4LDO, 4LZM, 4PHU, 4TN3, 5LZM, 6LZM, 7LZM |

## Supplementary Table 5

**Supplementary Table 5**. **List of evaluated fit models**. The fit models are differentiated by their number of states and free parameters. The average $\chi^2_r$ and the table with the listed results is given.

| *N*-states | parameter | | Constraints | free parameters | | Table | Average $\chi^2_r$ |
|---|---|---|---|---|---|---|---|
| | local | global | | per sample | total | | |
| 2 | $\langle R_{DA}^{(1)} \rangle$, $x_{DA}^{(1)}$ $\langle R_{DA}^{(2)} \rangle$, $x_{DA}^{(2)}$ | | $x_{DA}^{(1)} + x_{DA}^{(2)} = 1$ | **3** | **101** =(33*3+ 3-1) | S2b | 1.0825 |
| 2 | $\langle R_{DA}^{(1)} \rangle$, $\langle R_{DA}^{(2)} \rangle$ | $x_{DA}^{(1)}$, $x_{DA}^{(2)}$ | $x_{DA}^{(1)} + x_{DA}^{(2)} = 1$ | **2** | **67** =(33*2+2- 1) | S2c | 1.0985 |
| 3 | $\langle R_{DA}^{(1)} \rangle$, $\langle R_{DA}^{(2)} \rangle$, $\langle R_{DA}^{(3)} \rangle$ | $x_{DA}^{(1)}$, $x_{DA}^{(2)}$, $x_{DA}^{(3)}$ | $x_{DA}^{(1)} + x_{DA}^{(2)} + x_{DA}^{(3)} = 1$ | **3** | **101** =(33*3+3- 1) | S2d-S2f | 1.0736 |

## Supplementary Table 6

**Supplementary Table 6**. **List of primers used within this work.** T4Lfor and T4Lrev were used for subcloning into the pet11a vector. Note that T4Lfor lies within the backbone of pet11a to have sufficient distance to the first mutation site (amino acid residue 5).

| Primer* | Sequence (5'->3') |
|---|---|
| **T4Lfor** | GGAATGGTGCATGCAAGGAGATGG |
| **T4Lend**** | GCC*GGATCC*TTATAGATTTTTATACGC |
| **E5Amber for** | ATGAATATATTTTAGATGTTACGTATAGAT |
| **E5Amber rev** | ATCTATACGTAACTACTAAAATATATTCAT |
| **R8Amber for** | AATATATTTGAAATGTTATAGATAGATGAACGTCTTAGA |
| **R8Amber rev** | TCTAAGACGTTCATCTATCTATAACATTTCAAATATATT |
| **E11A for** | GAAATGTTACGTATAGATGCTGGTCTTAGACTTAAAATC |
| **E11A rev** | GATTTTAAGTCTAAGACCAGCATCTATACGTAACATTTC |
| **T26E for** | GACACAGAAGGCTATTACGAGATTGGCATCGGTCATTTG |
| **T26E rev** | CAAATGACCGATGCCAATCTCGTAATAGCCTTCTGTGTC |
| **K19Amber for** | CTTAGACTTAAAATCTATTAGGACACAGAAGGCTATTAC |
| **K19Amber rev** | GTAATAGCCTTCTGTGTCCTAATAGATTTTAAGTCTAAG |
| **E22Amber for** | AAAATCTATAAAGACACATAGGGCTATTACACTATTGGC |
| **E22Amber rev** | GCCAATAGTGTAATAGCCCTAGTGTCTTTATAGATTTT |
| **S36Amber for** | GGTCATTTGCTTACAAAATAGCCATCACTTAATGCTGCT |
| **S36Amber rev** | AGCAGCATTAAGTGATGGCTATTTTGTAAGCAAATGACC |
| **S44Amber for** | TCACTTAATGCTGCTAAATAGGAATTAGATAAAGCTATT |
| **S44Amber rev** | AATAGCTTTATCTAATTCCTATTTAGCAGCATTAAGTGA |
| **S44C for** | TCACTTAATGCTGCTAAATGTGAATTAGATAAAGCTATT |
| **S44C rev** | AATAGCTTTATCTAATTCACATTTAGCAGCATTAAGTGA |
| **N55Amber for** | GCTATTGGGCGTAATACTTAGGGTGTAATTACAAAAGAT |
| **N55Amber rev** | ATCTTTTGTAATTACACCCTAAGTATTACGCCCAATAGC |
| **K60Amber for** | ACTAATGGTGTAATTACATAGGATGAGGCTGAAAAACTC |
| **K60Amber rev** | GAGTTTTTCAGCCTCATCCTATGTAATTACACCATTAGT |
| **Q69Amber for** | GCTGAAAAACTCTTTAATTAGGATGTTGATGCTGCTGTT |
| **Q69Amber rev** | AACAGCAGCATCAACATCCTAATTAAAGAGTTTTTCAGC |
| **Q69C for** | GCTGAAAAACTCTTTAATTGTGATGTTGATGCTGCTGTT |
| **Q69C rev** | AACAGCAGCATCAACATCACAATTAAAGAGTTTTTCAGC |
| **D70Amber for** | GAAAAACTCTTTAATCAGTAGGTTGATGCTGCTGTTCGC |
| **D70Amber rev** | GCGAACAGCAGCATCAACCTACTGATTAAAGAGTTTTTC |
| **P86C for** | AGAAATGCTAAATTAAAATGTGTTTATGATTCTCTTGAT |
| **P86C rev** | ATCAAGAGAATCATAAACACATTTTAATTTAGCATTTCT |
| **R119C for** | GGATTTACTAACTCTTTATGTATGCTTCAACAAAAACGC |
| **R119C rev** | GCGTTTTTGTTGAAGCATACATAAAGAGTTAGTAAATCC |
| **D127C for** | CTTCAACAAAAACGCTGGTGTGAAGCAGCAGTTAACTTA |
| **D127C rev** | TAAGTTAACTGCTGCTTCACACCAGCGTTTTTGTTGAAG |
| **N132C for** | TGGGATGAAGCAGCAGTTTGTTTAGCTAAAAGTAGATGG |
| **N132C rev** | CCATCTACTTTTAGCTAAACAAACTGCTGCTTCATCCCA |
| **R137E for** | CAATTGAATCGATTTTCA CTTACCATATTAGTTTGTGGA |
| **R137E rev** | GTTAACTTAGCTAAAAGTGAATGGTATAATCAAACACCT |
| **I150C for** | AATCGCGCAAAACGAGTCTGTACAACGTTTAGAACTGGC |
| **I150C rev** | GCCAGTTCTAAACGTTGTACAGACTCGTTTTGCGCGATT |

*The underlined nucleotides mark the mutation side.
**The italic nucleotides mark the restriction enzyme recognition site

## *Supplementary Notes*

### Supplementary Note 1
### Single-molecule and fluorescence correlation spectroscopy
### 1-1. Example data analysis of MFD experiments

The smFRET data analysis is done in accordance to previously published methods e.g. Kalinin *et al.*, Sisamakis *et al.* or Kudryavtsev *et al.* [4 5].

Briefly, the photons emitted from single molecules traversing through the confocal detection volume are selected from background photons using the inter-photon time and a threshold for the maximum inter photon time [6]. Additionally, only burst containing a minimum number of 60 photons were selected for further analysis.

After burst selection, our MFD histograms are checked for e. g. signal stability (Supplementary Figure 3a-c), photobleaching (Supplementary Figure 3d), contamination with free dye (Supplementary Figure 2e), multimolecule events (Supplementary Figure 3d, f) or aggregates (Supplementary Figure 3f). Without coating the measurement chamber either with the tensile Tween20 (Supplementary Figure 3b) or unlabeled protein (Supplementary Figure 3c), the signal of our FRET-labeled molecules is lost within the 10 min needed to start the experiment (Supplementary Figure 3a). Photobleaching of the acceptor will appear as tilting in the 1D projection of |Tg-Tr| (Supplementary Figure 3d); the photon trace of the acceptor will be shorter than for the donor and thus, shift the – in ideal case sharp and randomly around 0 distributed – plot. Fluorophores coupled to a biomolecule have high anisotropy a follow the Perrin equation (Supplementary Figure 3e), free dye has a very low anisotropy ~ 0. Thus, it be visible below the biomolecule population. The presence of multimolecule events or aggregates can (*i*) be detected during burst selection, (*ii*) large |Tg-Tr| and/or (*iii*) by a large number of photons within the burst and a long burst duration, respectively (Supplementary Figure 3f).

### 1-2. Additional SMD and fFCS

To test for possible influences of the dyes on the protein, two distinct labeling configurations (DA) and (AD) were prepared as previously described. In the sub-µs to ms range the dynamics of T4L is independent of the labeling-configuration. However, we can see some small differences in the two samples. For example, the species fractions in eTCSPC for S44pAcF/I150C-(DA) and –(AD) are not identical; although, one can clearly identify the same conformers corresponding to the states $C_1$, $C_2$, and $C_3$.

Slight differences were observed when comparing experiments for -(DA) and –(AD) at the single-molecule level. When comparing the mutant S44pAcF/I150C-(DA), shown in Figure S8a, to the –(AD) labeling scheme shown in Figure S2, we observe the following: *i*) There is more "donor-only" fraction in the -(AD) labeling scheme than in the -(DA), this is part of the variability in labeling. *ii*) There is no accumulation of a high FRET state in the –(AD) scheme.

However, in this situation the elongation toward higher FRET or state $C_3$ is slightly more pronounced. This elongation is also present in the T26E/S44pAcF/I150C-(AD) mutant (Supplementary Figure 10b). This resembles the accumulation found in the sample T26E/S44pAcF/I150C–(DA) (Figure 6g, main text). Regardless of these differences, the 2D

histograms and eTCSPC show similar states. This is clear evidence that the three conformational states are present independent of the fluorophores.

In summary, the kinetic scheme might change slightly, but not significantly given the conserved effect on the *sCCF* curves (Figure 3b, main text). The *sCCF* shows unequivocally that the transition times are present in both labeling schemes. Therefore, the specific dye-protein interactions are not responsible for the transition times between sub-µs and ms.

The major difference between the –(DA) and –(AD) is the state $C_{3t}$. This state seems to accumulate for the –(DA) configuration. However, at low pH the –(AD) shows a similar elongation towards the $C_3$ state similar as T26E/S44pAcF/I150C–(AD), also consistent with the data presented for the S44pAcF/I150C– (DA) at low pH.

Additional MFD histograms for further functional mutants are shown in Supplementary Figure 7. A summary of the ensemble or sub-ensemble fits for these mutants is shown in Supplementary Table 2h. Supplementary Figure 2 shows MFD histograms for all 33 variants used within the T4L network.

**Supplementary Note 2**
**Species Cross Correlation function of –(DA) and –(AD) labeled samples**

Theoretically, the species cross-correlation function (*sCCF*), as defined in Supplementary Equation (25), can be extended to more than two species in solution. Practically, this suffers of technical limitations. The more species one has in solution, the more photons are required to differentiate between them. Therefore, we selected two pseudo-species that represent mixtures of the states found in solution. In addition, we added a third pseudo species that takes into consideration the contribution of scatter photons [7]. In this approach, the meaning of specific amplitudes and their relationships is lost; however, *sCCF* can extract the relaxation times as kinetic signatures of conformational transitions between all possible states.

For all data presented, we generated two pseudo-species, plus the addition of the scatter-filter. Decays were generated accordingly to Supplementary Equations (30)-(31), based on sub-ensemble burst analysis and eTCSPC data. In some cases, lifetimes of the pseudo-species were adjusted by 100's of ps to properly cross the y-axis of the correlation at a predetermined time for visual comparison. This procedure does not affect the recovered relaxation times.

Considering the case of the double labeled mutants S44pAcF/I150C-(DA) and –(AD), the patterns $p_j^{(i)}$ that correspond to the normalized probability distributions for the –(DA) and –(AD) samples are shown on Supplementary Figure 9a, c. The parameters used on the decay generation are shown in the caption. From these patterns, the filters $f_j^{(i)}$ (Supplementary Equation (26)) were calculated. These are shown in panels B and D of Supplementary Figure 9. These filters are then used to compute the *sCCF* by multiplying each photon and weighting its contribution to each state as in Supplementary Equation (25). The patterns that are shown in Supplementary Figure 9 correspond to only half of the detectors. The other half shows similar patterns. The need of another set of detectors with similar patterns and decays is to increase the amount of pair correlations and to exclude detector after-pulsing related artifacts from calculations. Finally, a full correlation containing all relaxation times and the characteristic diffusion time can be extracted. The reproducibility of the methodology is observed by the overlap of the two species cross-correlations (Figure 3b, main text), even with the fact that different parameters were used on the generation of the filters. Similar

overlap is shown for the mutant S44pAcF/I150C-(DA) and –(AD) at pH 3.0 (Supplementary Figure 9e). For the functional mutants (E11A, T26E, R137E), we show the *sCCF* (Supplementary Figure 9f-h).

**Supplementary Note 3**

**Analyzing the kinetic network of conformational states in T4L**

**3-1. Detection of distinct $C_3$ species**

The eTCSPC fluorescence decay of S44pAcF/I150C-(DA) was fit with a model containing three different conformational states ($C_1$, $C_2$ and $C_3$). The total population of $C_3$ corresponds to 20 % (Supplementary Table 2h). From the single molecule MFD histograms it was clear to observe burst accumulation at the location where $C_3$ lies. To quantify the amount of bursts corresponding to this, we computed the area under the curve for the region of $10^{-2} < F_D/F_A < 0.3$ (Supplementary Figure 4e) corresponding to 564 burst of the total 10139 burst of all single molecule events (subtracting 893 bursts from molecules missing an active acceptor). Thus, the burst accumulation of this state is 5.3% of the total number of bursts. We called this population $C_{3t}$ because it is a static accumulation of the population $C_3$ observed by eTCSPC. In order to account for the missing 15 % of $C_3$, there has to be an additional population, which exchanges with $C_1$ and $C_2$ at timescales faster than the burst duration. We called this population $C_{3d}$. Therefore; the total contribution of equals to the sum of the static plus the dynamic subpopulations of $C_3$ ($C_3$ = 20 % = $C_{3t} + C_{3d}$ = 5 % + 15 %). Because in fFCS we only observe two relaxation times from µs to ms, we ignore for the time being the existence of the 5 % of $C_{3t}$, as it is not needed to discuss the connectivity between $C_1$, $C_2$ and $C_3$ at faster timescales.

**3-2. Consolidated model of T4L**

To construct the best kinetic model that describes the free enzyme in solution let us consider the experimental facts: *i*) eTCSPC resolves three different FRET states. *ii*) fFCS shows two transition times faster than 10 ms. *iii*) smFRET diagrams are better described by a unimodal distribution mixed with a small population (~ 5%) with very high FRET only for the S44pAcF/I150C-(DA) variant.

Unimodal distributions in single-molecule experiments can occur due to time-averaging. Ignoring the donor only population, the free enzyme (S44pAcF/I150C-(DA)) samples four conformational states ($C_1$, $C_2$, $C_{3d}$ and $C_{3t}$), where the $C_{3t}$ is a static population at very high FRET, and $C_1$, $C_2$, $C_{3d}$ mix at the observed times of ~4 µs and ~230 µs.

Putting aside the state $C_{3t}$, the simplest model of conformational transitions that one can build from experimental observables is

$$[C_1] \underset{k_{21}}{\overset{k_{12}}{\rightleftharpoons}} [C_2] \underset{k_{32}}{\overset{k_{23}}{\rightleftharpoons}} [C_3] \quad , \qquad (1)$$

where $C_1$ corresponds to the most open conformer, $C_2$ is similar to the substrate-enzyme complex and $C_3$ has an interdye distance much shorter than $C_2$. With this in mind we disregarded the cyclic model

$$
\begin{array}{ccc}
 & [C_3] & \\
\nearrow\!\!\swarrow & & \nwarrow\!\!\searrow \\
[C_1] & \rightleftharpoons & [C_2]
\end{array}
\qquad (2)
$$

due to the sequential closing of the enzyme from the most open to the most closed state, due to the unseen number of slow transitions along the $C_3$ to $C_1$, and physical restrains observed in the structural models (PDB ID 172L and 148L). This limits the return process of $[C_1] \rightleftharpoons [C_3]$, because there is no evidence of other intermediate states. The only remaining possibility is the unlikely scenario that after reaching the compact state, the enzyme unfolds and refolds very fast, so that we do not capture the process. Thus, given this unlikely set of events, we maintain the assumption of the successive closing of the enzyme.

Our goal is to extract the reaction rates ($k_{12}$, $k_{21}$, $k_{23}$, $k_{32}$) from our experimental observables. To solve this, first we need to write the rate matrix $K$ for the system described in Supplementary Equation (3).

$$K = \begin{pmatrix} -k_{12} & k_{21} & 0 \\ k_{12} & -(k_{21}+k_{23}) & k_{32} \\ 0 & k_{23} & -k_{32} \end{pmatrix}. \qquad (3)$$

The two eigenvalues of $K$ correspond to the two observables measured by fFCS.

$$\frac{1}{t_{R1,2}} = \frac{1}{2}\cdot(k_{12}+k_{21}+k_{23}+k_{32}) \pm \sqrt{(k_{12}+k_{21}+k_{23}+k_{32})^2 - 4\cdot(k_{12}k_{23}+k_{12}k_{32}+k_{21}k_{32})}\,. \qquad (4)$$

The time evolution of the system on Supplementary Equation (3) is defined by

$$\frac{d}{dt}[C_i](t) = K_{ij}[C_i](t), \qquad (5)$$

which has an analytical solution on the form of

$$[C_i](t) = \mathbf{C}_0 \exp(K_{ij}t), \qquad (6)$$

where $C_0$ is the $i$-th eigenvector. At equilibrium, or $t \rightarrow \infty$, the equilibrium fractions for each conformer can be obtained analytically and are given by

$$[C_i] = \begin{pmatrix} \dfrac{k_{21}\cdot k_{32}}{k_{21}\cdot k_{32}+k_{12}\cdot(k_{23}+k_{32})} \\ \dfrac{k_{12}\cdot k_{32}}{k_{21}\cdot k_{32}+k_{12}\cdot(k_{23}+k_{32})} \\ \dfrac{k_{12}\cdot k_{23}}{k_{21}\cdot k_{32}+k_{12}\cdot(k_{23}+k_{32})} \end{pmatrix}. \qquad (7)$$

Note that $[C_3] = 1-([C_1]+[C_2])$. These fractions are obtained by fluorescence decay analysis as done in Supplementary Methods. The reaction rates ($k_{12}$, $k_{21}$, $k_{23}$, $k_{32}$) can be expressed in terms of the equilibrium fractions ($x_1 = [C_1]$, $x_2 = [C_2]$, $x_3 = [C_3]$) and the relaxation times ($t_{R1}$ and $t_{R2}$).

The analytical solution of this system has two solutions:

$$k_{12}^{(\pm)} = \frac{[C_2]\cdot\left(\frac{1}{t_{R1}}+\frac{1}{t_{R2}}\right) \pm \left([C_2]\cdot\left(\frac{4\cdot([C_1]-1)\cdot([C_1]+[C_2])}{t_{R1}\cdot t_{R2}} + [C_2]\cdot\left(\frac{1}{t_{R1}}+\frac{1}{t_{R2}}\right)^2\right)\right)^{1/2}}{2\cdot([C_1]+[C_2])},$$

$$k_{21}^{(\pm)} = \frac{[C_1]\cdot\left([C_2]\cdot\left(\frac{1}{t_{R1}}+\frac{1}{t_{R2}}\right) \pm \left([C_2]\cdot\left(\frac{4\cdot([C_1]-1)\cdot([C_1]+[C_2])}{t_{R1}\cdot t_{R2}} + [C_2]\cdot\left(\frac{1}{t_{R1}}+\frac{1}{t_{R2}}\right)^2\right)\right)^{1/2}\right)}{2\cdot[C_2]\cdot([C_1]+[C_2])},$$

$$k_{23}^{(\pm)} = \frac{([C_1]+[C_2]-1)\cdot\left([C_2]\cdot\left(\frac{1}{t_{R1}}+\frac{1}{t_{R2}}\right) \mp \left([C_2]\cdot\left(\frac{4\cdot([C_1]-1)\cdot([C_1]+[C_2])}{t_{R1}\cdot t_{R2}} + [C_2]\cdot\left(\frac{1}{t_{R1}}+\frac{1}{t_{R2}}\right)^2\right)\right)^{1/2}\right)}{2\cdot[C_2]\cdot([C_1]-1)},$$

$$k_{32}^{(\pm)} = \frac{-[C_2]\cdot\left(\frac{1}{t_{R1}}+\frac{1}{t_{R2}}\right) \pm \left([C_2]\cdot\left(\frac{4\cdot([C_1]-1)\cdot([C_1]+[C_2])}{t_{R1}\cdot t_{R2}} + [C_2]\cdot\left(\frac{1}{t_{R1}}+\frac{1}{t_{R2}}\right)^2\right)\right)^{1/2}}{2\cdot([C_1]-1)} \quad . \tag{8}$$

To complete the model we need to add the static fraction of ~ 5%. We assigned this static fraction to conformer $C_{3t}$, which is identical in FRET to the state $C_{3d}$. We split the fraction of $C_3$ into these two populations. The final reaction model can be expressed as

$$[C_1] \underset{k_{21}}{\overset{k_{12}}{\rightleftharpoons}} [C_2] \underset{k_{32}}{\overset{k_{23}}{\rightleftharpoons}} [C_{3d}] \underset{k_{43}}{\overset{k_{34}}{\rightleftharpoons}} [C_{3t}] \quad . \tag{9}$$

Where $k_{34}$ = 0.003 $ms^{-1}$ and $k_{43}$ = 0.008 $ms^{-1}$ were empirically determined but satisfy the condition that they have to be smaller than 0.01 $ms^{-1}$.

We justify the existence of the $C_{3t}$ population because this state accumulated in MFD histograms over the observation time (~ms), and the population of this state lies over the static FRET line. We note that $C_{3t}$ does not increase over the period of the experiment. Hence, this state must be in slow equilibrium (>10 ms) with the rest of the network as reflected by fFCS.

With all the determined rates, we did Brownian dynamics simulations as described in main text. The single-molecule MFD histograms for the simulated data shown in Supplementary Figure 4 and corresponds to the experimental data shown in Figure 3.

Note that is the full cycle were used, this analytical formalism would not have been possible, because there are more parameters unknown that experimental observables.

### 3-3. Simulation of the FRET data in complex kinetic schemes

To describe the experimental 2D histogram a four-state scheme was used. First, we calculated FRET histograms [8] where a kinetic model with discrete conformations was assumed. The transition between the states is described by rate equations. The probability for the system to be in state $i$ at time $t$, $\boldsymbol{p}_i(t)$, satisfies a set of rate equations, which can be written in matrix notation as:

$$\frac{d\boldsymbol{p}}{dt} = \boldsymbol{K}\cdot\boldsymbol{p} \tag{10}$$

where $\boldsymbol{p}$ is a column vector with the components $\boldsymbol{p}_i(t)$ and $\boldsymbol{K}$ is a transition rate matrix representing the rate constants for the transitions between states $i$ and $j$. At long times, $\boldsymbol{p}(t)$

approaches its equilibrium value, $\boldsymbol{p}_{eq}$. The vector of the equilibrium populations $\boldsymbol{p}_{eq}$ is normalized to 1 and satisfies $\boldsymbol{K} \cdot \boldsymbol{p}_{eq} = 0$. For each burst, the mean averaged efficiency $\langle E \rangle$ and the average fluorescence weighted lifetime $\langle \tau_{D(A)} \rangle_f$ can be calculated by:

$$\langle E \rangle = \frac{\sum t_i \cdot E_i}{t_{burst}} \quad (11) \qquad \text{and} \qquad \langle \tau_{D(A)} \rangle_f = \frac{\sum t_i(\boldsymbol{K}) \cdot \tau_i^2}{\sum t_i(\boldsymbol{K}) \cdot \tau_i} \quad (12)$$

where $t_i(\boldsymbol{K})$ is time spent by a molecule in state $i$ within the duration of the burst and depends on the transition rate matrix $\boldsymbol{K}$; $E_i$ is the FRET efficiency of the $i$-th state; $t_{burst}$ is the duration of the burst and $\tau_i$ is the fluorescence lifetime of the $i$-th state. Practically, each burst has certain duration and number of photons, which were chosen arbitrary from experimentally measured $t_{burst}$ (duration time) vs. $N$ (number of photons) 2D histogram. The residence times by each molecule in different states were calculated using Gillespie algorithm for continuous-time Markov Chain. Then, the average fluorescence lifetime $\langle \tau_{D(A)} \rangle_f$ for each burst was calculated by Monte-Carlo simulation of fluorescence emission given FRET efficiencies of each state. Stationary (equilibrium) populations of states were obtained by solving interstate transition dynamics matrix and the residence times obtained on previous step. The descriptions for the vector $\boldsymbol{p}$ and the rate matrix $\boldsymbol{K}$ (resulting into the equilibrium fractions for the state $i$, $\boldsymbol{p}_{eq,i}$) and the experimental observables, $E$ and $\tau$, used in the simulations are shown in the Table S4a. For plotting, $E$ was converted to $F_D/F_A$ ratio. The simulation procedure was repeated for a high number of bursts to generate $F_D/F_A$ vs $\langle \tau_{D(A)} \rangle_f$ 2D histogram (Supplementary Figure 4b-c). The resulting 1D and 2D histograms were compared to the experimental data, yielding a $\chi^2$ parameter for each simulation and histogram. To test the significance of the difference in $\chi^2$, we performed F-test as described above. The resulting values are combined in the Table S4b.

To estimate our errors on determining the rates we considered the 2σ confidence interval in determining the population fractions (Supplementary Table 2h) and the 2σ confidence interval in determining the relaxation times by fFCS (Table S4c). Taking those extremes we estimated the error and computed the reaction rate constants for Figure 7 in the main text according to Supplementary Equation (8).

## Supplementary Note 4

### Fluorescence decay analysis of single and double labeled T4 lysozyme

Selected mutants were labeled in two configurations (DA) and (AD), D for donor (Alexa488) and A for acceptor fluorophore (Alexa647). The order of the letters represents the position of the fluorophore. The first letter represents the label of the keto handle in the N-terminal subdomain and the second position corresponds to the thiol reaction for labeling in the C-terminus, except for the double cysteine mutant.

Each sample was measured in eTCSPC as described in the materials and methods section and analyzed with three different models. As the fluorophores are connected to T4L by long and flexible linkers (Supplementary Methods) the assumption of a static, fixed interdye distance does not reflect the actual sample property. In fact, the flexible linkers assure a free rotational motion of the fluorophore, which allows to assume $\langle \kappa^2 \rangle = 2/3$ (verified by corresponding anisotropy measurements of each dye, see Supplementary Table 5a-d). Yet, this conformational flexibility leads to a distribution of interdye distances on the timescale of

FRET. A proper model for describing our sample properties has to consider this distribution. Here, we modeled this interdye distance distribution $\langle R_{DA} \rangle$ with a Normal distribution (Supplementary Equations (36)) (Supplementary Table 2b-f). The best consistent model based on our experimental data and statistical analysis is that three continuous distance distributions are needed to describe all T4L variants.

To reach to the conclusion that three continuous distance distributions are needed to describe all T4L variants, first we needed to characterize the donor and acceptor fluorescence quantum yield $\Phi_{FD(0)}$ and $\Phi_{FA}$, respectively. A summary table of these is shown in Supplementary Table 2a. Table S2b summarizes the result of the two continuous distance distribution model with free amplitudes. The best fit with three continuous distance distribution is summarized in Supplementary Table 2d-g; decays are shown in Supplementary Figure 5a. The fit results for the functional variants are summarized in Supplementary Table 2h.

Using only the two-state model and comparing the modeled distances using PDBID 172L and 148L for the two states showed that our data cannot be correlated with the structural information from the two crystallographic structures (Supplementary Figure 5b, c).

## Supplementary Note 5
## Characterization of functional T4L variants
### 5-1. Catalytic activity of S44pAcF I150C T26E

The ability to process the selected substrate (peptidoglycan from *Micrococcus luteus*) of the mutants was monitored by reverse phase chromatography. Prior to use, the purchased peptidoglycan (Sigma-Aldrich, Switzerland) was purified as described by Maeda in 1980 [9] to remove minor fluorescent impurities. Double-labeled mutants (1 µM) were incubated with 3 mg/mL of substrate and allowed to react for several hours in 50 mM sodium phosphate buffer, 150 mM NaCl at pH 7.5. Samples at different times were monitored under a reverse phase HPLC at 495 nm. In this way we can identify the labeled lysozyme. Typical examples for the processing of substrate are shown in Supplementary Figure 8. Supplementary Figure 8a shows the elution profile of the peptidoglycan monitored at 215 nm. Multiple peaks from 10 to 14 min appear. In the same panel the elution of the T26E/S44pAcF/I150C-(AD) monitored at 215 nm is shown as incubated with the peptidoglycan. For better contrast of the shift in populations the absorbance was measured at the maximum for the AlexaFluor488 (495 nm). This is shown in Supplementary Figure 8b. After 260 minutes this mutant is fully saturated with the substrate.

### 5-2. Single-molecule experiments in the presence of substrate

For the variant E11A/S44C/I150C we carried out two-color excitation experiments, in which we alternately excited the donor and the acceptor fluorophore (PIE) [5]. Thus, we could sort out the molecules carrying only one type of fluorophore, a disadvantage of the unspecific labeling. However, this allowed us also directly identifying the bursts stemming from the peptidoglycan. Supplementary Figure 7a-b show the green to red fluorescence signal vs. the stoichiometry $S$ of E11A/S44C/I150C in the absence and presence of substrate. D-Only labeled molecules are located at $S = 0$, A-Only molecules at $S = 1$ and DA labeled molecules are centered at $S = 0.5$ The peptidoglycan appears as an additional population at $S = 0.8$.

Supplementary Figure 7c shows that the brightness and burst duration distribution of this variant are nearly identical in the absence and presence of substrate.
For the variant T26E/S44pAcF/I150C only single-color excitation experiments were performed. The brightness and burst duration distributions of the FRET and the subset of high-FRET bursts are shown in Supplementary Figure 7e-h. To avoid the contamination with bursts from aggregates, we selectively only considered bursts shorter than 5 ms in the further analysis.

**Supplementary Note 6**
**Challenges of smFRET measurement and their solution**
Most of the potential problems with smFRET come from the complexities associated with the labels. We list the solution for potential label artifacts, and how our approaches and considerations allow us to draw conclusions, artifact free, of our data.
1) Labeling influence on enzymatic work: HPLC on the T26E/S44pAcF/150C-(DA) and –(AD) mutants show that they can process the peptidoglycan to keep the substrate bound. Non-functional mutants stayed non-functional after labeling (E11A/S44C/I150C-(DA) and R137E/S44pAcF/I150C-(DA)).
2) Local quenching of Donor:
eTCSPC: In ensemble measurements, local quenching is observed by changes in the average lifetime of the donor. The multi-exponential fluorescence decays of the donor only labeled variants reflect the presence of quenched states. At these states, the fluorophore senses a different environment. Most likely these differences represent various conformations of the protein.
smFRET: Donor quenching, as in the case of eTCSPC, shifts the average donor lifetime towards shorter lifetimes. FRET lines are corrected for the multi-exponential properties of the donor decay.
fFCS: We use the multi-exponential time-resolved fluorescence decay information to generate different filters to calculate the species cross-correlation. Although, protein dynamics can be extracted from single label variants, the structural information is lost. This is only possible from the FRET labeled samples.
3) Triplet-state of Donor:
eTCSPC: Triplet state are long lived compared to the fluorescence lifetime. Therefore, on ensemble time-resolved fluorescence decays this effect is not visible.
smFRET: Triplet or dark states kinetics are short-lived compared to the burst duration.
fFCS: In a classical FCS experiment triplet or dark states appear as a “bunching” term in the correlation function. In fFCS we do not correlate fluctuations on signal, but rather we correlate fluctuations of species. In our case, they correspond to different conformations of T4L. We assume that triplet/dark states are not coupled to the conformations or the selected pseudo species. In other words, the photo-physics of the dye is independent of the conformation in which the molecule is. With this in mind, the *sCCF* will have positive and negative contributions from each species resulting in the fact that the “bunching” term is not present. We know that increasing the power can increase the triplet amplitude. To test this, we measured the sCCF of T4L-(DA) at different powers at objective and we did not observe any major differences in the relaxation times $t_{R1}$ and $t_{R2}$ (Supplementary Figure 12) or shape

of the *sCCF*. We also tested the addition of triplet quenchers Cycloocta-1,3,5,7-tetraenecarboxylic acid (COTc) but did not observe major deviations (Supplementary Figure 12a-d).

4) Acceptor cis-trans isomerization:

eTCSPC: If FRET to *cis* and *trans* is different the donor decay would reflect the *cis-trans* population. We assume that this effect is small therefore not visible.

smFRET: This effect can be observed as acceptor quenching. The reason is that the *cis* state is dark. Spending more time in the *cis* state will reduce the overall counts observed from the acceptor. This effect can be seen in the two dimensional histograms as a vertical shift of the islands position on $F_D/F_A$ vs. lifetime $\langle\tau_{D(A)}\rangle_f$ representation.

fFCS: For fFCS we correlate only photons emitted by the donor fluorophore. Changes in the brightness of the acceptor are not correlated. However, something that can happen is that the absorption of the energy transferred from the donor can be different for *cis* and *trans* states. This is something that was not tested. But as in the case of the donor triplet we assume that, even in the case in which this occurs, the photophysics dynamics of the acceptor dye is decoupled from the conformational dynamics of the molecule.

5) Dye mobility:

eTCSPC: Dye mobility occurs at slower timescales than the time-resolved fluorescence decay of the fluorophore. For this reason, it is better to consider FRET due to all configurations of fluorophore positions during time-resolved fluorescence decays. We take this into consideration by having a distribution of distances instead of single lifetimes to identify each conformational state. These are included in the treatment of the FRET lines. In order to do so, ensemble time resolved anisotropy decays were measured. We assumed that fluorophore mobility follows the “wobble in a cone” model [10]. Table S5a-c summarizes the residual anisotropies ($r_\infty$) of D - donor, A - acceptor and A(D) - the sensitized by FRET emission of acceptor that were used to calculate dye order parameters and $\kappa^2$ distributions (Table S5d) according to refs. [3] [10] (Eq. 9 and 10 in Sindbert *et al.*). The assumption is that fluorophores move according to the “wobble in a cone” model. According to all distributions the assumption of $\kappa^2 = 2/3$ is very well justified.

smFRET: In smFRET one can inspect the anisotropy $r_{sc}$ vs. lifetime $\langle\tau_{D(A)}\rangle_f$ histograms. If anisotropy is too high then one would expect that the dye can have restricted mobility.

fFCS: The mobility of the dye alone is better resolved using a complete FCS technique [11].

6) FPS provides also a consistent view of the conformational states of T4L. Each distinct set of conformer specific FRET restrains are within the expected uncertainty of our tools. In addition, the kinetics found in all our variants are consistent with two global relaxation time ($t_{R1}$ = ~4 μs, $t_{R2}$ = ~ 230 μs) and the expected three conformational states.

7) Thermodynamic stability and proper folding of our mutants were verified by chemical denaturation using urea.

8) Fluorescence intensity decay were fit with various models and gave a consistent view of three FRET induced donor lifetimes or two FRET induced donor lifetimes where only one would be expected if the conformer $C_3$ did not exist.

9) Limitations on hybrid FRET: Current limitations of hybrid FRET include the introduction of labels via site directed mutagenesis. Not all systems are resistant to extensive mutations

and maintain their stability. In terms of the size of molecules under study, the limitations will be determined by the level of resolution that it is desired. Larger molecules would require a larger set of FRET measurements and bigger markers, including the inclusion of fluorescent proteins would cause larger uncertainties. Although, nothing limits the ability to do measurements in complex environments (crowder or viscous solutions and even in live cells), one must assure that proper controls are satisfied. Of particular interest are measurements in living cells, where the labeled molecules have to be introduced without damaging the liability of the cells.[12]

**Supplementary Note 7**
**Original data available on Zenodo**

The following files are available on Zenodo under DOI 10.5281/zenodo.3376527:

- eTCSPC_wildtype.zip contains all eTCSPC FRET data including reference measurements used for derivation of DA distances.
- Single_molecule_wildtype.zip contains raw single-molecule data used for filtered FCS and MFD analysis: calibration measurements (e.g. for detection efficiency, instrument response function (IRF), background, and g-factor calibration) and measurement data.
- Single_molecule_functional_variants.zip contains raw single-molecule data for derivation of the kinetic scheme.
- EPR_wildtype.zip contains EPR data.
- FRET_screening_of_PDB_structures.zip contains a table of experimental distances and errors for each DA pair (corresponding to Supplementary Table 2), and a file with an overview on the FRET screening of the PDB structures.

Each of the subfolders in eTCSPC_wildtype.zip, Single_molecule_wildtype.zip and Single_molecule_functional_variants.zip for a DA pair X-Y contain a X-Y.yml file describing the setup and referencing all data relevant for the analysis of the measurement.

| Filename of meta file on Zenodo | description |
|---|---|
| eTCSPC\5-44\5-44.yml | eTCSPC data for variant E5pAcF/S44C |
| eTCSPC\5-132\5-132.yml | eTCSPC data for variant E5pAcF/N132C |
| eTCSPC\8-69\8-69.yml | eTCSPC data for variant R8pAcF/Q69C |
| eTCSPC\8-86\8-86.yml | eTCSPC data for variant R8pAcF/P86C |
| eTCSPC\8-119\8-119.yml | eTCSPC data for variant R8pAcF/R119C |
| eTCSPC\8-132\8-132.yml | eTCSPC data for variant R8pAcF/N132C |
| eTCSPC\19-69\19-69.yml | eTCSPC data for variant K19pAcF/Q69C |
| eTCSPC\19-86\19-86.yml | eTCSPC data for variant K19pAcF/P86C |
| eTCSPC\19-119\19-119.yml | eTCSPC data for variant K19pAcF/R119C |
| eTCSPC\19-132\19-132.yml | eTCSPC data for variant K19pAcF/N132C |
| eTCSPC\22-127\22-127.yml | eTCSPC data for variant E22pAcF/D127C |
| eTCSPC\36-86\36-86.yml | eTCSPC data for variant S36pAcF/P86C |
| eTCSPC\36-132\36-132.yml | eTCSPC data for variant S36pAcF/N132C |
| eTCSPC\44-69\44-69.yml | eTCSPC data for variant S44pAcF/Q69C |
| eTCSPC\44-86\44-86.yml | eTCSPC data for variant S44pAcF/P86C |
| eTCSPC\44-119\44-119.yml | eTCSPC data for variant S44pAcF/R119C |
| eTCSPC\44-127\44-127.yml | eTCSPC data for variant S44pAcF/D127C |
| eTCSPC\44-132\44-132.yml | eTCSPC data for variant S44pAcF/N132C |
| eTCSPC\44-150\44-150.yml | eTCSPC data for variant S44pAcF/I150C |
| eTCSPC\55-69\55-69.yml | eTCSPC data for variant N55pAcF/Q69C |
| eTCSPC\55-119\55-119.yml | eTCSPC data for variant N55pAcF/R119C |
| eTCSPC\55-132\55-132.yml | eTCSPC data for variant N55pAcF/N132C |
| eTCSPC\55-150\55-150.yml | eTCSPC data for variant N55pAcF/I150C |
| eTCSPC\60-86\60-86.yml | eTCSPC data for variant K60pAcF/P86C |
| eTCSPC\60-119\60-119.yml | eTCSPC data for variant K60pAcF/R119C |
| eTCSPC\60-132\60-132.yml | eTCSPC data for variant K60pAcF/N132C |
| eTCSPC\60-150\60-150.yml | eTCSPC data for variant K60pAcF/I150C |
| eTCSPC\69-86\69-86.yml | eTCSPC data for variant Q69pAcF/P86C |
| eTCSPC\69-119\69-119.yml | eTCSPC data for variant Q69pAcF/R119C |
| eTCSPC\69-132\69-132.yml | eTCSPC data for variant Q69pAcF/N132C |

| | |
|---|---|
| eTCSPC\69-150\69-150.yml | eTCSPC data for variant Q69pAcF/I150C |
| eTCSPC\70-119\70-119.yml | eTCSPC data for variant D70pAcF/R119C |
| eTCSPC\70-132\70-132.yml | eTCSPC data for variant D70pAcF/N132C |

| Filename of meta file on Zenodo | description |
|---|---|
| Single_molecule_wildtype\5-44\5-44.yml | Single-molecule data for variant E5pAcF/S44C |
| Single_molecule_wildtype\5-44\5-44.yml | Single-molecule data for variant E5pAcF/S44C |
| Single_molecule_wildtype\5-132\5-132.yml | Single-molecule data for variant E5pAcF/N132C |
| Single_molecule_wildtype\8-69\8-69.yml | Single-molecule data for variant R8pAcF/Q69C |
| Single_molecule_wildtype\8-86\8-86.yml | Single-molecule data for variant R8pAcF/P86C |
| Single_molecule_wildtype\8-119\8-119.yml | Single-molecule data for variant R8pAcF/R119C |
| Single_molecule_wildtype\8-132\8-132.yml | Single-molecule data for variant R8pAcF/N132C |
| Single_molecule_wildtype\19-69\19-69.yml | Single-molecule data for variant K19pAcF/Q69C |
| Single_molecule_wildtype\19-86\19-86.yml | Single-molecule data for variant K19pAcF/P86C |
| Single_molecule_wildtype\19-119\19-119.yml | Single-molecule data for variant K19pAcF/R119C |
| Single_molecule_wildtype\19-132\19-132.yml | Single-molecule data for variant K19pAcF/N132C |
| Single_molecule_wildtype\22-127\22-127.yml | Single-molecule data for variant E22pAcF/D127C |
| Single_molecule_wildtype\36-86\36-86.yml | Single-molecule data for variant S36pAcF/P86C |
| Single_molecule_wildtype\36-132\36-132.yml | Single-molecule data for variant S36pAcF/N132C |
| Single_molecule_wildtype\44-69\44-69.yml | Single-molecule data for variant S44pAcF/Q69C |
| Single_molecule_wildtype\44-86\44-86.yml | Single-molecule data for variant S44pAcF/P86C |
| Single_molecule_wildtype\44-119\44-119.yml | Single-molecule data for variant S44pAcF/R119C |
| Single_molecule_wildtype\44-127\44-127.yml | Single-molecule data for variant S44pAcF/D127C |
| Single_molecule_wildtype\44-132\44-132.yml | Single-molecule data for variant S44pAcF/N132C |
| Single_molecule_wildtype\44-150\44-150.yml | Single-molecule data for variant S44pAcF/I150C |
| Single_molecule_wildtype\55-69\55-69.yml | Single-molecule data for variant N55pAcF/Q69C |
| Single_molecule_wildtype\55-119\55-119.yml | Single-molecule data for variant N55pAcF/R119C |
| Single_molecule_wildtype\55-132\55-132.yml | Single-molecule data for variant N55pAcF/N132C |
| Single_molecule_wildtype\55-150\55-150.yml | Single-molecule data for variant N55pAcF/I150C |
| Single_molecule_wildtype\60-86\60-86.yml | Single-molecule data for variant K60pAcF/P86C |
| Single_molecule_wildtype\60-119\60-119.yml | Single-molecule data for variant K60pAcF/R119C |
| Single_molecule_wildtype\60-132\60-132.yml | Single-molecule data for variant K60pAcF/N132C |
| Single_molecule_wildtype\60-150\60-150.yml | Single-molecule data for variant K60pAcF/I150C |
| Single_molecule_wildtype\69-86\69-86.yml | Single-molecule data for variant Q69pAcF/P86C |
| Single_molecule_wildtype\69-119\69-119.yml | Single-molecule data for variant Q69pAcF/R119C |
| Single_molecule_wildtype\69-132\69-132.yml | Single-molecule data for variant Q69pAcF/N132C |
| Single_molecule_wildtype\69-150\69-150.yml | Single-molecule data for variant Q69pAcF/I150C |
| Single_molecule_wildtype\70-119\70-119.yml | Single-molecule data for variant D70pAcF/R119C |
| Single_molecule_wildtype\70-132\70-132.yml | Single-molecule data for variant D70pAcF/N132C |
| | |
| Single_molecule_functional_variants\44-150\44-150.yml | Single-molecule data for function variant S44pAcF/I150C (identical to Single_molecule_wildtype\44-150\44-150.yml) |
| Single_molecule_functional_variants\44-150+pep\44-150+pep.yml | Single-molecule data for function variant S44pAcF/I150C with peptidoglycan |
| Single_molecule_functional_variants\44-150-E11A\44-150-E11A.yml | Single-molecule data for function variant S44pAcF/I150C/E11A |
| Single_molecule_functional_variants\44-150-E11A+pep\44-150-E11A+pep.yml | Single-molecule data for function variant S44pAcF/I150C/E11A with peptidoglycan |
| Single_molecule_functional_variants\44-150-R137E\44-150-R137E.yml | Single-molecule data for function variant S44pAcF/I150C/R137E |
| Single_molecule_functional_variants\44-150-T26E\44-150-T26E.yml | Single-molecule data for function variant S44pAcF/I150C/T26E |
| Single_molecule_functional_variants\44-150-T26E+pep\44-150-T26E+pep.yml | Single-molecule data for function variant S44pAcF/I150C/T26E with peptidoglycan |

The subfolders for eTCSPC measurements for DA pair X-Y contain the following files:

| Filename | Purpose |
|---|---|

| | |
|---|---|
| X-Y_DA.dat | Data for measurement of the FRET sample |
| X-Y_D0.dat | Data for donor-only reference measurement |
| IRF_DA.dat | Instrument response function for FRET measurement |
| IRF_D0.dat | Instrument response function for donor-only reference measurement |

The subfolders for a single-molecule measurement for DA pair X-Y contain the following subfolders:

| **Folder name**[1] | **Content** |
|---|---|
| "X-Y smd 0M" or "X Y DA sm" or "X_Y_0murea_sm" or "T4L_X_Y_FRET" or "T4L-X-Y-DA-smd" or "X_Y_da_sm" | Measurement data |
| "IRF" or "irf" or "H2O" or "water" or "h2o" | Instrument response function (IRF) |
| "DNA" or "dna" | Calibration measurement for detection efficiency |
| "pbs0M" or "pbs" or "buffer" or "buffer_HS" or "PBS" or "buffer_tween20" or "buff_0m" or "Buffer 00M" | Calibration measurement for background |
| "Rh101" and "Rh110" or "Rhod101" and "Rhod110" or "Rhod110thick" or "rho101" and "rho110" | Measurements of G-factors for the calibration of the anisotropy in the red (Rh101) and green (Rh110) detection channels |

[1] Folder names might vary between subfolders

## *Supplementary Methods*

### Multiparameter Fluorescence Detection (MFD)

MFD for confocal single molecule Förster Resonance Energy Transfer (smFRET) measurements was done using a 485 nm diode laser (LDH-D-C 485 PicoQuant, Germany, operating at 64 MHz, power at objective 110 µW) exciting freely diffusing labeled T4L molecule that passed through a detection volume of the 60X, 1.2 NA collar (0.17) corrected Olympus objective. The emitted fluorescence signal was collected through the same objective and spatially filtered using a 100 µm pinhole, to define an effective confocal detection volume. Then, the signal was divided into parallel and perpendicular components at two different colors ("green" and "red") through band pass filters, HQ 520/35 and HQ 720/150, for green and red respectively, and split further with 50/50 beam splitters. In total eight photon-detectors are used- four for green (τ-SPAD, PicoQuant, Germany) and four for red channels (APD SPCM-AQR-14, Perkin Elmer, Germany). A time correlated single photon counting (TCSPC) module (HydraHarp 400, PicoQuant, Germany) with a 1ps resolution in Time Tagged Time Resolved (TTTR – an application note regarding this data acquisition setting is found in Ref [13] (https://www.picoquant.com/images/uploads/page/files/14528/technote_tttr.pdf)) was used for data registration.

For smFRET measurements samples were diluted (buffer used 50 mM sodium phosphate, pH 7.5, 150 mM NaCl, 40 µM TROLOX and 1 µM unlabeled T4L) to pM concentration assuring ~ 1 burst per second. Collection time varied from several minutes up to 10 hours. To avoid drying out of the immersion water during the long measurements an oil immersion liquid with refraction index of water was used (Immersol, Carl Zeiss Inc., Germany). NUNC chambers (Lab-Tek, Thermo Scientific, Germany) were used with 500 µL sample volume. Standard controls consisted of measuring water to determine the instrument response function (IRF), buffer for background subtraction and the nM concentration green and red standard dyes (Rh110 and Rh101) in water solutions for calibration of green and red channels, respectively. To calibrate the detection efficiencies, we used a mixture solution of double labeled DNA oligonucleotides with known distance separation between donor and acceptor dyes.

### MFD burst analysis: Multiparameter FRET histograms and FRET-lines

Bursts were selected by 2σ criteria out of the mean background value with cut off times that vary from sample to sample with a minimum of 60 photons for each burst. Each burst was then processed and fitted using a maximum likelihood algorithm [14] using in house developed software (LabVIEW, National Instruments Co.). Fluorescent bursts were plotted in 2D histograms (Origin 8.6, OriginLab Co).

The relation FRET-efficiency $E$ and the species weighted average donor lifetimes $\langle\tau\rangle_x$ depends on the fluorescence quantum yields of the dyes ($\Phi_{FD(0)}$ and $\Phi_{FA}$ for donor and acceptor respectively) and implicitly on background ($\langle B_G\rangle$ and $\langle B_R\rangle$ for green and red channels), detection efficiencies ($g_G$ and $g_R$ for green and red respectively) and crosstalk ($\alpha$):

$$E = \frac{1}{1 + \frac{F_D / \Phi_{FD(0)}}{F_A / \Phi_{FA}}} = 1 - \frac{\langle \tau_{D(\mathrm{A})} \rangle_x}{\langle \tau_{D(0)} \rangle_x} \tag{13}$$

The corrected fluorescence ($F_D$ and $F_A$) depends on the detection efficiencies of green ($g_G$) and red ($g_R$) channels as follows:

$$F_D = \frac{S_G - \langle B_G \rangle}{g_G}, \tag{14}$$

$$F_A = \frac{S_R - \alpha F_G - \langle B_R \rangle}{g_R}, \tag{15}$$

where the total signal in green and red channels are $S_G$ and $S_R$, respectively. The ratio ($F_D/F_A$) is weighted by the species fractions.

In Supplementary Equation (13), brackets $\langle ... \rangle_x$ represent averaging over all lifetime components. For the species $\tau^{(i)}$ weighted by its population fraction $x^{(i)}$, these averages are given by:

$$\langle \tau_{D(0)} \rangle_x = \sum_i x^{(i)} \tau_{D(0)}^{(i)} \quad \text{and} \quad \langle \tau_{D(\mathrm{A})} \rangle_x = \sum_i x^{(i)} \tau_{D(\mathrm{A})}^{(i)} \tag{16}$$

Above $\langle \tau_{D(\mathrm{A})} \rangle_x$ and $\langle \tau_{D(0)} \rangle_x$ are the species averaged fluorescence lifetimes of the donor in presence and absence of an acceptor, respectively.

In sm FRET experiments approximately ~100 green photons per burst are detected. Hence, only the average time since excitation is reliably determined experimentally by the maximum likelihood estimators (MLE) for individual bursts. This time is weighted by the fluorescence intensity and hence, relates to the fluorescence lifetime components by:

$$\langle \tau_{D(0)} \rangle_f = \frac{\sum_i x^{(i)} \tau_{D(0)}^{(i)\,2}}{\sum_i x^{(i)} \tau_{D(0)}^{(i)}} \quad \text{and} \quad \langle \tau_{D(\mathrm{A})} \rangle_f = \frac{\sum_i x^{(i)} \tau_{D(A)}^{(i)\,2}}{\sum_i x^{(i)} \tau_{D(A)}^{(i)}}, \tag{17}$$

We call these lifetimes fluorescence weighted average lifetimes.

The two averaged observables $E$ (in Supplementary Equations (13)) and $\langle \tau_{D(A)} \rangle_f$ can be related to each other. We call a line describing a theoretical relation of the two a "FRET-line". Such FRET-lines are projections of a parametrization of a multi-dimensional lifetime distribution to a two-dimensional plane using either the transfer-efficiency $E$ or $F_D/F_A$ as one and $\langle \tau_{D(\mathrm{A})} \rangle_f$ as second axis.

Fluorophores are moving entities coupled to biomolecules at specific places via flexible linkers. Therefore, for single protein conformations a DA-distance distribution has to be considered. For simplicity, we use normal distributions to describe the DA-distance distributions. If the donor and acceptor interfluorophore average distance is $\langle R_{\mathrm{DA}} \rangle$, the corresponding DA-distance distribution is:

$$p(R_{DA}) = \frac{1}{w_{\mathrm{DA}} \sqrt{\pi / 2}} \exp\left( -2 \left[ \frac{R_{\mathrm{DA}} - \langle R_{DA} \rangle}{w_{DA}} \right]^2 \right), \tag{18}$$

Here, $w_{DA}$ is the width of the DA-distance distribution attributed to the broadening due to the linker-flexibility set to a physical meaningful value of 12 Å [3]. Using the Förster-relationship $\tau_{D(\mathrm{A})}(R_{DA}) = \tau_{D(0)} \cdot \left(1 + (R_0/R_{DA})^6\right)^{-1}$ and the following integrals:

$$\langle \tau_{D(\mathrm{A})} \rangle_x = \int \tau_{D(\mathrm{A})}(R_{DA}) p(R_{DA}) dR_{DA}, \tag{19}$$

$$\langle \tau_{D(\mathrm{A})} \rangle_f = \frac{\int \left(\tau_{D(\mathrm{A})}(R_{DA})\right)^2 p(R_{DA}) dR_{DA}}{\langle \tau_{D(\mathrm{A})} \rangle_{x,L}}, \tag{20}$$

This (Supplementary Equation (18)) distribution can be projected to a point in the $E$-$\langle\tau_{D(\mathrm{A})}\rangle_{\mathrm{f}}$ plane. If the average DA distance $\overline{\langle R_{DA} \rangle}$ is varied within a given range (i.e. $[0,\infty]$) a line within the $E$-$\langle\tau_{D(\mathrm{A})}\rangle_{\mathrm{f}}$ plane is obtained. Such a line we call a static FRET-line, as it is valid for all molecules with given (single) conformation, irrespectively of the mean DA-separation, $\overline{\langle R_{DA} \rangle}$.

To describe molecules, which are interconverting between two states with mean distances $\overline{\langle R_{DA} \rangle}^{(1)}$ and $\overline{\langle R_{DA} \rangle}^{(2)}$ and fractions $x^{(1)}$ and $x^{(2)}=1-x^{(1)}$, by a line in the $E$-$\langle\tau_{D(\mathrm{A})}\rangle_{\mathrm{f}}$ plane we use the following distance distribution:

$$p(R_{DA}) = \frac{x_{DA}^{(1)}}{w_{\mathrm{DA}}\sqrt{\pi/2}} \exp\left(-2\left[\frac{R_{\mathrm{DA}} - \langle R_{DA}^{(1)} \rangle}{w_{DA}}\right]^2\right) + \frac{1 - x_{DA}^{(1)}}{w_{\mathrm{DA}}\sqrt{\pi/2}} \exp\left(-2\left[\frac{R_{\mathrm{DA}} - \langle R_{DA}^{(2)} \rangle}{w_{DA}}\right]^2\right) \tag{21}$$

To obtain a "dynamic" FRET-line, which is valid for a molecule in exchange between these two states, the fraction $x^{(1)}$ is varied within the range [0, 1] and the position in the $E$-$\langle\tau_{D(\mathrm{A})}\rangle_{\mathrm{f}}$ plane is calculated using the Supplementary Equations (19), (20) and (13).

**Guidelines for reading MFD histograms**

Several guidelines are needed to properly read MFD histograms. A short list is presented here.

*I*) Donor only population is shown at low $E$ with lifetime ~ 4 ns (donor-only for Alexa488).

*II*) High FRET appears at shorter lifetimes when the fluorescence of acceptor is high ($E \rightarrow 1$).

*III*) Static FRET states follow a theoretical line that accounts for dye linker mobility called "static FRET line" [15].

*IV*) A molecule that exchanges conformations at timescales faster than the diffusion time emits a burst of photons whose mixed fluorescence is characterized by the fluorescence average lifetime. Elongation of peaks in $E$-$\langle\tau_{D(\mathrm{A})}\rangle_{\mathrm{f}}$-histograms and deviation from static FRET-lines are an indication for slow conformational dynamics processes on the hundreds of microseconds.

We inspect the signal over the duration of the measurement. Typically, we find stable signal over 1 hr and 10 hrs. Additionally, we minimize unlikely effects of multimolecule events by comparing the difference in the burst duration in donor and acceptor channels ($|T_G|$-$|T_R|$) or aggregates (e. g. via burst duration or the diffusion time component of the correlated

molecular bursts) and impurities due to e.g. free, unattached fluorophores (e.g. by plotting the scatter-corrected anisotropy vs. $\langle \tau_{D(A)} \rangle_f$).

**Filtered Fluorescence Correlation Spectroscopy**

In fluorescence correlation spectroscopy (FCS) information on fluctuating systems is obtained by calculating the correlation function [16,17]:

$$ {}^{A,B}G(t_c) = 1 + \frac{\langle \delta^A S(t) \cdot \delta^B S(t+t_c) \rangle}{\langle {}^A S(t) \rangle \cdot \langle {}^B S(t) \rangle} . \qquad (22)$$

where $t_c$ is the correlation time, ${}^{A,B}S(t)$ represents the detected intensity signal (number of detected photons per time interval) at channels $A$ or $B$, and $\delta^{A;B}S(t)$ corresponds to the deviation of the signal from the time average signal denoted as $\langle {}^{A;B}S(t) \rangle$. ${}^{A,B}G(t_c)$ is an auto-correlation function (*ACF*) if $A = B$ otherwise ${}^{A,B}G(t_c)$ is a cross-correlation (*CCF*).

The correlation function [18] [19] of a mixture of $n$ molecular static species is given by an weighted average:

$$ G(t_c) = 1 + \frac{1}{N} \cdot \frac{\sum_i^n x^{(i)} \cdot (Q^{(i)})^2 \cdot G_{diff}^{(i)}(t_c)}{\left( \sum_i^n x^{(i)} \cdot Q^{(i)} \right)^2} , \qquad (23)$$

where $G_{diff}^{(i)}(t_c)$ describes molecular diffusion. For a 3-dimensional Gaussian detection probability, $W(x,y,z) = \exp\left(-2\left(x^2+y^2\right)/\omega_0^2\right) \cdot \exp\left(-2\,z^2/z_0^2\right)$, $G_{diff}^{(i)}(t_c)$ is given by:

$$ G_{diff}^{(i)}(t_c) = \left(1 + \frac{t_c}{t_{diff}^{(i)}}\right)^{-1} \cdot \left(1 + \left(\frac{\omega_0}{z_0}\right)^2 \cdot \frac{t_c}{t_{diff}^{(i)}}\right)^{-\frac{1}{2}} , \qquad (24)$$

The $1/e^2$ radii in $x$ and $y$ or in $z$ direction are denoted by $\omega_0$ and $z_0$, respectively. The characteristic diffusion time $t_{diff}^{(i)}$ relates to the diffusion coefficient of each species $i$ $t_{diff}^{(i)} = \omega_0^2 / 4D^{(i)}$ . The amplitude of the correlation is scaled with the reciprocal of the average number of fluorescent particles $N$ in the confocal volume. Each molecular fraction $x^{(i)} = c^{(i)} / \sum_i c^{(i)}$ has a concentration $c^{(i)}$, and brightness $Q^{(i)}$.

To separate species, we use filtered FCS (fFCS) [7,20]. fFCS differs from standard FCS [16] and FRET-FCS [21] by interrogating the "species" (conformational states) fluctuations instead of photon count rates [21]. We define the species cross- correlation function (*sCCF*) as

$$ G^{(i,m)}(t_c) = \frac{\langle F^{(i)}(t) \cdot F^{(m)}(t+t_c) \rangle}{\langle F^{(i)}(t) \rangle \cdot \langle F^{(m)}(t+t_c) \rangle} = \frac{\left\langle \left( \sum_{j=1}^{d \cdot L} f_j^{(i)} \cdot S_j(t) \right) \cdot \left( \sum_{j=1}^{d \cdot L} f_j^{(m)} \cdot S_j(t+t_c) \right) \right\rangle}{\left\langle \sum_{j=1}^{d \cdot L} f_j^{(i)} \cdot S_j(t) \right\rangle \cdot \left\langle \sum_{j=1}^{d \cdot L} f_j^{(m)} \cdot S_j(t+t_c) \right\rangle} , \qquad (25)$$

where (*i*) and (*m*) are two selected "species" or "pseudospecies" in a mixture, where pseudospecies correspond to the equilibrium of two or more mixed species that are in fast exchange. A set of filters $f_j^{(i)}$ that depend on the arrival time of each photon after each excitation pulse is used. The signal $S_j(t)$, obtained via pulsed excitation, is recorded at each $j$

= 1 ... $L$ TCSPC-channel. The signal and filters per detector, $d$, are stacked in a single array with dimensions $d \cdot L$ for global minimization according to [7]. Filters are defined in such a way that the relative "error" difference between the photon count per species ($w^{(i)}$) and the weighted histogram $f_j^{(i)} \cdot H_j$ is minimized as defined in Supplementary Equation (26).

$$\left\langle \left( \sum_{j=1}^{d \cdot L} f_j^{(i)} \cdot H_j - w^{(i)} \right)^2 \right\rangle \to \min \,, \tag{26}$$

where brackets represent time averaging.

The requirement is that the decay histogram $H_j$ can be expressed as a linear combination of the conditional probability distributions $p_j^{(i)}$, such as $H_j = \sum_{i=1}^{n(=2)} w^{(i)} p_j^{(i)}$, with $\sum_{j=1}^{d \cdot L} p_j^{(i)} = 1$. Here, $p_j^{(i)}$ corresponds to the time resolved fluorescence decay of each selected pseudo-species $i$ (Supplementary Figure 9a, c). Using the definition of the fluorescence decays in Equation 26 it is then possible to find a weight value $w^{(i)}$ to satisfy the experimental observable fluorescence decay characteristic of the mixture $H_j$, and the corresponding filters per species $f_j^{(i)}$ . Examples are plotted in Supplemental Figure 9b, d.

Hence, the species cross- correlation $G^{(i,m)}(t_c)$ provides maximal contrast for intercrossing dynamics [7]. One major advantage of *sCCF* is that, if photophysical properties are decoupled from species selection, the intercrossing dynamics [21] is recovered with great fidelity.

To properly fit the species cross-correlation function, we used [7]

$$G(t_c) = 1 + \frac{1}{N} \cdot G_{diff}(t_c) \cdot [1 - G_K(t_c)], \tag{27}$$

where $G_K(t_c)$ is

$$G_K(t_c) = \sum_{t_{Ri}}^{t_{Rn}} A_{Ki} \exp(-t_c / t_{Ri}). \tag{28}$$

In Supplementary Equation (28) the summation is over $n$ reaction times $t_{Rn}$.

The same 3-dimensional Gaussian shaped volume element is assumed. We assume that $G_{diff}(t_c) = G_{diff}^{(i)}(t_c) = G_{diff}^{(m)}(t_c)$ take the form of Supplementary Equation (27). The normalized correlation function is presented as:

$$g(t_c) = N \cdot (G(t_c) - 1). \tag{29}$$

Filtered FCS requires prior knowledge of the time-resolved fluorescence and polarization decays for each species or pseudospecies. For a mixture of more than two species, we generated two decays corresponding to two "pseudo-species". Using the scatter profile as the excitation pulse, the parallel and perpendicular decay components ($F_{||}(t)$ and $F_{\perp}(t)$) for each "pseudo-species" were generated as

$$\begin{aligned} F_{||}(t) &= F(t) \cdot (1 + (2 - 3l_1) \cdot r(t)) / 3 \\ F_{\perp}(t) &= F(t) \cdot (1 - (1 - 3 \cdot l_2) \cdot r(t)) / 3 \end{aligned} \,, \tag{30}$$

where $F(t)$ is the time-resolved fluorescence decay at magic angle, and $l_1$ = 0.01758 and $l_2$ = 0.0526 are correction factors [22 23]. The anisotropy decay $r(t)$ is given by

$$r(t) = r_{0,ov} \exp(-t / \rho_{overall}) + r_{0,ba} \exp(-t / \rho_{backbone}) + r_{0,li} \exp(-t / \rho_{linker}) . \quad (31)$$

Background signal consists of dark counts (uniformly distributed over TCSPC channels) and scatter contribution.

**Ensemble Time Correlated Single Photon Counting with high precision**

Ensemble Time Correlated Single Photon Counting (eTCSPC) measurements were performed using either an IBH-5000U (IBH, Scotland) or a Fluotime 200 (Picoquant, Germany) system.

The excitation source of the IBH machine were a 470 nm diode laser (LDH-P-C470, Picoquant, Germany) operating at 10 MHz for donor excitation and a 635 nm (LDH-P-C635, Picoquant, Germany) for acceptor excitation. The excitation and emission slits were set to 2 nm and 16 nm, respectively. The excitation source of the Fluotime200 system was a white light laser (SuperK extreme, NKT Photonics, Denmark) operating at 20 MHz for both donor (485 nm) and acceptor (635 nm) excitation with excitation and emission slits set to 2 nm and 5 nm, respectively. Additionally, in both systems, cut-off filters were used to reduce the amount of scattered light (>500 nm for donor and >640 nm for acceptor emission).

For green detection, the monochromator was set to 520 nm and for red detection to 665 nm. All measurements were conducted under magic angle conditions (excitation polarizer 0°, emission polarizer 54.7°), except for anisotropy where the position of the emission polarizer was alternately set to 0° (VV) or 90° (VH).

In the IBH system, the TAC-histograms were recorded with a bin width of 14.1 ps within a time window of 57.8 ns, while the Fluotime200 was set to a bin width of 8 ps within a time window of 51.3 ns. Photons were collected up to a peak count of 100’000 corresponding in average to a total number of $30 \cdot 10^6$ photons. The instrument response function IRF (~230 ps FWHM for the IBH, ~ 150 ps for the Fluotime200) was collected under the same recording settings at the excitation wavelength of the sample without cutoff-filters using a scattering Ludox-dispersion, which yielded a comparable count rate as the later on measured samples.

For the IBH system, it was needed was performed before each measurement session a reference measurement with a continuous light signal to account for the differential non-linearity of the counting electronics. The recorded uncorrelated photons yield a reference histogram that is ideally constant. After recording of this measurement, the average number of photons in each time-bin is calculated. Next, the measurement was smoothed by a window function using a Hanning-filter with a window-size of 17 bins. The smoothed decay histogram was normalized to the previously calculated average number of photons. Instead of correcting the experimental histogram the model function is multiplied by the smoothed and normalized reference histogram to preserve the Poissonian statistics of the measured fluorescence intensity histograms of interest.

**Donor and acceptor fluorescence quantum yields**

Depending on the labeling position, the donor and acceptor fluorescence quantum yields vary and have been estimated for each sample (Supplementary Table 2a). We estimate $\Phi_{FD(0)}$ and $\Phi_{FA}$ of the fluorescent species by the species-averaged fluorescence lifetime $\langle \tau \rangle_x$ of donor or

acceptor, respectively. As reference samples we used Alexa488-labeled DNA $\langle \tau_{D(0)} \rangle_x$ = 4.0 ns, $\Phi_{FD(0)}$ = 0.8 and for the acceptor Alexa647, we used the similar dye Cy5 at Cy5-labeled DNA with $\langle \tau_A \rangle_x$ = 1.17 ns and $\Phi_{FA}$ = 0.32 [24] . This FRET pair has a Förster distance of 52 Å.

**Time-resolved fluorescence decay analysis**

*Model*

We model the fluorescence decay of the donor in the absence of FRET $F_{D(0)}(t)$ by a multi-exponential decay to account for sample specific differences of the donor reference samples

$$F_{D(0)}(t) = \sum_i x_{D(0)}^{(i)} \exp(-t/\tau_{D(0)}^{(i)}). \quad (32)$$

Here, $\tau_{D(0)}^{(i)}$ is the donor fluorescence lifetime and $x_{D(0)}^{(i)}$ are the pre-exponential factors.

Sample specific differences were considered in the analysis of the FRET samples by joint analysis where all donor species are quenched by the same FRET rate constant $k_{RET}$. Such model is correct if quenching does not change the donor radiative lifetime and if FRET is uncorrelated with quenching of the donor by its local environment. Under these conditions the donor fluorescence intensity decay in the presence of FRET $F_{D(A)}(t)$ factorizes into the donor fluorescence decay in absence of FRET and the FRET-induced donor quenching $\varepsilon_{D(A)}(t)$

$$F_{D(A)}(t) = F_{D(0)}(t) \cdot \varepsilon_{D(A)}(t). \quad (33)$$

We relate the FRET-induced donor quenching to the DA-distance distribution by the rate-constant of energy transfer as defined by Förster

$$k_{RET} = k_F \cdot \kappa^2 \cdot \left(\frac{R_{0J}}{R_{DA}}\right)^6 \quad (34)$$

Here, $R_{0J}$ is a reduced Förster-radius, $k_F$ is the radiative rate constant of fluorescence and $\kappa^2$ is the orientation-factor. This reduced Förster-radius is given by

$$R_{0J} = \left[\frac{9(\ln 10)}{128\pi^5 \cdot N_A} \cdot \frac{J}{n^4} \cdot\right]^{\frac{1}{6}} = 0.2108 \cdot \text{Å} \cdot \left[\frac{1}{n^4} \cdot \left(\frac{J(\lambda)}{\text{mol}^{-1} \cdot \text{dm}^3 \cdot \text{cm}^{-1} \cdot \text{nm}^4}\right)\right]^{\frac{1}{6}}, \quad (35)$$

where $N_A$ is Avogadro's constant, $n$ is the refractive index of the medium and $J = \int f_D(\lambda) \cdot \varepsilon_A(\lambda) \cdot \lambda^4 \cdot d\lambda$ is the overlap integral between $f_D(\lambda)$, the donor emission spectrum and $\varepsilon_A(\lambda)$, the acceptor absorption spectrum. The FRET-induced donor decay relates to the distance distribution $p(R_{DA})$ by

$$\varepsilon_{D(A)}(t) = \int p(R_{DA}) \cdot \exp\left(-t \cdot \langle \kappa^2 \rangle \cdot k_F \left[1 + (R_{0J}/R_{DA})^6\right]\right) dR_{DA}. \quad (36)$$

We use an average orientation factor of $\langle \kappa^2 \rangle \approx 2/3$ (justified by the anisotropy studies compiled in Supplementary Tables 3A-D). We used a reduced Förster-radius of $R_{0J}$ =56.4 Å which was determined for the donor with a radiative rate constant $k_F$=0.224 ns$^{-1}$. As previously described [25] we propagate potential errors of the $\langle \kappa^2 \rangle \approx 2/3$ approximation to our experimental distances (Supplementary Table 5d).

We use a superposition of normal distributions to describe a mixture of states:

$$p(R_{DA}) = \sum_{i=1}^{N} x_{DA}^{(i)} \frac{1}{w_{\mathrm{DA}}\sqrt{\pi/2}} \exp\left(-2\left[\frac{R_{\mathrm{DA}} - \langle R_{DA}^{(i)} \rangle}{w_{DA}}\right]^2\right). \quad (37)$$

Here, $N$ is the number of states (2 or 3) with $\langle R_{DA}^{(i)} \rangle$ being the mean of the state *(i)* distance distribution with species fraction $x_{DA}^{(i)}$ and a width $w_{DA}$ set to a physical meaningful value of 12 Å (flexible dye-linkers) [3].

We analyze our data by substituting Supplementary Equation (37) into (36). Next, Supplementary Equation (36) is inserted into Supplementary Equations (33). Finally, we analyze the fluorescence intensity decay of the donor in presence and absence of FRET (Supplementary Equation (38) or (39)) in a joint fit, in which the fluorescence lifetimes and corresponding species fractions of the donor only reference sample were identical to the respective parameters in the FRET sample. By this procedure the photon counting statistics of both the reference- and fluorescence-decay in presence of FRET is preserved. Thus, the counting statistics are clearly defined (Poisson distribution). This allows for an analysis with proper error-estimates. By the global (joint) analysis of the reference sample and the FRET-sample the photophysical properties (dynamic quenching) are taken into account. To further reduce the number of free model parameters, we combined the donor only and FRET measurements of all 33 FRET samples into a joint single data set, in which the species fractions of the DA-distance distribution were shared among all 33 variants. The so achieved reduction in degrees of freedom of a joint/global fit stabilizes the fit dramatically.

*Ensemble Time Correlated Single Photon Counting*

The experimental fluorescence intensity decays were fitted using the iterative re-convolution approach, where the model-decay curves are convoluted with the experimental instrument response function (*IRF*). Additionally, we consider a constant offset $c$ of the fluorescence intensity and correct the instrumental differential non-linearity by a time-dependent function $Lin(t)$. With these corrections, the experimental time-resolved fluorescence intensities of the FRET-sample and the donor reference sample are proportional to:

$$\begin{aligned} F_{\mathrm{FRET}}(t) &= \left(N_0 \cdot \left[(1 - x_{\mathrm{DOnly}}) F_{\mathrm{D(A)}}(t) + x_{\mathrm{DOnly}} F_{\mathrm{D(0)}}(t)\right] \otimes IRF + sc \cdot IRF + c\right) \cdot Lin(t) \\ F_{\mathrm{Ref}}(t) &= \left(N_0 \cdot F_{\mathrm{D(0)}}(t) \otimes IRF + sc \cdot IRF + c\right) \cdot Lin(t) \end{aligned} . \quad (38)$$

Here, $sc$ is due to scattered light from the sample. The model fluorescence intensity histograms were scaled to the experimental measured number of photons to reduce the number of free fitting parameters (the initial amplitude $N_0$ is not fitted).

*Sub-ensemble Time Correlated Single Photon Counting*

In sm-measurements we determine the number of fluorescent photons $N_F$ and the number of background photons $N_{BG}$ using buffer reference measurements as reference. Given the known number of fluorescence and background photons the fluorescence decays were modeled by:

$$\begin{aligned} F_{\mathrm{FRET}}(t) &= N_F \cdot \left[(1 - x_{\mathrm{DOnly}}) F_{\mathrm{D(A)}}(t) + x_{\mathrm{DOnly}} F_{\mathrm{D(0)}}(t)\right] \otimes IRF + N_{BG} \cdot IRF \\ F_{\mathrm{Ref}}(t) &= N_F \cdot F_{\mathrm{D(0)}}(t) \otimes IRF + N_{BG} \cdot IRF \end{aligned} . \quad (39)$$

This procedure reduced the number of the number of free parameters compared to the eTCSPC measurements.

*Summary fit models*

In total, we used three different fit models to describe our data. They differ in their number of states and the number of joint (global) and free relevant parameters, which are given in Supplementary Table 5. The fit of the two globally linked states was obtained within the procedure to estimate the fraction of the third state (see below).

*Fitting of functional variants*

Functional variants were fitted globally, i. e. distances for states $C_1$ and $C_2$ were linked over all three variants used to mimic free enzyme *E*, enzyme-substrate complex *ES* and enzyme product complex *EP* while the distance for $C_3$ was only linked for *E* and *ES* to allow for the different (covalent) nature of this state in *EP*. The experimental fluorescence decays were fitted by the conventional Levenberg–Marquardt minimization algorithm using custom software written in Python.

*Uncertainty estimation*

The statistical errors of the DA-distances were determined by sampling the parameter space [26,27] and applying the F-distribution at a confidence level of 95% (2σ) given the minimum determined $\chi^2$. The maximum allowed $\chi^2_{r,\max}$ for a given confidence-level (*P;* e.g. for 2σ *P* = 0.95) was calculated by

$$\chi^2_{r,\max}(P) = \chi^2_{r,\min} \cdot \left[1 + n/\upsilon \cdot \mathrm{cdf}^{-1}(F(n,\upsilon,P))\right], \quad (40)$$

where $\mathrm{cdf}^{-1}(F(\mathrm{n},\nu,P))$ is the inverse of the cumulative distribution function of the *F*-distribution for *n* number of free parameters, and with ν degrees of freedom. $\chi_r^2{}_{\min}$ is the minimum determined $\chi_r^2$ [28].

To estimate the species fraction $x_3$ of the third state, we performed a support plane analysis for the global fit [29].

**Supplementary References**


1. Ratzke, C., Hellenkamp, B. & Hugel, T. Four-colour FRET reveals directionality in the Hsp90 multicomponent machinery. *Nat. Commun.* **5**, 4192 (2014).
2. Vale, R. D. & Oosawa, F. Protein motors and Maxwell's demons: does mechanochemical transduction involve a thermal ratchet? *Adv. Biophys.* **26**, 97-134 (1990).
3. Sindbert, S. *et al.* Accurate distance determination of nucleic acids via Forster resonance energy transfer: implications of dye linker length and rigidity. *J. Am. Chem. Soc.* **133**, 2463-2480 (2011).

4. Sisamakis, E., Valeri, A., Kalinin, S., Rothwell, P. J. & Seidel, C. A. Accurate single-molecule FRET studies using multiparameter fluorescence detection. *Methods Enzymol.* **475**, 455-514 (2010).
5. Kudryavtsev, V. *et al.* Combining MFD and PIE for accurate single-pair Forster resonance energy transfer measurements. *ChemPhysChem* **13**, 1060-1078 (2012).
6. Widengren, J. *et al.* Single-molecule detection and identification of multiple species by multiparameter fluorescence detection. *Anal. Chem.* **78**, 2039-2050 (2006).
7. Felekyan, S., Kalinin, S., Sanabria, H., Valeri, A. & Seidel, C. A. Filtered FCS: species auto- and cross-correlation functions highlight binding and dynamics in biomolecules. *ChemPhysChem* **13**, 1036-1053 (2012).
8. Gopich, I. V. & Szabo, A. Theory of the energy transfer efficiency and fluorescence lifetime distribution in single-molecule FRET. *Proc. Natl. Acad. Sci. USA* **109**, 7747-7752 (2012).
9. Maeda, H. A new lysozyme assay based on fluorescence polarization or fluorescence intensity utilizing a fluorescent peptidoglycan substrate. *J Biochem* **88**, 1185-1191 (1980).
10. Dale, R. E., Eisinger, J. & Blumberg, W. E. The orientational freedom of molecular probes. The orientation factor in intramolecular energy transfer. *Biophys. J.* **26**, 161-193 (1979).
11. Felekyan, S. *et al.* Full correlation from picoseconds to seconds by time-resolved and time-correlated single photon detection. *Rev. Sci. Instrum.* **76** (2005).
12. Sustarsic, M. & Kapanidis, A. N. Taking the ruler to the jungle: single-molecule FRET for understanding biomolecular structure and dynamics in live cells. *Curr. Opin. Struct. Biol.* **34**, 52-59 (2015).
13. Picoquant. <https://www.picoquant.com/images/uploads/page/files/14528/technote_tttr.pdf>
14. Maus, M. *et al.* An experimental comparison of the maximum likelihood estimation and nonlinear least-squares fluorescence lifetime analysis of single molecules. *Anal. Chem.* **73**, 2078-2086 (2001).
15. Kalinin, S., Sisamakis, E., Magennis, S. W., Felekyan, S. & Seidel, C. A. On the origin of broadening of single-molecule FRET efficiency distributions beyond shot noise limits. *J. Phys. Chem. B* **114**, 6197-6206 (2010).
16. Elson, E. L. & Magde, D. Fluorescence correlation spectroscopy. I. Conceptual basis and theory. *Biopolymers* **13**, 1-27 (1974).

17. Magde, D., Elson, E. L. & Webb, W. W. Fluorescence correlation spectroscopy. II. An experimental realization. *Biopolymers* **13**, 29-61 (1974).
18. Kim, S. A., Heinze, K. G., Bacia, K., Waxham, M. N. & Schwille, P. Two-photon cross-correlation analysis of intracellular reactions with variable stoichiometry. *Biophys. J.* **88**, 4319-4336 (2005).
19. Schwille, P. Vol. 65 (ed R. Rigler E.L. Elson) 360-378 (Springer, Berlin, 2001).
20. Böhmer, M., Wahl, M., Rahn, H. J., Erdmann, R. & Enderlein, J. Time-resolved fluorescence correlation spectroscopy. *Chem. Phys. Lett.* **353**, 439-445 (2002).
21. Felekyan, S., Sanabria, H., Kalinin, S., Kuhnemuth, R. & Seidel, C. A. Analyzing Forster resonance energy transfer with fluctuation algorithms. *Methods Enzymol.* **519**, 39-85 (2013).
22. Koshioka, M., Sasaki, K. & Masuhara, H. Time-Dependent Fluorescence Depolarization Analysis in Three-Dimensional Microspectroscopy. *Appl. Spectrosc.* **49**, 224-228 (1995).
23. Schaffer, J. *et al.* Identification of single molecules in aqueous solution by time-resolved fluorescence anisotropy. *J. Phys. Chem. A* **103**, 331-336 (1999).
24. Woźniak, A. K., Schröder, G., Grubmüller, H., Seidel, C. A. M. & Oesterhelt, F. Single molecule FRET measures bends and kinks in DNA. *Proc.Natl.Acad.Sci.USA.* **105**, 18337-18342 (2008).
25. Kalinin, S. *et al.* A toolkit and benchmark study for FRET-restrained high-precision structural modeling. *Nat. Meth.* **9**, 1218-1225 (2012).
26. Goodman, J. & Weare, J. Ensemble Samplers with Affine Invariance. *Comm. App. Math. Comp. Sci.* **5**, 65-80 (2010).
27. Foreman-Mackey, D., Hogg, D. W., Lang, D. & Goodman, J. emcee: The MCMC Hammer. *Publications of the Astronomical Society of the Pacific* **125**, 306-312 (2013).
28. Lakowicz, J. R. *Principles of Fluorescence Spectroscopy*. Third edn, (Springer, 2006).
29. Straume, M., Frasier-Cadoret, S. G. & Johnson, M. L. Topics in Fluorescence Spectroscopy, Principles. Vol. 2 (ed Joseph R Lakowicz) Ch. Least-Squares Analysis of Fluorescence Data, 177-240 (Plenum Press, 1991).
