# Supplementary material for: Resolving dynamics and function of transient states in single enzyme molecules
Source: Nat Commun. 2020 Mar 6;11:1231. doi: 10.1038/s41467-020-14886-w (PMC7060211; doi:10.1038/s41467-020-14886-w)
Supplement: Supplementary file 4 — Source Data [file 41467_2020_14886_MOESM4_ESM.pdf]

Source Data File

Source Data to Supplementary Figure 8C

20121219 incubation of t26e.jpg

1 2 3 4 5 6 7 8 9

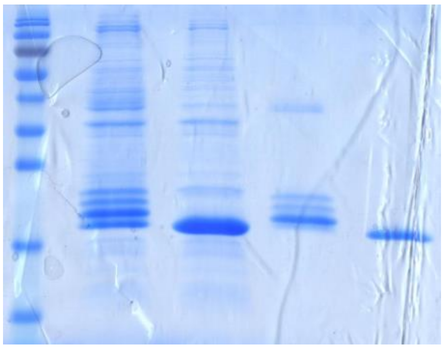

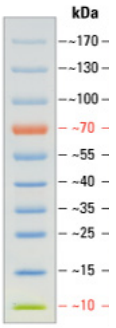

Lanes from left to right:

1) Marker: Thermo Scientific™ PageRuler™ Prestained 10-180kDa Protein Ladder
2) (empty)
3) T4L S44pAcF I150C, 0 hrs
4) (empty)
5) T4L S44pAcF I150C, 2 hrs
6) (empty)
7) T4L N55pAcF N132C, 0 hrs
8) (empty)
9) T4L N55pAcF N132C, 2hrs
